# Bioactive peptides and proteins for tissue repair: microenvironment modulation, rational delivery, and clinical potential

**DOI:** 10.1186/s40779-024-00576-x

**Published:** 2024-12-05

**Authors:** Zhuo-Wen Hao, Zhe-Yuan Zhang, Ze-Pu Wang, Ying Wang, Jia-Yao Chen, Tian-Hong Chen, Guang Shi, Han-Ke Li, Jun-Wu Wang, Min-Chao Dong, Li Hong, Jing-Feng Li

**Affiliations:** 1 Department of Orthopedics, Zhongnan Hospital of Wuhan University, Wuhan, 430071 China; 2 Department of Obstetrics and Gynecology, Renmin Hospital of Wuhan University, Wuhan, 430060 China

**Keywords:** Bioactive peptides and proteins (BAPPs), Growth factors, Delivery strategies, Tissue regeneration, Clinical potential

## Abstract

Bioactive peptides and proteins (BAPPs) are promising therapeutic agents for tissue repair with considerable advantages, including multifunctionality, specificity, biocompatibility, and biodegradability. However, the high complexity of tissue microenvironments and their inherent deficiencies such as short half-live and susceptibility to enzymatic degradation, adversely affect their therapeutic efficacy and clinical applications. Investigating the fundamental mechanisms by which BAPPs modulate the microenvironment and developing rational delivery strategies are essential for optimizing their administration in distinct tissue repairs and facilitating clinical translation. This review initially focuses on the mechanisms through which BAPPs influence the microenvironment for tissue repair via reactive oxygen species, blood and lymphatic vessels, immune cells, and repair cells. Then, a variety of delivery platforms, including scaffolds and hydrogels, electrospun fibers, surface coatings, assisted particles, nanotubes, two-dimensional nanomaterials, and nanoparticles engineered cells, are summarized to incorporate BAPPs for effective tissue repair, modification strategies aimed at enhancing loading efficiencies and release kinetics are also reviewed. Additionally, the delivery of BAPPs can be precisely regulated by endogenous stimuli (glucose, reactive oxygen species, enzymes, pH) or exogenous stimuli (ultrasound, heat, light, magnetic field, and electric field) to achieve on-demand release tailored for specific tissue repair needs. Furthermore, this review focuses on the clinical potential of BAPPs in facilitating tissue repair across various types, including bone, cartilage, intervertebral discs, muscle, tendons, periodontal tissues, skin, myocardium, nervous system (encompassing brain, spinal cord, and peripheral nerve), endometrium, as well as ear and ocular tissue. Finally, current challenges and prospects are discussed.

## Background

Bioactive peptides and proteins (BAPPs) are functional molecules with unique amino acid sequences that are involved in a plethora of biological processes, including biocatalysis, immunomodulation, activation/inhibition of signaling pathways, and the regulation of cellular fate and behavior [1, 2]. Thus, they show great potential as therapeutic agents for various diseases, such as diabetes [3], tissue damage [4], cancer [5], infection [6], and chronic pain [7]. Since the first commercial success of human insulin (Humulin) in 1982, over 80 BAPPs have been approved and administered worldwide in clinics, with more than 150 candidates currently undergoing clinical development and an additional 400 − 600 candidates in preclinical studies [8]. It is estimated that the pharmaceutical market for BAPPs will surpass that for small-molecule drugs, reaching approximately $400 billion by 2025 [9]. With the development of omics [10], display technologies [11], computational modeling [12], and machine learning [13], therapeutics based on BAPPs are expected to grow exponentially.

Tissue repair is generally correlated with acute or chronic injuries, degenerative diseases, and metabolic diseases. BAPPs serve as potent therapeutic agents for tissue regeneration, offering distinct advantages. In comparison to other bioactive compounds, such as small molecule drugs [14], nucleic acids [15], ions [16], nanoparticles [17], and micro/nanostructures [18], BAPPs target and interact with specific receptors with limited side effects and have highly complex functions [19, 20]. Additionally, BAPPs exhibit intrinsic biocompatibility and biodegradability due to their natural presence in the tissue microenvironment where they engage in biological processes [21, 22]. These attributes enable BAPPs to modulate the microenvironment via reactive oxygen species (ROS), blood and lymphatic vessels, immune cells, and repair cells for customizable tissue repair at specific anatomical sites.

While BAPPs induce multiple functions to modulate complex microenvironments for tissue regeneration, their therapeutic efficacy remains limited primarily due to their short half-life and susceptibility to enzymatic degradation [23]. Furthermore, the degree of effect exerted by BAPPs is firmly related to both loading concentration and release kinetic [4]. Therefore, innovative delivery platforms have been developed to incorporate BAPPs, preserving their bioactivity and shielding them from enzymatic breakdown [24]. Reasonable modifications to these delivery systems can improve loading efficiency while enabling rapid controlled, sustained, or heterogeneous release. The strategic combination of diverse delivery platforms allows the sequential release of multiple BAPPs for tissue repair.

To address the intricate requirements of physiological tissue repair during distinctive periods, the on-demand release of BAPPs by stimuli-responsive delivery systems has attracted substantial attention in the biomedical field [25]. The release of BAPPs can be triggered by endogenous stimuli from the microenvironment, such as glucose, ROS, enzymes, and pH. Moreover, exogenous stimuli such as ultrasound, heat, light, magnetic field, and electric field could be artificially applied to control the release of BAPPs for tissue regeneration. Based on the stimuli-responsive delivery, BAPPs could be precisely released to facilitate the process of tissue repair, thus achieving targeted administration of these agents.

**Fig. 1:**
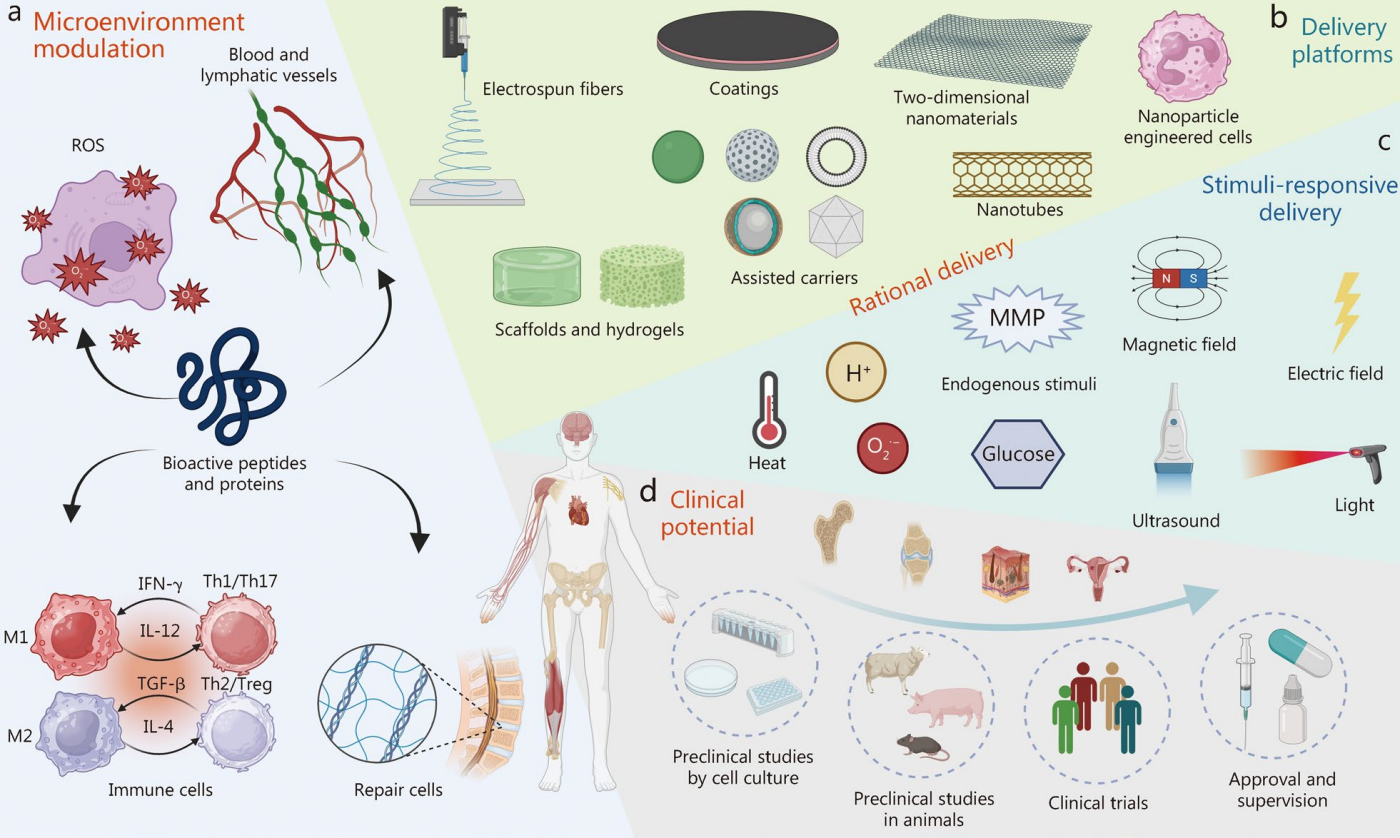
Overview of bioactive peptides and proteins (BAPPs). **a** Microenvironment modulation, including ROS, blood and lymphatic vessels, immune cells, and repair cells. **b** Delivery platforms comprising electrospun fibers, coatings, two-dimensional nanomaterials, nanoparticles, engineered cells, scaffolds, hydrogels, assisted carriers, and nanotubes. **c** Stimuli-responsive delivery mechanisms involving endogenous stimuli, magnetic field, electric field, heat, light, and ultrasound. **d** Clinical potential encompassing preclinical studies utilizing cell culture and animal models alongside clinical trials, as well as approval and supervision. ROS reactive oxygen species, MMP matrix metalloproteinases, IL interleukin, IFN-γ interferon-γ, Th helper T cell, Treg regulatory T cells, TGF-β transforming growth factor-β

Herein, we review the basic mechanisms by which BAPPs modulate the microenvironment, alongside recent progress in rational delivery strategies and their clinical potential for tissue repair (Fig. 1). Two critical dimensions should be considered when delivering BAPPs for tissue repair: the selection of appropriate BAPPs and the choice of delivery strategies. The selection of BAPPs is closely linked to their functions and target tissues, as well as specific characteristics of the tissue microenvironment. Regarding delivery strategies, it is essential to consider either single or multiple factor release through various platforms, with an emphasis on spatiotemporal control for stimuli-responsive delivery. Moreover, we focus on the clinical potential of BAPPs by evaluating their efficacy in clinical trials or preclinical studies. This review aims to elucidate how BAPPs influence microenvironment modulation and identify distinctive rational delivery strategies that can promote clinical translation.

## Microenvironment modulation by BAPPs

Distinctive BAPPs play a crucial role in modulating the tissue microenvironment to promote tissue repair through various mechanisms, mainly involving ROS, blood and lymphatic vessels, immune cells, and repair cells. The ability of BAPPs to modulate the microenvironment is summarized in Table 1 [26–114]. Understanding microenvironment modulation will enhance the selection and subsequent application of specific BAPPs for effective tissue repair.

**Table 1:** Bioactive peptides and proteins (BAPPs) for microenvironment modulation

Main function	BAPPs	Receptor/Functional area	Pathway	Other function	Target cells	Target factors	References
ROS scavenging	Antioxidant-RP1(AMRLTYNKPCLYGT)	-	-	-	-	DPPH↓ ABTS↓	[26]
	PaT-2	-	-	-	-	ROS↓	[27]
	L-carnosine (C_9_H_14_N_4_O_3_) Carnosine-Hy	His	Nrf2-Are-HO1	Anti-aging	-	-OH ↓ ROS↓ RNS↓ RCS↓	[28]
	CAQAPLA	-	-	-	-	-OH↓ DPPH↓ ABTS↓	[29]
	SS-31 (d-Arg-Dmt-Lys-Phe-NH_2_)	Dmt	-	-	-	ROS↓	[30]
	C-peptide	-	Dmt	-	-	ROS↓	[31]
Angiogenesis	VEGF	VEGFR-2	PI3K-Akt, Ras-Erk1/2	Osteogenesis	ECs	-	[32]
	QK or KLT (KLTWQELYQLKYKGI)	VEGFR-2	PI3K-Akt, Ras-Erk1/2	Osteogenesis	ECs	-	[33]
	IGFs	IGF-1R	PI3K-Akt, Ras-Erk1/2	Osteogenesis	ECs, VSMCs, pericytes	-	[34, 35]
	IGF-1derived peptide (GYGSSSRRAPQT)	IGF-1R	PI3K-Akt, Ras-Erk1/2	Osteogenesis	ECs, VSMCs, pericytes	-	[36, 37]
	FGF-2	FGFR	Ras-Erk1/2	Skin regeneration	ECs	-	[38, 39]
	PDGF	PDGFR-β	PI3K-Akt, Ras-Erk1/2	Osteogenesis	VSMCs, pericytes	-	[40, 41]
	TGF-β	TGFBR1(ALK5), ACVRL1(ALK1)	Smad, p38(MAPK)	-	ECs, VSMCs, pericytes	-	[42, 43]
Lymphatic regeneration	ET-1, VEGF	ETBR, VEGFR-3	-	-	LECs	-	[44]
Immune modulation	LSALT	DPEP-1	-	-	Neutrophils	-	[45]
	MLIF (Met-Gln-Cys-Asn-Ser)	Gln-Cys-Asn	VLA-4, VCAM-1	-	Monocytes	-	[46]
	IFN-γ	IFN-γR	JAK2-STAT1	-	M1 macrophage	-	[47]
	TNF-ɑ	TNFR	-	-	M1 macrophage	-	[47]
	GM-CSF	-	JAK2-STAT5	-	M1 macrophage	-	[48]
	PGRN, atsttrin peptide	TNFR	NF-κB, p38(MAPK), JNK-MAPK	-	M1 macrophage	-	[49]
	APETx2 (GTACSCGNSKGIYWFYRPSCPTDRGYTGSCRYFLGTCCTPAD)	ASIC-3	NF-κB, p38(MAPK)	-	M1 macrophage	IL-1↓ IL-6↓ TNF-α↓ MMP3↓ ADAMATs↓ COL2↑ AGGRECA↑	[50]
	MLIF (Met-Gln-Cys-Asn-Ser) MLIF related peptides (Gln-Cys-Asn-Ser)	Gln-Cys-Asn, Try, His	NF-κB, MAPK	-	M1 macrophage	MIP-1α↓ MIP-1β↓ IL-1β↓ IL-8↓ CCK1↓ TNF-α↓ CCR1↓	[51]
HBD1 PEP-B (ACPIFTKIQGTCYRG)	Electrostatic bonding	NF-κB, MAPK	-	-	IL-6↓ TNF-α↓ IL-8↓ TLR-2↓ TLR-4↓ mRNA↓		[52, 53]
	IL-4, IL-10	IL-4R, IL-10R	JAK1/2-STAT6, PI3K-Akt, JNK	-	M2 macrophage	-	[54]
	CD200	-	PI3K-Akt, NF-κB	-	M2 macrophage	TNF-α↓ IL-6↓ IL-10↑	[55]
	VIP	VIP-R	-	-	Th2 cell	T-bet↓ CXCL10↓ c-MAF↑ GATA-3↑ JUN-B↑ CCL22↑	[56]
	MILF (Met-Gln-Cys-Asn-Ser)	Gln-Cys-Asn	-	-	Th2 cell, CD8^+^ T cell	IL-10↑	[57]
Cell survival	BCL-2	BH4	-	-	-	-	[58]
	MCP-1	-	Caspase-3↓	-	H9C2 myofibroblasts	-	[59]
	7A (MHSPGAD)	-	-	-	H9C2 cells	-	[60, 61]
	OspC-3	-	GSDMD-caspase-4/11	-	-	-	[62]
	FSP-1	-	-	-	-	COQ10↓ Vitamin K↓ Lipid ROS↓	[63]
Cell senescence	AnkB-LIR	-	Atg8 family	-	-	-	[64]
	AnkG ER	-	GABARAP selective	-	-	-	[64]
	PTH 1-34	-	mTOR	-	-	-	[65, 66]
	Transforming growth factor-β1 (TGF-β1)	-	Smad	-	-	p15↓ p21↓ p27↓ c-Myc↓	[67]
	BMP-4	-	-	-	-	p16↓ p53↓	[68]
	IGFBP7	-	SIRT1 deacetylase	-	-	p21↓	[69]
	Stromal cell derived factor-1 (SDF-1)	CXCR-4	PI3K-Akt	-	MSCs, EPCs	-	[70]
Cell recruitment	E7 peptide (EPLQLKM)	CXCR-4	PI3K-Akt, Ras-Erk1/2, p38(MAPK)	-	BMSCs	-	[71, 72]
	BMHP1 (PFSSTKT), BMHP2 (SKPPGTSS)	-	-	-	BMSCs	-	[73, 74]
	Substance P	NK-1R	Ras-Erk1/2	-	MSCs	-	[75]
Cell adhesion	RGD						
(Arg-Gly-Asp)	Integrin-αvβ3	-	Osteogenesis, angiogenesis	MC3T3-E1, MSCs, HUVECs	-	[76]	
	DGR	-	-	Osteogenesis	MC3T3-E1 cells	-	[77]
	PHSNR	-	-	Osteogenesis	Fibroblast	-	[78]
	NCAM sequence (EVYVVAENQQGKSKA)	IgSF	-	-	NSCs	-	[79]
	DGEA, GFOGER, GTPGPQGIAGQRGVV	α2β1	-	-	Osteoblasts	-	[80]
	REDV, SVVYGLR	α4β1	-	-	ECs	-	[80]
Osteogenesis	P20 (KIPKASSVPTELSAISTLYL)	-	-	-	-	-	[81]
	P24 (pSKIPKASSVPTELSAISTLYLDDD)	-	-	-	-	-	[82]
	BMP-2-mimetic-peptide (SpSVPTNSPVNSKIPKACCVPTELSAI)	BMPRI	Smad, Wnt	Angiogenesis	BMSCs	-	[83, 84]
	BMP-4-mimetic peptide (RKKNPNCRRH)	-	-	-	-	-	[85]
	BMP-7-mimetic peptide (GQGFSYPYKAVFSTQ)	-	-	-	-	-	[86]
	BMP-9-mimetic peptide (CGGKVGKACCVPTKLSPISVLYK)	-	-	-	-	-	[87]
	Parathyroid hormone (PTH)	-	-	-	-	-	[88]
	Teriparatide (SVSEIQLMHNLGKHLNSMERVEWLRKKLQDVHNF)	-	-	-	-	-	[88]
	PTHrP 1 (pSVSEIQLMHNLGKHLNSMERVEWLRKKLQDVHNFDD)	PTHIR	CAMP^_^PKA, PKC, WNT, Ras-Erk1/2, p38(MAPK), NLK-MAPK	Angiogenesis	MSCs	-	[88]
	PTHrP2 (pSVSEIQLMHNLGKHLNSMERVEWLRKKLQDVHNFEEE)	-	-	-	-	-	[88]
	PTH related protein	-	-	-	-	-	[89, 90]
	Abaloparatide	-	-	-	-	-	[91, 92]
	OGP (ALKRQGRTLYGFGG), OGP10-14 (YGFGG)	-	HO-1-eNOS, CDK2-cyclin A, RhoA/ROCK, lK141205-CXCL13, Ras^_^Erk1/2	-	MSCs, osteoblasts	-	[93]
	BIFP, BIFY	RANK, RANKL	p-Akt, NFATc1	-	Osteoclasts	-	[94]
	PHTrP107-111 (osteostatin)	-	PKC-RANKL/M-CSF-NFATC1, cathepsin K, OSCAR	Angiogenesis	Osteoclasts	-	[95, 96]
Cartilage regeneration	Transforming growth factor-β (TGF-β), Fibroblast growth factor-18 (FGF-2), LIANAK, YYVGRKPK	-	PI3K-Akt, Ras-Erk1/2	-	Joint synovium MSCs, embryonic stem cells, IPSCs	-	[97, 98]
Intervertebral disc regeneration	IGF-1, TGF-β, SDF-1, CCL-5	-	Tie2-ANG-1	-	Tie2^+^GD2^+^ cells, ESCs	-	[99–101]
Muscle regeneration	HGF	C-MET	PI3K-Akt	-	SCs	MyoD↑ myogenin↑ IGF-binding↑	[102]
	IGF-1	IGF-1R	PI3K-Akt	-	SCs	MyoD↑ myogenin↑ IGF-binding↑	[102]
Tendon regeneration	IGF-1, BMP-12, POSTN	-	TGF-β-Smad2/3, PI3K-Akt	-	TSPCs	-	[103, 104]
Periodontal tissue repair	PDGF-BB, FGF-2, BMP-2	-	-	-	PDLSCs, odontoblast	-	[105, 106]
Skin regeneration	EGF, PDGF	-	PI3K-Akt, JAK-STAT, Ras-Erk1/2	Angiogenesis	ESCs, fibroblasts	-	[107, 108]
Myocardial regeneration	PDCD5	-	HDAC-3-TGF-β-SMAD	-	Fibroblasts	HDAC-3↓	[109]
Nerve regeneration	Brain-derived neurotrophic factor (BDNF)	Tropomyosin receptor kinase B	PI3K-Akt, Ras-Erk, PLCγ1-PKC	-	NSCs	-	[110]
	Neural growth factor (NGF)	TrkA/ p75NTR	Ras-Erk1/2-Elk1/Rsk, PI3K-Akt-(BAX-BAD)	-	NSCs	-	[108]
	Calcitonin-gene related peptide (CGRP) (ACDTATCVTHRLAGLLSRSGGVVKNNFVPTNVGS)	RAMP1, CRLR	JNK, PI3K-Akt, Ras-Erk1/2	Angiogenesis	Schwann cells, MSCs	-	[111]
	LAR peptide	-	-	-	-	CSPG↓	[112]
	Ily-lys-val-Ala-Val (IKVAV)	-	-	-	Neural precursor cells	-	[113]
Endometrium tissue repair	KGF	-	-	-	ECs	-	[114]

*ROS* reactive oxygen species, *RNS* reactive nitrogen species, *RCS* active chlorine, *VEGF vascular endothelial growth factor VEGFR* vascular endothelial growth factor receptor, *ECs* endothelial cells, *VSMCs* vascular smooth muscle cells, *LECs* lymphatic endothelial cells, *MSC* mesenchymal stem cell, *LSALT* proper noun, *MLIF* monocyte locomotion inhibitory factor, *IFN-γ* interferon-γ, *GM-CSF* granulocyte–macrophage colony-stimulating factor, *PGRN* progranulin, *NF-κB* nuclear factor kappa-B, *MAPK* mitogen-activated protein kinase, *IL* interleukin, *VIP* vasoactive intestinal peptide, *BCL-2* B-cell lymphoma-2, *MCP-1* monocyte chemotactic protein-1, ESCs embryonic stem cells, *HDACs* histone deacetylase, *GSDMD* gasdermin D, *PTH* parathyroid hormone, *mTOR* mammalian target of rapamycin, *BMP* bone morphometric proteins, *IGFBP-7* IGF binding protein-7, *IGF-1* insulin-like growth factor-1, *PDCD5* programmed cell death 5, *SDF-1* stromal cell-derived factor-1, *CCL-5* C–C motif chemokine ligand 5, *HGF* hepatocyte growth factor, *PDGF-BB* platelet-derived growth factor-BB, *FGF-2* fibroblast growth factor-2, MIP-1α macrophage inflammatory protein-1α, *CCK* cell counting kit, *CCR* chemokine receptor, *PI3K-Akt* phosphoinositide 3-kinase-protein kinase B, *KGF* keratinocyte growth factor, *NSCs* neural stem cells, *CSPG* chondroitin sulfate proteoglycans, *PDLSCs* periodontal ligament stem cells, *TSPCs* tendon stem/progenitor cells, *IPSCs* induced pluripotent stem cells, *BIFP* binding-induced fibrillogenesis peptide P, *BIFY* binding-induced fibrillogenesis peptide Y

### ROS

**Fig. 2:**
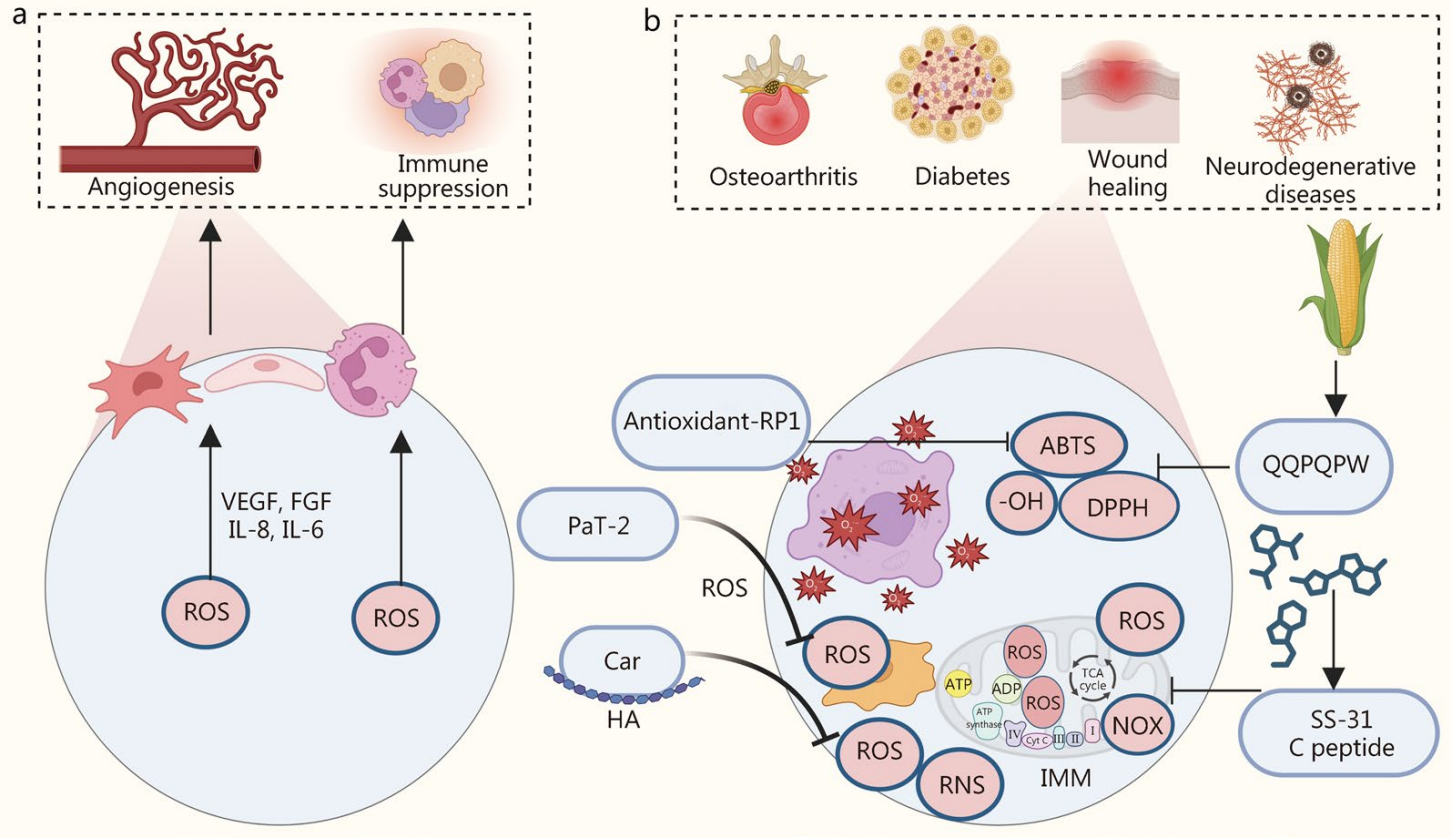
BAPPs regulate the ROS family during physiological and pathological processes.** a** Physiological function of reactive oxygen species (ROS). **b** Bioactive peptides and proteins (BAPPs) for scavenging ROS and other free radicals in osteoarthritis, diabetes, wound healing, and neurodegenerative diseases. VEGF vascular endothelial growth factor, FGF fibroblast growth factors, IL interleukin, PaT-2 FPPWL-NH2, HA hyaluronic acid, RNS reactive nitrogen species, NOX NADPH oxidases, ATP adenosine 5’-triphosphate, ADP adenosine diphosphate, TCA tricarboxylic acid cycle, Cyt C cytochrome C

ROS are a large class of oxidants derived from molecular oxygen, including reactive nitrogen, sulfur, carbon, selenium, electrophiles, and halogens [115]. At physiological concentrations in the tissue microenvironment, ROS play a pivotal role in tissue repair, mainly through mechanisms such as angiogenesis and immune suppression [116, 117], as illustrated in Fig. 2a. However, under certain pathological conditions such as hypoxia and hyperglycemia, there is an overproduction or accumulation of ROS. This excess leads to detrimental oxidative stress that compromises cellular proteins, lipids, and DNA integrity [118]. Consequently, mitigating excessive ROS levels and regulating the oxidative/antioxidant balance are essential for tissue repair. Certain bioactive peptides, known as antioxidant peptides (AOPs), exhibit potent antioxidant capacities. These compounds can effectively reduce the levels of ROS and pro-oxidants that mediate ROS production, thereby significantly delaying or preventing oxidative stress (Fig. 2b).

A diverse array of AOPs obtained from animals and plants are recognized for their environmental friendliness, low toxicity, and high antioxidant capacity [119]. Among the AOPs sourced from animals, carnosine is an endogenous dipeptide composed of two amino acids (β-alanine and L-histidine) and predominantly found in skeletal muscle [120]. The bioactive component of carnosine is its histidine residue, which can interact with and scavenge ROS [28]. Due to its potent antioxidative stress effects, carnosine has demonstrated promising therapeutic outcomes in conditions such as infectious and rheumatic polyarthritis, gastrointestinal ulcers, diabetes mellitus and cardiovascular diseases, Parkinson’s disease, and periodontitis [121]. However, when administered alone via injection in vivo, the efficacy of carnosine is greatly hampered by hydrolysis mediated by serum and tissue peptidases [122, 123]. Therefore, Lanza et al. [124] conjugated carnosine with hyaluronic acid (HA) to enhance both its antioxidant properties and resistance to enzymatic hydrolysis. Alternatively, antioxidant-RP1 (AMRLTYNKPCLYGT) is a naturally occurring AOP isolated from Rana pleuraden and found to effectively scavenge 1,1-diphenyl-2-picryl-hydrazyl radical (DPPH) and/or 2,2'-azinobis-(3-ethylbenzthiazoline-6-sulphonate) (ABTS) free radicals [26]. PaT-2 (FPPWL-NH2), another AOP derived from Pithecopus Azureus, has been shown to significantly block lipopolysaccharide (LPS)-induced glutamate release as well as ROS production in human microglia [27]. Among plant-derived AOPs, those obtained from maize exhibited notable antioxidant activity [125]. In prior research utilizing two proteases (Alcalase and Protamex) to hydrolyze maize gluten meal for AOP extraction, the amino acid sequence identified was Cys-Ser-Gln-Ala-Pro-Leu-Ala (CSQAPLA) [29], which displayed antioxidant activity against ABTS, DPPH, and -OH, demonstrating dose-dependent scavenging effects alongside certain superoxide radical scavenging abilities [29].

Among the chemically synthesized AOPs, SS-31 (d-Arg-Dmt-Lys-Phe-NH2) exerts antioxidant effects primarily attributed to the Dmt residue, enabling it to selectively target mitochondria and localize in the inner mitochondrial membrane [30]. Research indicates that this peptide is highly effective in inhibiting intracellular ROS and preventing the generation of oxidative stress [126]. Furthermore, a synthetic C-peptide has been shown to directly reduce ROS production by acting at subcellular sites and inhibiting ROS generation through its influence on high glucose (HG) activation of nicotinamide adenine dinucleotide phosphate oxidase at the plasma membrane, while also restoring normal mitochondrial electron transport chain function in endothelial cells (ECs) [31]. Consequently, these AOPs hold great potential for tissue repair due to their capacity to modulate elevated ROS levels in the microenvironment.

### Blood and lymphatic vessels

**Fig. 3:**
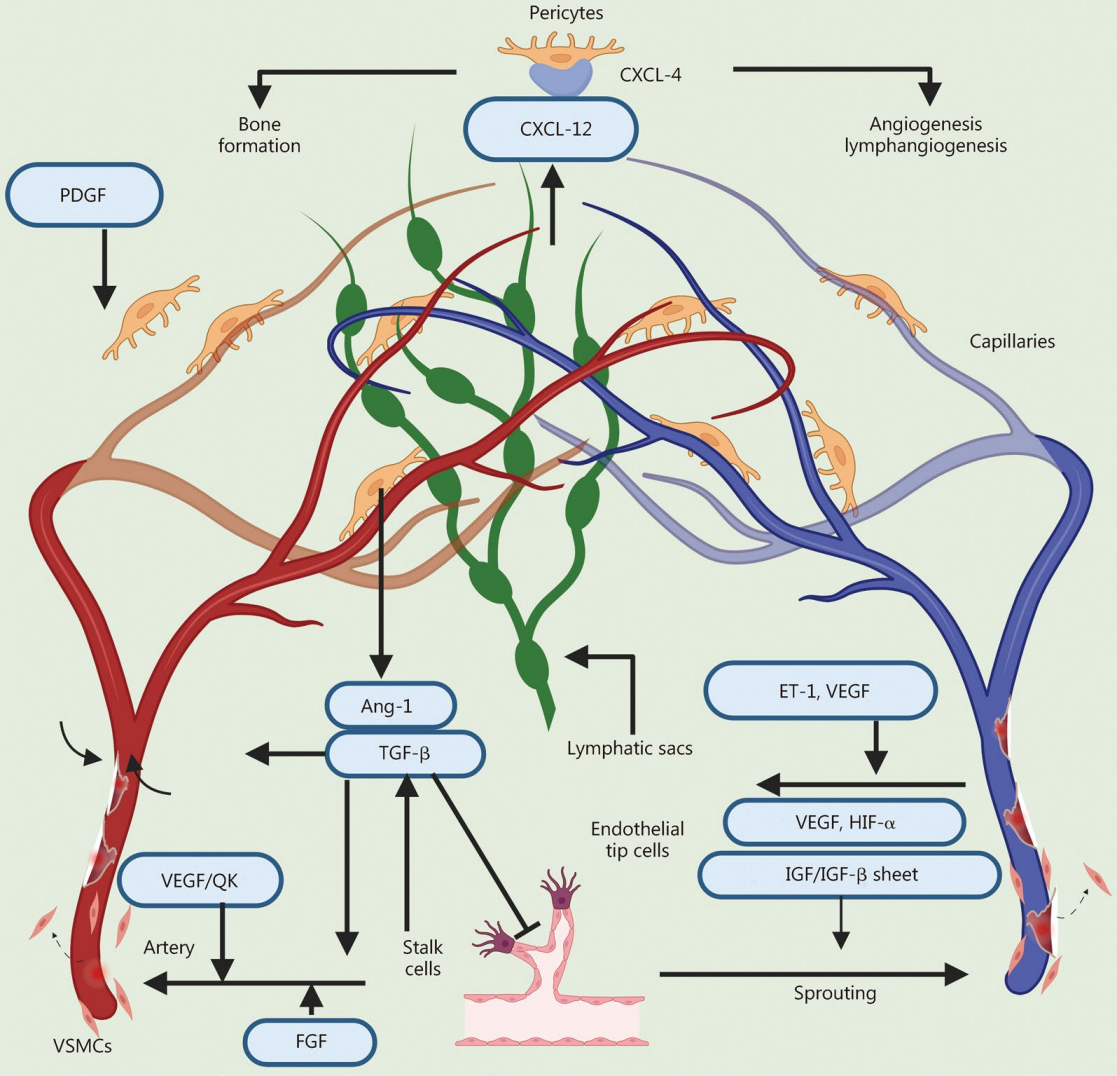
Bioactive peptides and proteins (BAPPs) for blood and lymphatic vessel regeneration. PDGF platelet-derived growth factor, Ang-1 angiopoietins-1, TGF-β transforming growth factor-β, VEGF vascular endothelial growth factor, QK KLTWQELYQLKYKGI, FGF fibroblast growth factors, VSMCs vascular smooth muscle cells, ET-1 endothelin-1, HIF-α hypoxia-inducible factor-α, IGF insulin-like growth factor, CXCL chemokine ligand C-X-C motif chemokine ligand

Blood and lymphatic vessels establish extensive networks essential for transporting fluids, gases, macromolecules and cells. Tissue repair generally requires the prior formation of a vascularized network, with exceptions for certain specific avascular tissues such as cartilage and intervertebral disks. This section summarizes the BAPPs that promote angiogenesis and lymphatic regeneration (Fig. 3).

Vascular endothelial growth factor (VEGF) is the predominant and critical angiogenic protein, binding to its receptor on ECs to promote their proliferation and migration by activating the phosphoinositide 3-kinase-protein kinase B (PI3K-Akt) signaling pathway and the Ras-extracellular signal-regulated kinases1/2 (Ras-Erk1/2) signaling pathway. In the later stages of angiogenic, elevated levels of VEGF are used to stabilize neo-vessels and extend the duration of angiogenic signaling [32]. A VEGF-derived peptide known as KLTWQELYQLKYKGI (QK) was developed based on helix regions 17 − 25 involved in receptor interaction, demonstrating similar capabilities in inducing EC proliferation and migration [33]. Insulin-like growth factor-1 (IGF-1) also activates the PI3K-Akt and Ras-Erk1/2 signaling pathways through binding to receptors, increasing the expression of VEGF to further facilitate the proliferation and migration of ECs [34]. Additionally, IGF-1 stimulates the proliferation, migration, and differentiation of vascular smooth muscle cells (VSMCs) and pericytes, which in turn promotes the construction and maintenance of vascular wall tissue and ensures the stabilization of neovascularization [35]. The C domain of IGF-1 (GYGSSSRRAPQT) exhibited the superior ability to promote angiogenesis during tissue repair processes [36, 37]. Fibroblast growth factors (FGFs), a family of pleiotropic factors acting on various cell types including ECs, show potent angiogenic function via the Ras-Erk1/2 pathway after binding to the receptor [38, 39]. Moreover, transforming growth factor-β (TGF-β), another bioactive protein involved in the proliferation and migration of ECs regulates the differentiation of VSMCs by upregulating the expression of α-smooth muscle actin and smooth muscle myosin through drosophila mothers against decapentaplegic 3 (Smad3) and p38-mitogen-activated protein kinase (MAPK) pathways [42, 43]. Platelet-derived growth factor (PDGF) can recruit VSMCs and pericytes to modulate stable proliferation and differentiation of ECs during the late stage of angiogenesis [40, 41].

Recent studies have revealed that lymphatic vessels possess broader tissue repair functions. For example, these vessels facilitate the recovery of cardiac tissue after injury by secreting the lymphatic endothelium-derived extracellular protein Reelin (RELN) [127, 128]. In the context of skin repair, lymphangiectasia ECs enhance lymphatic drainage and promote tissue regeneration through the secretion of the lymphatic vessel secretory factor chemokine ligand C-X-C motif chemokine ligand 12 (CXCL-12) and angiopoietin-like protein 7 [129]. Regarding bone tissue, research has indicated that lymphatic vessels are present in both mouse and human bones, with intraosseous lymphatic vessels playing a crucial role in human bone development and hematopoietic regeneration [130]. Therefore, employing BAPPs to stimulate the formation of lymphatic vessels may be a potential strategy for tissue repair, and the co-formation of blood and lymphatic vessels also holds significant potential. Endothelin-1 (ET-1) along with its receptor endothelin B receptor (ETBR) could serve as viable targets for inducing lymphangiogenesis in tissue repair. ET-1 acts on ETBR to directly regulate lymphatic EC proliferation, migration, invasion, and differentiation and also indirectly promotes VEGF induction. Under normoxic conditions, ET-1 upregulates the expression levels of VEGF-C, vascular endothelial growth factor-3 (VEGFR-3), and VEGF-A and stimulates hypoxia-inducible factor-1α expression, thereby regulating the process of lymphangiogenesis [44].

### Immune cells

**Fig. 4:**
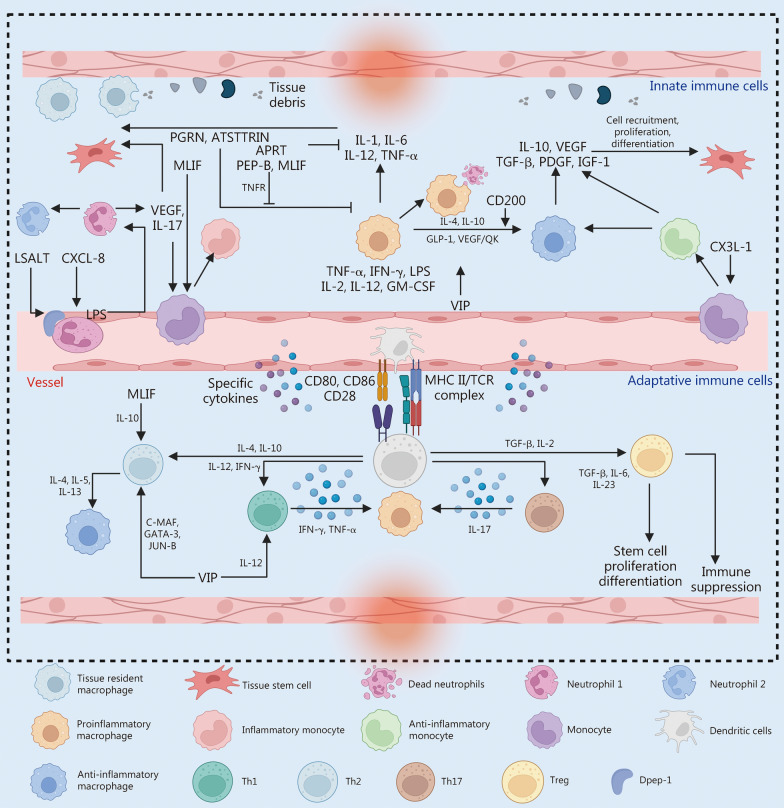
Bioactive peptides and proteins (BAPPs) for immune cell. For innate immune cells, BAPPs regulate the pro-inflammatory and anti-inflammatory balance of neutrophils, monocytes, and macrophages primarily by acting on these cells. In the case of adaptive immune cells, BAPPs similarly modulate the proinflammatory and anti-inflammatory balance of T cells with various phenotypes as well as macrophages by predominantly targeting these cells. PGRN progranulin, ATSTTRIN proper noun, APET ASIC3 inhibitory peptide, MLIF monocyte locomotion inhibitory factor, VEGF vascular endothelial growth factor, QK KLTWQELYQLKYKGI, IL interleukin, LASLT proper noun, LPS lipopolysaccharide, PEP-B synthetic human β-defensin 1 short motif, TNFR tumor necrosis factor receptor, TNF tumor necrosis factor, IFN interferon, GM-CSF granulocyte–macrophage colony-stimulating factor, GLP glucagon-like peptide, VIP vasoactive intestinal polypeptide, IGF insulin-like growth factor, PDGF platelet-derived growth factor, CX3L the chemokine (C-X-C motif) ligand, MHC major histocompatibility complex, TCR T cell antigen receptor, Th helper T cell, Treg regulatory T cells, DPEP dipeptidase

A range of innate and adaptive immune cell subtypes is instrumental in tissue repair and regeneration, so it is essential to incorporate a broad spectrum of immunomodulatory BAPPs in the design of repair therapies, and precisely regulate immune cell activation and recruitment to create an inflammatory microenvironment that promotes tissue repair (Fig. 4).

#### Innate immune cells

In the early stages of tissue injury, neutrophil influx is triggered by inflammation, but excessive recruitment of neutrophils can lead to an exaggerated inflammatory response. Therefore, optimal modulation of neutrophil recruitment represents a primary focus for intervention. LSALT peptide, a non-enzymatic dipeptidase-1 inhibitor, effectively obstructs neutrophil migration to the site of injury during acute inflammation, thus reducing inflammation and accelerating tissue regeneration [45]. Additionally, natural homeostasis between proinflammatory N1 and anti-inflammatory N2 phenotypes emerges in the inflammatory environment. Identifying and developing BAPPs aimed at targeting neutrophil polarization may be a potential immunomodulatory strategy for tissue repair by shifting the immune response to a pro-regenerative state.

Monocytes are recruited to the damaged site by VEGF and interleukin (IL)-17 produced by neutrophils, playing a vital role in tissue repair [131]. The process of tissue repair may be impeded if the recruitment of monocytes is inhibited; conversely, overrecruited monocytes can result in heightened inflammation due to the subsequent formation of macrophages [132]. Thus, appropriate regulation of recruitment is important for tissue repair. Monocyte locomotion inhibitory factor (MLIF) is an oligopeptide consisting of 5 amino acids (Met-Gln-Cys-Asn-Ser), and the Gln-Cys-Asn motif is the functional region [133]. MLIF primarily exerts its immunomodulatory effects by inhibiting adhesion molecules very late antigen-4 (VLA-4) and vascular cellular adhesion molecule 1 (VCAM-1) on mononuclear phagocytes, thereby diminishing monocyte migration and mitigating the inflammatory response during the initial phase of injury [46].

Monocyte-derived macrophages represent a crucial immune cell type with multiple functions and play essential roles in innate and adaptive immunity [134]. Macrophages can be classified into M1 and M2 phenotypes. The proinflammatory M1 phenotype macrophages are primarily responsible for engulfing apoptotic neutrophils and removing pathogens and debris from local tissues. The early inflammatory responses mediated by M1 macrophages are necessary for tissue repair; however, their removal results in delayed healing, while prolonged activation of M1 macrophages may lead to excessive inflammation [135–137]. Distinctive BAPPs modulate the polarization and inhibition of M1 macrophage. Various inflammatory molecules and cytokines can promote M1 polarization. Interferon-γ (IFN-γ) predominantly acts through the JAK1/2-STAT1 pathway to induce M1 polarization, enhancing the expression of other proinflammatory factors such as IL-6 and tumor necrosis factor-α (TNF-α), with TNF-α being a potent promoter of M1 polarization as well as a significant inhibitor of M2 polarization [47]. Granulocyte–macrophage colony-stimulating factor (GM-CSF) promotes the polarization of M1 macrophage mainly through the JAK2-STAT5 pathway [48]. Conversely, progranulin and its derivative peptide atsttrin can bind to the TNF receptors to inhibit the phenotype and function associated with LPS-induced M1 macrophage polarization via the nuclear factor kappa-B (NF-κB) and MAPK signaling pathways [49]. APETx2 is the second peptide targeting acid-sensing ion channel (ASIC), specifically inhibiting both homodimeric ASIC-3 and heterodimeric channels containing ASIC-3 [138]. APETx2 downregulates the expression of ASIC-3 channel proteins, subsequently impeding downstream activation of the NF-κB and p38(MAPK) pathways, which further decrease the secretion of inflammatory cytokines such as IL-1β, IL-6, and TNF-α [50]. Alternatively, MLIF downregulates the expression of IL-1β and IL-8, as well as its metabolites such as a tetrapeptide (Gln-Cys-Asn-Ser), can also inhibit the expression of cytokines including IL-1β, IFN-γ, and IL-6, thereby exerting anti-inflammatory effects [51]. Moreover, human β-defense 1 (HBD1) is a naturally occurring cationic antimicrobial peptide in the human body with essential functions such as broad-spectrum antimicrobial activity, inflammation inhibition, differentiation modulation, anticancer properties, and immune chemotaxis [139, 140]. It has been shown that the HBD family has a high affinity for LPS in vitro and can directly bind to LPS, thus inhibiting inflammatory response [139, 140]. Then, a short HBD1-derived peptide known as Pep-B (ACPIFTKIQGTCYRG) was developed, and it was found that Pep-B reduces the expression of proinflammatory markers induced by LPS such as ROS, and inhibits inflammatory activation mediated by NF-κB, Erk-MAPK, and p38(MAPK) signaling pathways [53]. The mRNA levels of proinflammatory factors including IL-6, TNF-α, IL-8, TLR-2, TLR-4, and other proinflammatory markers decreased in response to PEP-B in the differentiation microenvironment of dental pulp stem cells [52].

When the inflammatory response is prolonged, the anti-inflammatory M2 phenotype is primarily activated. M2 macrophages are capable of secreting IL-10, C chemokine ligand (CCL) 13, TGF-β, PDGF, VEGF, and IGF to promote tissue repair [136, 137, 141]. BAPPs that induce M2 macrophage polarization mainly include cytokines such as IL-4, IL-10, IL-13, and stromal cell-derived factor-1α (SDF-1α) [54], as well as immunomodulatory proteins like CD200 [55]. Among these factors, IL-4 and IL-10 serve as fundamental pro-M2 polarizing factors and act endogenously through the JAK1/2-STAT6 pathway [142, 143]. CD200 is a biomolecule expressed on the surface of neutrophils, macrophages, and dendritic cells that regulates cell activation. It promotes M2 polarization by enhancing the secretion of the anti-inflammatory cytokine IL-10 and also impedes the secretion of M2 polarization inhibitory factors such as TNF-α and IL-6 [55]. However, the effect of M2 macrophages in tissue repair is a double-edged sword, excessive M2 polarization may lead to tissue hyperfibrosis [144]. Therefore, a sequential activation of M1 and M2 macrophages to an appropriate extent is essential for immune modulation during tissue repair [145].

#### Adaptive immune cells

Th1 and Th17 cells secrete proinflammatory cytokines such as IFN-γ, TNF-α, and IL-17, which facilitate M1 macrophage polarization, leading to tissue damage repair [146]. Conversely, Th2 cells release cytokines including IL-4, IL-5, and IL-13that regulate M2 macrophage activity to suppress inflammatory responses mediated by Th1 and Th17 cells [147, 148]. However, overactivation of Th2 cells may lead to allergies and pathological fibrosis due to their direct stimulation of M2 macrophages [144]. The interplay between Th1 and Th2 cells involves mutual regulation; thus, transitioning from a Th1 phenotype to a Th2 phenotype promotes inflammation reduction and tissue regeneration. Vasoactive intestinal peptide (VIP), a neurotransmitter of 28 amino acids found in the central and enteric nervous systems, plays a crucial role in regulating the balance between Th1 and Th2 balance. VIP decreases the secretion of IL-12 and inhibits the expression of transcription factors associated with the differentiation of Th1 cells. Moreover, VIP promoted the expression of C-MAF, GATA-3, and JUN-B, which are key regulators for promoting Th2 cell function It also induces the expression of chemokine CCL2 while suppressing CXCL-10 production [56]. Additionally, MLIF not only influences monocytes but also participates in Th2 polarization by elevating levels of the anti-inflammatory factor IL-10 while concurrently inhibiting the synthesis of proinflammatory factors such as IL-1β and TNFs [57].

Regulatory T (Treg) cells are an essential component of the adaptive immune system, and they maintain immune homeostasis by suppressing autoreactive and hyperactive immune responses [149]. Treg cells are indispensable for the repair of various tissues, capable of being recruited from lymphoid organs to injury sites where inflammation has subsided through a variety of inhibitory mechanisms, including neutrophil regulation [150], Th2 cell regulation [151], and M2 macrophage polarization [152]. Furthermore, Treg cells can directly promote tissue regeneration by activating tissue-specific stem and progenitor cells [153]. Given their pivotal role in tissue repair, understanding how to induce Treg cell activation is an essential topic for the application of immunomodulatory peptides and proteins. Both IL-2 and TGF-β are integral to the induction and regulation of Treg cells. The majority of Treg cells express the CD25 molecule (IL-2R receptor), which exhibits a high affinity for IL-2 and initiates CD25-mediated signaling through activation of the transcription factor STAT5 [154]. Previous studies have demonstrated that Treg cell differentiation requires a “two-step” model wherein robust stimulation via the T-cell antigen receptor (TCR) induces CD25 expression in precursor cells, which is subsequently enhanced by IL-2 stimulation, ultimately leading to *Foxp3* expression [155–157]. Ogawa et al. [158] argued that Foxp3 expression is induced by the transcription factors Smad3, Stat5, and c-Rel, each facilitating distinct signaling pathways. Smad3 is activated by the TGF-β signaling pathway, Stat5 responds to signals from IL-2Rγ, TCR, and TGF-β signaling pathway, while c-Rel activation occurs via TCR signaling pathway [159]. Notably, both Smad3 and Stat5 can be regulated by TGF-β to promote *Foxp3* gene expression.

### Repair cells

Following tissue injury, repair cells such as stem cells or tissue-specific precursor cells are essential for maintaining cell viability and optimal functionality. These cells are subsequently recruited to the injury site, where their adhesive state plays a crucial role in facilitating tissue repair. The ensuing proliferation and differentiation of these repair cells further contribute to the repair process. Throughout these mechanisms, BAPPs exert critical effects on tissue repair by sustaining the survival of repair cells, modulating cellular senescence, and promoting their recruitment, adhesion, proliferation, and differentiation.

#### Cell survival

After being recruited and localized to injury sites, repair cells endure various detrimental conditions, including ischemia, oxidative stress, and inflammatory factors. The precise and rational regulation of these distinct cell death processes is important for tissue repair.

**Fig. 5:**
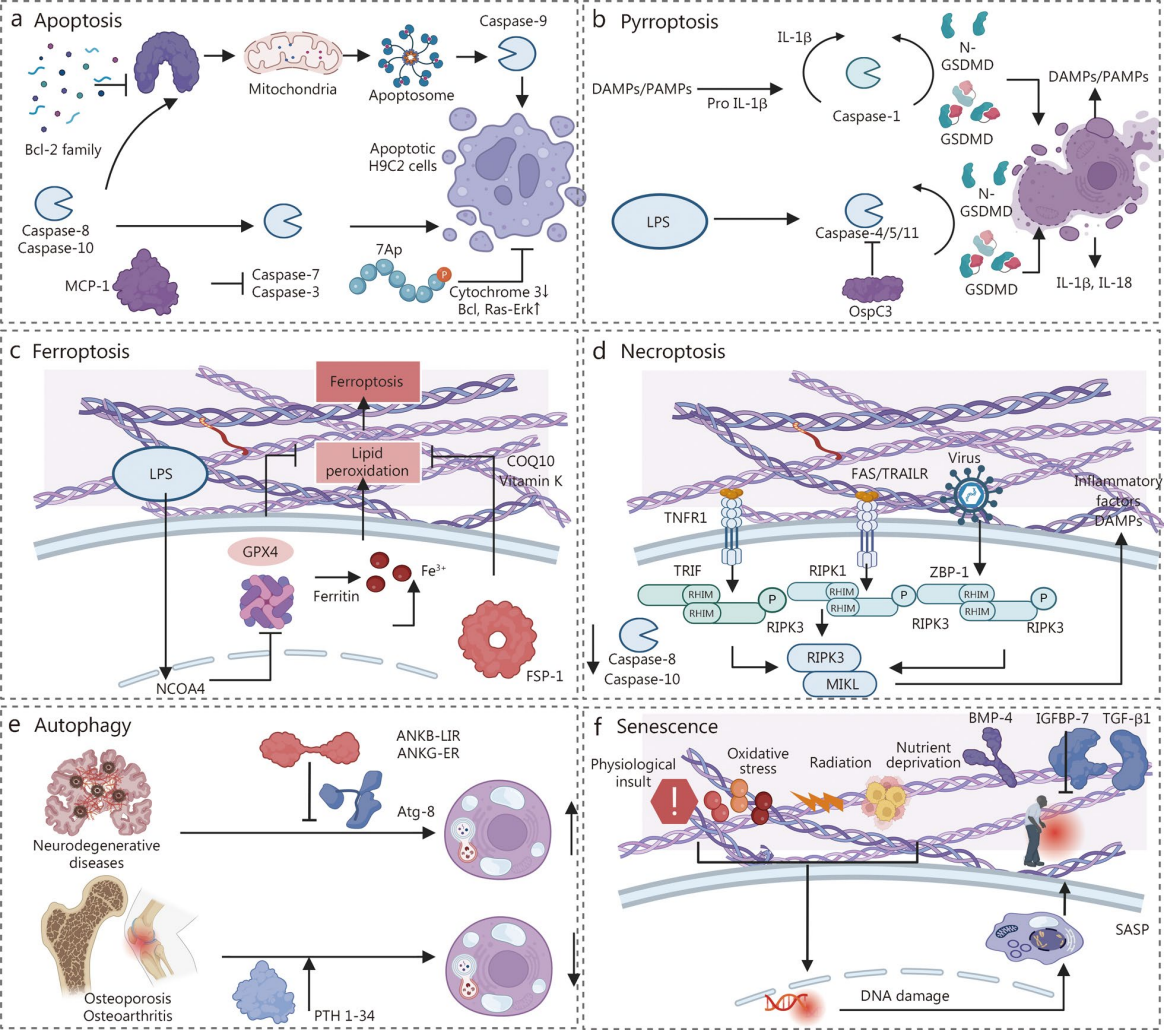
Bioactive peptides and proteins (BAPPs) for cell survival and cell senescence. **a** Bcl-2 family, MCP-1 and 7A inhibit cell apoptosis. **b** Ospc3 inhibits cell pyroptosis through caspase-4/5/11. **c** FSP-1 inhibits lipid peroxidation, thereby inhibiting ferroptosis. **d** Potential pathways to inhibit cell necroptosis. **e** ANKB-LIR, ANKG-ER, and PTH 1-34 modulate autophagy balance in neurodegenerative diseases, osteoporosis, and osteoarthritis. ANKB-LIR ankyrin-B extended LIR motif, ANKG-ER ankyrin-G extended E1991R LIR motif, PTH 1-34 parathyroid hormone-related peptide 1–34. **f** TGF-β1, BMP-4, and IGFBP-7 inhibit cell senescence. BAX BCL2-Associated X, BAK BCL2-Associated K, MCP-1 monocyte chemotactic protein-1, 7Ap phosphorylated HDAC7-derived peptide 7A family, Bcl B-cell lymphoma, Ras-Erk1/2 Ras-extracellular signal-regulated kinases1/2, IL interleukin, DAMPs damage-associated molecular patterns, PAMPs pathogen-associated molecular pattern, LPS lipopolysaccharide, GSDMD gasdermin D, OspC3 Shiga bacterial effectors, GPX glutathione peroxidase, NCOA4 nuclear receptor coactivator 4, FSP-1 ferroptosis suppressor protein-1, TNFR1 tumor necrosis factor receptor1, FAS factor-related Apoptosis, TRAILR tumor necrosis factor-related apoptosis-inducing ligand, RIPK receptor-interacting protein kinase 1, ZBP-1 Z-DNA binding protein 1, MIKL mixed lineage kinase domain-like protein, PTH parathyroid hormone, ANKB-LIR ankyrin-B motif, ANKG-ER ankyrin-K motif, BMP-4 bone morphometric proteins-4, IGFBP-7 IGF binding protein-7, TGF-β transforming growth factor β, SASP senescence-associated secretory phenotype, TRIF Toll/IL-1 receptor (TIR) domain-containing adaptor

Apoptosis was the first identified form of programmed cell death and is thought to play a major role in the development and progression of various diseases such as myocardial infarction. Certain BAPPs exhibit potential for targeting apoptosis to facilitate tissue repair. The anti-apoptotic protein B-cell lymphoma-2 (Bcl-2) inhibits cardiomyocyte apoptosis in infarcted hearts. The BH4 domain is a distinctive structural feature of this class of anti-apoptotic proteins [58]. Monocyte chemotactic protein-1 (MCP-1), also referred to as CCL2 exerts anti-apoptotic effects by decreasing the activity of caspase-3/7 in H9C2 myofibroblasts [59]. Furthermore, members of the histone deacetylase (HDAC) family, particularly the phosphorylated HDAC7-derived peptide 7A family(7Ap), are crucial in promoting chromatin compression and regulating gene transcription, thereby influencing multiple facets of cardiovascular disease, including myocardial infarction [60, 61] (Fig. 5a). Pyroptosis is another type of programmed cell death accompanied by the release of large amounts of proinflammatory factors such as IL-1β and IL-18. The effector protein OspC3 secreted by Shigella has been shown to inhibit LPS-induced cellular pyroptosis [62] (Fig. 5b). Additionally, ferroptosis is an iron-dependent pathway that leads to increased lipid peroxidation, and may significantly contribute to degenerative diseases across various organs while being associated with oxidative damage [160]. Doll et al. [63] reported that ferroptosis suppressor protein 1 (FSP1) can suppress ferroptosis by eliminating lipid peroxidation through the reduction of COQ10 and vitamin K, thus working synergistically with glutathione peroxidase 4 and glutathione to effectively inhibit phospholipid peroxidation and ferroptosis [63] (Fig. 5c). Moreover, necroptosis, a type of programmed cell death characterized by cellular swelling and rupture, is highly proinflammatory and often considered deleterious (Fig. 5d). Therefore, the use of BAPPs to block programmed cell death may be a potential strategy for tissue repair.

Autophagy is essential for maintaining energy homeostasis by regulating the degradation of cellular molecules and organelles. Dysregulation of autophagy has been implicated in the development of various human diseases, including neurodegenerative diseases, cystic fibrosis, cardiomyopathy, osteoarthritis, and osteoporosis [161, 162]. Consequently, modulating autophagy with specific BAPPs holds great potential for tissue repair (Fig. 5e). Li et al. [64] demonstrated that the AnkB-LIR peptide binds to all Atg8 family members with high affinity; thus, this peptide serves as a nonselective inhibitor of autophagy targeting all Atg8 proteins. Furthermore, Li et al. [64] developed a GABARAP-selective AnkG-ER peptide that may selectively inhibit GABARAP-mediated autophagy while minimally interfering with LC3-mediated autophagy. Neurodegenerative lesions are frequently associated with excessive autophagy. Thus, these two peptides could be potential therapeutic agents for neurodegenerative diseases. However, treatments aimed at osteoporosis and osteoarthritis require the promotion of autophagy. Research indicates that parathyroid hormone (PTH) inhibits the senescence of rat nucleus pulposus cells by activating autophagy through the mTOR pathway [65]. Animal experiments have also shown that PTH improves knee osteoarthritis in rats through autophagy [66].

#### Cell senescence

Cellular senescence is a stable and irreversible state of growth arrest caused by physiological insult, oxidative stress, radiation, nutrient deprivation, and other factors, wherein prolonged senescence reduces the regenerative capacity of tissues and leads to tissue degeneration [121]. BAPPs have been shown potential to inhibit the senescence process, indicating promising application in regenerative medicine. TGF-β signal transduction has been implicated in cell senescence and stem cell senescence (Fig. 5f). Specifically, TGF-β promotes senescence by upregulating the expression of p15, p21, and p27 while downregulating several proliferation factors, including c-Myc [67]. Furthermore, TGF-β1 induces Smad2/3 phosphorylation through activation of Smad1/5/9, thereby inhibiting the TGF signaling pathway [68]. In an investigation by Guan et al. [68] BMP-4 was administered to lung fibroblasts, and the results revealed a reduction in the expression level of senescence markers (p16 and p53), corroborated by similar findings in vivo using murine models. Additionally, IGF binding protein-7 (IGFBP-7) can metabolically activate the bioactivity of SIRT1 deacetylase, resulting in decreased transcription of p21 [69]. IGFBP-7 inhibits the senescence process of dental pulp-derived mesenchymal stem cells (MSCs) while promoting osteogenic differentiation [69]. Therefore, utilizing BAPPs to inhibit cellular senescence is advantageous for regulating the tissue microenvironment and improving the efficacy of tissue repair.

#### Cell recruitment

**Fig. 6:**
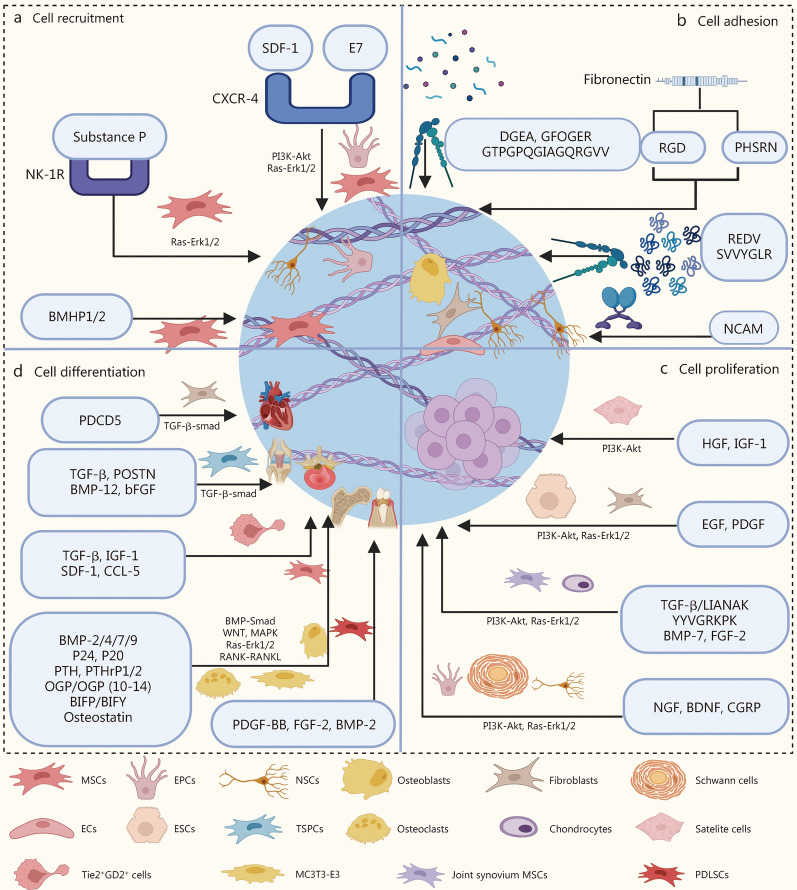
Bioactive peptides and proteins (BAPPs) for repair cells in cell recruitment, cell adhesion, cell proliferation, and cell differentiation. **a** SDF-1, E7, substance P, and BMHP1/2 modulate the recruitment of MSCs, EPSCs, and NSCs through the PI3K-Akt and Ras-Erk1/2 pathways. **b** PHSRN, RGD, NCAM, and other cell adhesion peptides modulate cell adhesion through integrin-αvβ3 α2β1, α4β1, and IgSF. **c** HGF, EGF, TGF-β, NGF, and other BAPPs modulate cell proliferation through the PI3K-Akt and Ras-Erk1/2 pathways. **d** PDCD5, TGF-β, IGF-1, BMP-2, P24, P20, PTH, PTHrP 1/2, OGP, osteostatin, and other BAPPs modulate cell differentiation through the Smad, Ras-Erk1/2, WNT, and MAPK pathways. SDF-1 stromal cell-derived factor-1, E7 EPLQLKM, CXCR-4 chemokine receptor type 4, PI3k-Akt phosphoinositide 3-kinase-protein kinase B, NK-1R neurokinin-1 receptor, DGEA collagen I-derived peptide, RGD adhesion peptide, PHSRN synergy peptide, NCAM neural cell adhesion molecule, PDCD5 programmed cell death 5, TGF-β transforming growth factor-β, POSTN periostin, BMP bone morphogenetic protein, bFGF basic fibroblast growth factor, IGF-1 insulin-like growth factor-1, PTH parathyroid hormone, PTHrP parathormone related peptide, OGP osteogenic growth peptide, BIFP binding-induced fibrillogenesis peptide P, BIFY binding-induced fibrillogenesis peptide Y, MAPK mitogen-activated protein kinases, RANK receptor activator of nuclear factor-kappa B, FGF fibroblast growth factor, HGF hepatocyte growth factor, EGF epidermal growth factor, PDGF platelet-derived growth factor, NGF nerve growth factor, BDNF brain-derived neurotrophic factor, CGRP calcitonin gene-related peptide

Cell recruitment peptides/proteins primarily function by recruiting repair cells that are naturally present in various tissues, including bone marrow MSCs (BMSCs), satellite cells, fibroblasts, and neural stem cells (NSCs), to the site of damage, which is critical for early tissue repair (Fig. 6a). Stromal cell-derived factor-1 (SDF-1) is a small molecule cytokine capable of recruiting these repair cells. Research has demonstrated that SDF-1 promotes the recruitment of monocytes during the early stages of tissue repair via its anti-inflammatory properties. Additionally, SDF-1 inhibits GSK3β activation by binding to the specific receptor CXCR4 and activating the PI3K-Akt pathway, thereby enhancing *β-catenin* gene expression and facilitating the recruitment of MSCs and endothelial progenitor cells to injury sites [70]. The activation of the PI3K-Akt pathway via the SDF-1-CXCR4 complex also plays a vital role in recruiting other stem cell peptides. In addition to SDF-1, several bioactive peptides have been identified as capable of recruiting stem cells. For example, EPLQLKM (E7) is a newly discovered peptide with a specific affinity for endogenous BMSCs [71]. The E7 peptide efficiently recruits BMSCs both in vitro and in vivo, but the mechanism of action remains unclear. The current hypothesis suggests that upon recruiting BMSCs, E7 first activates the binding of SDF-1 to the C-X-C chemokine receptor type 4 (CXCR4) receptor before regulating the proliferation, differentiation, and migration of BMSCs through the p38(MAPK), Ras-Erk1/2, and the PI3K-Akt signaling pathways [72]. Furthermore, bone marrow-homing peptides identified by phage display screening are enriched in the amino acids K, P, F, S, and T, showing high affinity for stem cells. BMHP1 (PFSSTKT) and BMHP2 (SKPPGTSS) have been widely used [73, 74]. In bone tissue and cartilage regeneration, substance P preferentially binds to NK-1Rs and activates the G protein-coupled signaling pathway along with the Ras-Erk1/2 signaling pathway in MSCs, contributing significantly to their recruitment [75].

#### Cell adhesion

Cell adhesion mainly depends on cell adhesion proteins (Fig. 6b). Fibronectin (FN) is an adhesion protein found in the extracellular matrix (ECM) that mediates the adhesion and spreading of various cell types by organizing homologous repeat modules into functional domains [78]. The most widely studied peptide derived from FNs is the tripeptide arginine-glycine-aspartic acid (RGD) [76]. However, it has been argued that RGD alone does not accurately represent the affinity of FN for integrins [163]. This limitation arises because FN possesses a second epitope, proline-histidine-serine-arginine-aspartic acid (PHSRN), which acts synergistically with RGD to enhance the ability of repair cells to adhere, migrate, and spread through activation of the α5β1 receptor [164, 165]. In addition to RGD and PHSRN, several peptides/proteins target specific cells. For example, neural cell adhesion molecule (NCAM) is a membrane-bound glycoprotein that is specifically expressed throughout the nervous system and facilitates adhesion and interconnection between neurons by forming a highly adherent meshwork on the cell surface via a transmembrane structure dependent on numerous fine membrane numbers [79]. Furthermore, DGEA, GFOGER, and GTPGPQGIAGQRGVV mediate the selective adhesion of osteoblasts to integrin α2β1, while REDV and SVVYGLR facilitate selective vascular ECs adherence to integrin α4β1 [80].

#### Cell proliferation

Cell proliferation, primarily mediated by the PI3K-Akt and Ras-Erk1/2 pathways, represents a crucial aspect of tissue repair. These two pathways act as fundamental mechanisms that respond to the binding of BAPPs to their respective receptors, thereby promoting cell proliferation. Cell proliferation-promoting peptides/proteins predominantly serve on various repair cells during tissue repair by activating specific upstream pathways through binding to first the corresponding receptors and then activating two common downstream pathways such as the PI3K-Akt pathway and the Ras-Erk1/2 pathway. The PI3K-Akt pathway regulates cell growth primarily through its influence on the TSC1/TSC2 complex and mammalian target of rapamycin (mTOR) signaling [166]. BAPPs associated with cell proliferation include TGF-β along with its related peptide LIANNK; IGF-1 and its related peptide; hepatocyte growth factor (HGF), fibroblast growth factor (FGF), and epidermal growth factor (EGF), brain-derived neurotrophic factor (BDNF), nerve growth factor (NGF), and calcitonin gene-related peptide (CGRP) [102]. These BAPPs can activate the PI3K-Akt and RAS-ERK1/2 pathways, which subsequently upregulate the expression of transcription factors ETS-1, ATF-1, and ELK-1 to induce cell proliferation as well as cyclin D1 protein to regulate the cell cycle, ultimately promoting the proliferation of stem cells and specific repair cells (Fig. 6c).

#### Cell differentiation

Cell differentiation-promoting peptides/proteins typically interact with their specific receptors to induce the differentiation of repair cells, typically stem cells or precursor cells, into specific tissue cells for tissue repair through unique signaling pathways (Fig. 6d). For example, TGF-βs, bone morphometric proteins (BMPs) and their related peptides, such as P24 and P20, mediate the differentiation of osteoblasts and chondrocyte lineages via both Smad-dependent and Smad-independent signaling pathways, but they recruit distinct receptor heterotetramers and R-Smad complexes, thus exerting different biological effects on osteogenesis, osteoclastogenesis, and chondrogenesis [167]. A strong connection between osteogenic growth peptide (OGP) and OGP (10 − 14) along with its cellular receptor during bone formation can promote the osteogenic-like differentiation of MSCs in multiple ways [93]. Additionally, NGF interacts with two primary receptors, TrkA and p75NTR, and neurorestorative effects are mediated by two receptor-mediated signaling pathways to promote neuronal differentiation and axon growth, repair damaged neurons, maintain the survival of mature neurons, and facilitate the regeneration of axons [110]. BDNF binds to its specific receptor, tropomyosin receptor kinase, to activate the PI3K-Akt, Ras-Erk1/2, and phospholipase C γ1**/**protein kinase C (PLCγ1/PKC) pathways, thus promoting the directional differentiation of NSCs, neuronal migration, as well as further axon and dendrite regeneration [168].

Cell differentiation-promoting peptides/proteins can also exert distinct functions in tissue repair by interacting with receptors on various tissue-specific cells. For example, the BIF peptides BIFP and BIFY bind to RANK and RANKL, effectively blocking the RANK-RANKL interaction, and further attenuating osteoclast activity to promote bone regeneration [94]. The pentapeptide PHTrP107-111 (osteostatin), which includes the Thr-Arg-Ser-Ala-Trp sequence from the C-terminal fragment of PTH-related proteins, promotes osteogenic differentiation of MSCs, inhibits senescence and inflammation in osteoarthritic osteoblasts, reverses skeletal alterations associated with IGF-1 deficiency, and improves bone regeneration in animal models of bone defects [95, 96]. Platelet-derived growth factor-BB (PDGF-BB) is a pleiotropic bioactive protein exhibiting chemotactic, mitogenic, differentiating, and angiogenic properties, and it interacts with its receptors across multiple cells, such as mesangial cells, MSCs, fibroblasts, and smooth muscle cells, for tissue regeneration [169]. Heparin-binding epidermal growth factor-like growth factor (HB-EGF) is another versatile bioactive protein involved in various tissue repair processes such as wound healing, periodontal regeneration, and neurogenesis following ischemic injury by functioning on numerous cell types [170]. FGFs constitute a large family of secretory molecules that interact with tyrosine kinase receptors to direct diverse cell types, including ECs, chondrocytes, neurons, and smooth muscle cells, for specific tissue repair outcomes [171].

Moreover, cell differentiation-promoting peptides/proteins can mediate the directional differentiation of multipotential cells for specific tissue repair by promoting one pathway while inhibiting another. For instance, PTH and PTH-related peptides/proteins bind to PTH1R to enhance the expression of BMP-2, VEGF, and the anti-apoptotic gene *Bcl-2* through the cAMP-PKA pathway, which also phosphorylates the S358 site of salt-inducible kinase 2 (SIK2), thereby inhibiting the activity of SIK2 and subsequently downregulating the MEF2C expression as well as reducing the expression of the sclerotin-encoding gene *SOST*, ultimately leading to upregulation of Wnt4 ligand for osteogenesis [172]. Furthermore, PTH and its derivatives increase SETDB1 expression to inhibit the expression of the peroxisome proliferator-activated receptor gamma gene through the NLK pathway within the MAPK signaling cascade, thereby suppressing the adipogenic differentiation of MSCs [173, 174]. The promotion of osteogenesis alongside inhibition of adipogenesis by MSCs significantly contributes to bone tissue repair, particularly in osteoporotic bone regeneration within a microenvironment where MSCs are predisposed toward adipogenic differentiation rather than osteogenic differentiation.

## Delivery platforms for BAPPs

**Fig. 7:**
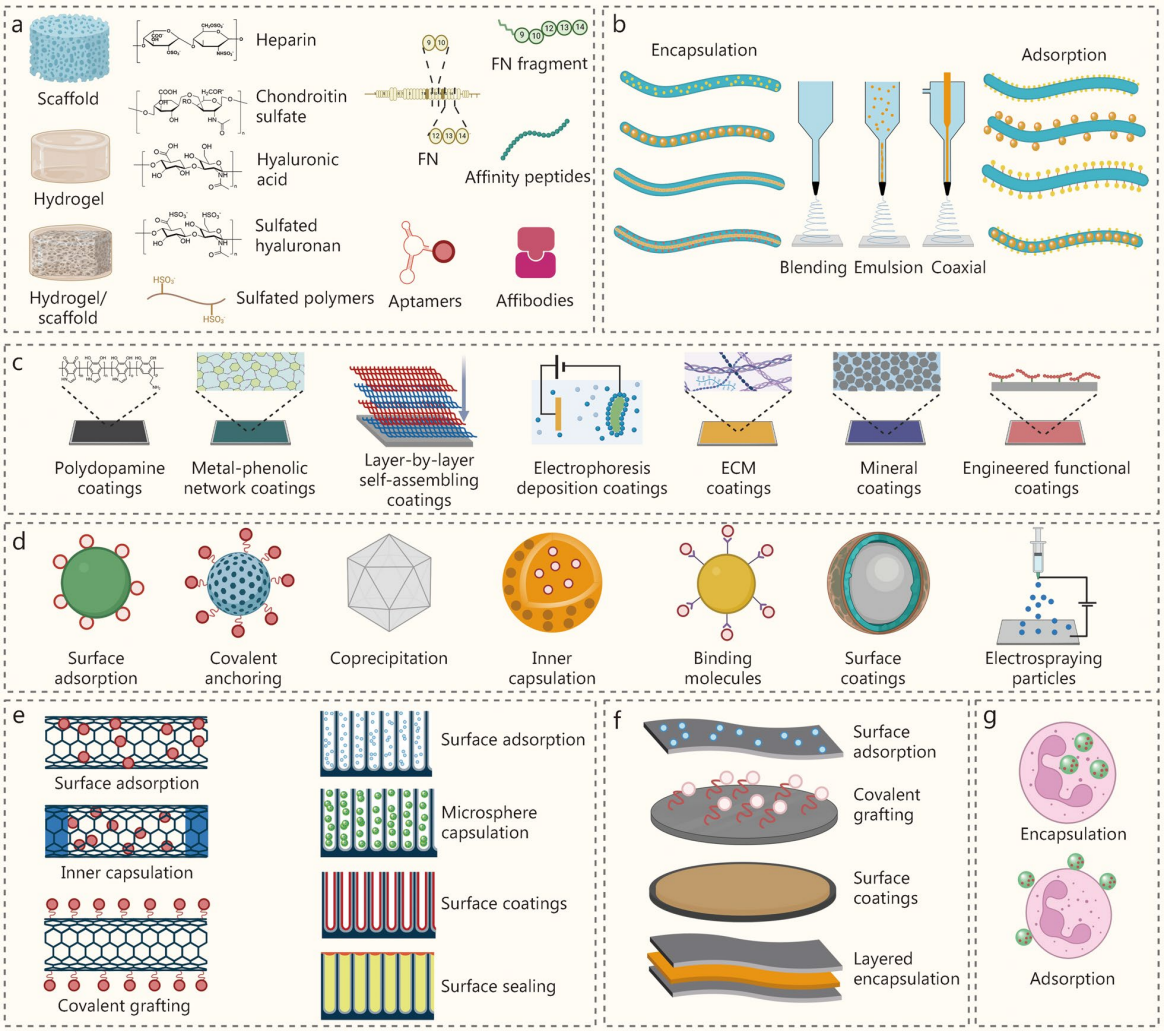
Distinctive delivery platforms coupled with modification strategies for tissue repair. **a** Scaffolds and hydrogels; Strategies to improve affinity for bioactive peptides and proteins (BAPPs), including glycosaminoglycans (GAGs) and sulfated polymers, fibronectin and derivatives, affinity peptides and affibodies, and aptamers. **b** Types of electrospun fibers (blending, coaxial, and emulsion) and delivery strategies by encapsulation and adsorption. **c** Distinctive surface coatings for delivery, including polydopamine coatings, metal-phenolic network coatings, extracellular matrix (ECM) coatings, layer-by-layer self-assembly coatings, electrophoretic deposition coatings, mineral coatings, and engineered functional coatings. **d** Delivery strategies for assisted particles, including surface adsorption, covalent grafting, coprecipitation during synthesis, inner capsulation, binding molecules, surface coatings, and electrospraying. **e** Delivery strategies for nanotubes by surface adsorption, inner capsulation, and covalent grafting; delivery strategies for TiO_2_ nanotubes include surface adsorption, microsphere capsulation, surface coating, and surface sealing. **f** Delivery strategies for two-dimensional nanomaterials, including surface adsorption, covalent grafting, surface coatings, and layered encapsulation. **g** Nanoparticles engineered cells for delivery by encapsulation and adsorption. FN fibronectin

Various delivery platforms have been developed to incorporate BAPPs for tissue repair (Fig. 7), including scaffolds, hydrogels, electrospun fibers, surface coatings, assisted carriers, nanotubes, two-dimensional nanomaterials, and nanoparticles engineered cells. Furthermore, appropriate modifications to these delivery platforms improve the incorporation efficiencies and facilitate fast, controlled, sustained, or heterogeneous release.

### Scaffolds and hydrogels

Scaffolds and hydrogels are commonly used in tissue repair, with BAPPs being directly incorporated into their matrix [175, 176]. These materials not only provide structural support and facilitate cell adhesion and growth but also serve as an interconnected porous network that regulates the release of BAPPs. Additionally, hydrogels can be employed as coatings to modify porous scaffolds, thus increasing the loading capacity and retention of BAPPs [177]. Although controlled release of BAPPs can be achieved by steric hindrance within the scaffold or hydrogel matrix, the release kinetics may be optimized utilizing various binding systems, including glycosaminoglycans (GAGs), binding proteins and peptides, as well as aptamers.

#### GAGs and sulfated polymers

GAGs are linear ECM polysaccharides composed of amino sugars and aldehydes, including heparin, heparan sulfate, chondroitin sulfate, and HA. GAGs exhibit potent affinity for BAPPs via nonspecific or specific interactions. Specifically, GAGs possess numerous sulfate and carboxylic acid groups that confer highly electronegative properties, facilitating the nonspecific adsorption of positively charged BAPPs. Furthermore, BAPPs can specifically bind to designated binding domains in GAG molecules via hydrogen bonds, van der Waals forces, and hydrophobic interactions [178].

Heparin, heparan sulfate, and chondroitin sulfate are rich in sulfate and carboxylic acid groups, enabling them to bind positively charged BAPPs. These compounds have been used to regulate the release of BAPPs for tissue engineering and regeneration, incorporated into scaffolds or hydrogels achieved through physical interactions or chemical grafting. Conversely, the negatively charged properties of GAGs facilitate their introduction to scaffolds or hydrogels containing cationic components via electrostatic interactions. For example, Ding et al. [179] simultaneously coupled monocarboxylic acid-terminated polyethylene glycol (PEG) with arginine to polyethylene aspartic acid diglyceride, thus developing a biodegradable polycationic polymer. Researchers then combined negatively charged heparin with a positively charged polymer to fabricate a hydrogel by electrostatic interactions, achieving a stable release of FGF-2 for up to 16 d [179]. In another study, researchers integrated heparin and chitosan to develop hydrogels for BMP-2 delivery aimed at osteogenesis [180]. To enhance the local anchoring of GAGs, binding peptides exhibiting high affinity for GAGs can be introduced to scaffolds or hydrogels. For instance, heparin-binding peptides were incorporated into self-assembling peptides that interact with heparan sulfate to control the release of bioactive proteins [181]. On the contrary, GAGs can be covalently coupled to scaffolds or hydrogels due to their abundant functional groups, including amine groups, carboxyl groups, and sulfhydryl groups. Heparin was first thiolated before covalently introduced to chitosan-based hydrogels containing poly(ethylene glycol-B-caprolactone-B-ethylene glycol) via Michael’s addition reaction [182]. Thiolated heparin exhibits a high affinity for basic FGF (bFGF), allowing its content adjustment to modulate bFGF release [182]. Moreover, the carboxyl groups of heparin can undergo amide coupling reactions with other amine-containing groups, thereby being incorporated into the hydrogel. 1-(3-dimethylaminopropyl)-3-ethylcarbodiimide (EDC)/N-hydroxy succinimide (NHS) chemistry has been employed to couple these carboxyl groups from heparin with star-shaped PEG end-functionalized amine groups to fabricate hydrogels designed for delivering BMP-2 and VEGF [183].

HA is a linear, non-sulfonated glycosaminoglycan composed of alternating β-1,4-D-glucuronic acid and β-1,3-N-acetyl-D-glucosamine [184]. The presence of carboxyl groups in the glucuronic acid sugar units enables HA to bind and interact with BAPPs for sustained release. For example, Deng et al. [185] incorporated HA into a PDLLA-PEG hydrogel to regulate the release of TGF-β3. Their results indicated that the addition of HA reduced the burst release of TGF-β3 while prolonging its release over 21 d. To enhance the binding properties of HA, sulfate groups can be utilized for modification. Specifically, researchers synthesized sulfated HA (sHA) and further constructed sHA/collagen hydrogels for delivering HB-EGF in skin wound repair, and the findings demonstrated that sHA had an enhanced binding ability for HB-EGF without any burst release occurring [186].

Given that the sulfate group is the key heparin mimetic moiety contributing to binding affinity, various polymers generally employed in the construction of scaffolds or hydrogels, such as alginate, chitosan, dextran, and cellulose, can be sulfated to enhance noncovalent interactions with BAPPs. For example, sulfated alginate can be synthesized through the esterification of hydroxyl groups; however, this process reduces the viscosity of alginate, necessitating additional materials for scaffolds or hydrogel formation [187]. Wang et al. [188] synthesized sulfated alginate and combined it with gelatin methacryloyl (GelMA) to fabricate bio-inks featuring an interpenetrating network. The resulting sulfated alginate was capable of adsorbing TGF-β3 via electrostatic interactions to promote cartilage formation. These findings showed that the release of TGF-β3 from the sulfated bio-inks reached (42.2 ± 5.8)% on day 7 and continued throughout 21 d [188]. Additionally, sulfated alginate has been added to bind specific BAPPs, such as FGF-2, HGF, IGF-1, and TGF-β1. Furthermore, other polymers, including cellulose, glucan, and chitosan, can be sulfated to optimize the delivery of BAPPs for tissue regeneration.

#### FN and its derivatives

FN is a glycoprotein abundantly present in the ECM that consists of three types of subunits (FN I-III). The FN III subunit features a binding domain known as FN III_12-14_, which contains positively charged amino acids, lysine, and arginine, facilitating interactions with multiple bioactive proteins, including TGF-β, BMP-2, BMP-6, BMP-7, PDGF-BB, and HGF. The binding domain adjacent to the integrin binding site (FN III _9–10_) in FN molecules can trigger cooperative signaling between integrins and protein receptors to amplify the effects of bioactive proteins for tissue regeneration. Therefore, fibronectin is a potent molecule for the delivery of BAPPs to scaffolds and hydrogels.

FNs can be incorporated into scaffolds or hydrogels through physical adsorption or chemical coupling. Typically, FN physically adsorbs on polymers in a globular conformation, but ethyl polyacrylate (PEA) can promote the assembly of FN into a network on its surface, thus exposing the integrin binding region (FN III_9-10_) and the growth factor binding region (FN III_12–14_) to repair cells [189]. Taking advantage of this property, Cheng et al. [190] developed a polyPEA-coated three-dimensional (3D) polycaprolactone (PCL) scaffold and then physically immobilized FN on its surface to fabricate a nanocoating for delivering BMP-2 via adsorption. The results revealed that 90% of the loaded BMP-2 remained adsorbed on the coating after 14 d, greatly reducing the treatment dosage of BMP-2 [190]. Furthermore, FN can be covalently conjugated to scaffolds or hydrogels due to disulfide bonds present in the FN I and FN II domains, while the FN III domain lacks such bonds. Consequently, these disulfide bonds enable the covalent coupling of FN molecules to substrates without affecting the functional site on FN III. For instance, researchers covalently conjugated FN to 4-arm PEG using a Michael-type addition reaction to fabricate hydrogels for delivering VEGF and BMP-2, and the findings demonstrated sustained release of BMP-2 and VEGF for osteogenesis and angiogenesis [191].

The utilization of short recombinant FN fragments as substitutes for full-length FNs is more conducive to clinical translation due to their lower production costs. Martino et al. [192] engineered the FN III_9-10_ and FN III_12-14_ fragments through genetic manipulation, resulting in the development of a recombinant FN fragment, FN III_9-10/12–14_, which was subsequently covalently conjugated to the fibrin scaffold. This functionalized scaffold was used to deliver distinct bioactive proteins, including VEGF, PDGF, and BMP, thereby promoting cooperative signaling between GF receptors and integrins simultaneously [192].

The development of FN mimetics is another ideal strategy for substituting FN in binding BAPPs, owing to their low cost and ease of synthesis. Zhang et al. [193] synthesized amphiphilic amino acid copolymers that are positively charged, designed to mimic the FN III_12-14_ domain for binding bioactive proteins. These copolymers were formulated as a mixture of a cationic residue [lysine (Lys)] and hydrophobic residues [norleucine (Nle), norvalin, aminobutyric acid, or alanine], followed by covalent conjugation to a gelatin sponge scaffold for the delivery of bioactive proteins. The results showed that the copolymer Lys0.4-Nle0.6 exhibited the strongest binding affinity for bioactive protein when the ratio of Lys to Nle was 2:3 [193].

#### Affibodies and affinity peptides

Affibodies and affinity peptides are peptide-based molecules developed and screened through display techniques such as yeast surface display and phage display, resulting in their relatively high affinity for specific BAPPs. These affibodies and affinity peptides can be incorporated into scaffolds or hydrogels to bind BAPPs and regulate their release profiles.

Affibodies are a class of small antibody-mimetic proteins characterized by three α-helical structures, derived from one of the immunoglobulin binding domains of staphylococcal protein A [194]. The binding affinity can be readily adjusted by modifying the surface-exposed amino acid sequences on two of three helices, thus enabling the fabrication of highly diverse protein libraries for screening via display techniques. Distinct affinities have been developed and screened to optimize the release of BAPPs. For example, Bostock et al. [194] identified an affibody exhibiting moderate affinity with high specificity for FGF-2 through yeast surface display, subsequently covalently coupling this affibody to an HA hydrogel to control and prolong FGF-2 release. In another study, affibodies with moderate affinity for BMP-2 were generated using yeast surface display and conjugated to PEG-maleimide hydrogels to modulate BMP-2 release [195]. Furthermore, distinct affibodies can independently regulate the release of multiple BAPPs due to their high specificity. For instance, two types of affibodies targeting IGF-1 and PEDF were developed and coupled to hydrogels, and their release rates could be adjusted by changing the strength of interaction between the affibodies and their respective target proteins [196].

Affinity peptides can be screened from peptide libraries using display techniques to selectively bind specific BAPPs. For example, neurotrophin-3 (NT-3)-binding peptides were identified from a Ph.D.-7 peptide library (New England Biolabs) through the phage display technique and subsequently covalently conjugated to PEG hydrogels [197]. The results unveiled that the hydrogel modified with NT-3-binding peptides significantly reduced the burst release while prolonging the release of NT-3 [197].

#### Aptamers

Aptamers, which are single-stranded oligonucleotides consisting of 20 − 60 nucleotides, are selected from synthetic RNA/DNA libraries via the systematic evolution of ligands by exponential enrichment and can bind specifically to target molecules with high affinity. These aptamers can be incorporated into scaffolds or hydrogels to regulate the release kinetics of BAPPs. For example, Zhao et al. [198] introduced anti-VEGF aptamers and anti-PDGF-BB aptamers to fibrinogen hydrogels to bind VEGF and PDGF-BB, and the results suggested that the functionalized hydrogel could simultaneously chelate VEGF and PDGF-BB while delaying their release in a sustained manner. Furthermore, complementary series of aptamers exhibit greater affinity for other aptamers than for BAPPs, allowing these complementary series to modulate the release of BAPPs [199]. Rana et al. [200] loaded VEGF into a GleMA hydrogel containing anti-VEGF aptamers and found that the addition of a complementary series accelerated VEGF release. Moreover, the molar ratio between the complementary series and aptamers could be adjusted to fine-tune the dose that triggered VEGF release.

### Electrospun fibers

Electrospun fibers are promising delivery platforms characterized by their facile preparation and large surface area. BAPPs can be loaded into electrospun fibers using two strategies. The first strategy involves the direct addition of BAPPs to the polymer solution before electrospinning, thereby encapsulating them within electrospun fibers. The second strategy entails adsorbing BAPPs onto the surface of pre-prepared electrospun fibers.

#### Electrospun fibers for encapsulation

BAPPs are directly encapsulated within electrospun fibers, utilizing three electrospinning techniques: blend electrospinning, emulsion electrospinning, and coaxial electrospinning, for their delivery.

Blend electrospinning is the most basic strategy for delivering bioactive peptides or proteins [201]. In this approach, bioactive peptides or proteins were encapsulated in the raw materials to form a homogeneous electrospinning solution, which was then utilized to fabricate electrospun fibers. A study dissolved a PTH-related peptide in cellulose acetate and employed the solution to produce electrospun fibers for wound healing [202]. Another investigation incorporated glial cell line-derived neurotrophic factor in poly(lactic-co-glycolic acid) (PLGA) and NGF into poly(D, L-lactic acid), which were subsequently used to obtain electrospun fibers aimed at nerve tissue repair [203]. However, due to their high solubility, bioactive peptides or proteins may be rapidly released from electrospun fibers. Therefore, it is necessary to introduce binding components into these fibers to prolong the release of bioactive peptides or proteins for long-term tissue regeneration. Additionally, the delayed release could also be achieved through assisted carriers. For instance, Song et al. [204] integrated both nanohydroxyapatite and chitosan-based nanoparticles containing Nel-like molecule-1 (NELL-1) proteins into polycaprolactone (PCL) solutions, which were then used to construct electrospun fibers for bone regeneration. The results indicated that NELL-1 could be slowly released from the fibers in vitro over 30 d [204]. Nevertheless, BAPPs are directly exposed to electrospinning solutions, where organic solvents may lead to reduced activity or loss of biological function.

Emulsion electrospinning is an advanced strategy for delivering bioactive peptides or proteins in the core of electrospun fibers, ensuring a non-random distribution. Given that BAPPs are generally hydrophilic, water-in-oil (W/O) emulsion electrospinning has been adopted for their delivery. The bioactive peptides or proteins were incorporated into the aqueous phase, which was then dispersed into the lipophilic phase to obtain electrospinning solutions. As organic solvents in the lipophilic phase volatilize, the aqueous phase is compressed to the center of the jet, resulting in electrospun fibers with core–shell structures that delay the release of bioactive peptides or proteins. For example, a dextran solution containing bFGF was used as the aqueous phase, while PLGA solution containing wool keratin constituted the lipophilic phase. Following thorough mixing and stirring, these electrospinning solutions underwent emulsion electrospinning to incorporate bFGF into the core of the PLGA/wool keratin composite fibers for bone regeneration, and the sustained release of bFGF was maintained over 28 d [205]. Furthermore, core–shell electrospun fibers produced via emulsion electrospinning can facilitate the co-delivery of multiple bioactive peptides or proteins. Xia et al. [206] fabricated core–shell electrospun fiber scaffolds through this technique for peripheral nerve regeneration. VEGF was incorporated into the shell to promote angiogenesis and the invasion of Schwann cells during the initial days post-release, while NGF was encapsulated in the fiber cores for neuronal differentiation over 1 month. In another study, two-layer vascular grafts were created using emulsion electrospinning with VEGF in the core to enhance endothelialization and bFGF and SDF-1α in the shell to stabilize the effects of VEGF within vascular grafts [207]. However, it is difficult to obtain stable and uniform core–shell structures via emulsion electrospinning due to the generally low surface tension exhibited by these solutions. Additionally, uncontrolled phase separation during processing may lead to core–shell structures.

Compared with blend electrospinning and emulsion electrospinning, coaxial electrospinning is a superior strategy for the delivery of BAPPs. In this method, two different solutions are artificially separated into two needles, while sharing a common spinneret. A key advantage of coaxial electrospinning is that one of the solutions can be non-spinnable, thereby expanding the applications of electrospinning [21]. To facilitate the delivery of BAPPs, core solutions can be introduced during electrospinning to mitigate initial burst release. For example, Evrova et al. [208] incorporated PDGF-BB into the core phase of coaxial electrospun fibers aimed at tendon rupture healing and reported that these fibers achieved sustained release of PDGF-BB over 30 d in vitro and dramatically improved the tensile strength of treated tendons when used as implants. Furthermore, coaxial electrospinning allows for the synthetic delivery of multiple bioactive peptides or proteins according to different regenerative requirements through core–shell electrospun fibers similar to those produced by emulsion electrospinning. A study conducted by Man et al. [209] used polyvinyl pyrrolidone containing TGF-β1 as the core phase and poly(ε-caprolactone) as the shell phase. After coaxial electrospinning, a BMSC-affinity peptide E7 was covalently coupled to the fiber shell to promote cell adhesion. This interaction necessitates relatively long-term effects rather than rapid release [209]. However, if vascularization or immune modulation is considered in the early stages of tissue regeneration, delivering bioactive peptides or proteins to the shell of fibers occurs more rapidly. For instance, BMP-2 was introduced into a poly(L-lactic acid) (PLLA) core solution, while bFGF was loaded into a PLGA shell solution [210]. These components were then used to fabricate a core/shell fibrous scaffold via coaxial electrospinning, and bFGF was integrated into the fiber shells with approximately 70% released during the first few days to promote vascularization and M2 macrophage polarization. In contrast, BMP-2 was incorporated within fiber cores where only about 4% was released simultaneously but continued slow release occurred over 30 d for bone regeneration [210].

The critical point in designing electrospun fibers with core–shell structures is ensuring that the bioactive agents in the inner core are released gradually. Controlling the properties of the shell serves as a strategy for optimizing the release of BAPPs from the core phage. For example, Chen et al. [211] incorporated BMP-2 in polyvinyl alcohol (PVA) as the core material of an electrospun fiber, utilizing silk fibroin and PCL as the outer shell, regulating BMP-2 release by adjusting the ratio of silk fibroin to PCL in the shell. Additionally, the release of growth factors from the core phase of fibers can be optimized through other delivery platforms. The study by Liu et al. [212] embedded TGF-β1 into silk fibroin nanoparticles as the core phase, while VEGF was directly dissolved in a silk fibroin aqueous solution. These two components underwent coaxial electrospinning to produce electrospun fibers with a core–shell structure, which was inserted into decellularized small intestinal submucosa mesh for abdominal wall defect repair [212]. At day 7, the release of VEGF accounted for approximately 80% of its total amount, while TGF-β1 release was around 34% [212]. The rapid release of VEGF coupled with slow TGF-β1 release facilitates mature vascular network formation, and the late-stage slow release of TGF-β1 also contributes to the maturation and remodeling of the extracellular matrix [212].

#### Electrospun fibers for adsorption

While direct encapsulation of bioactive peptides or proteins within electrospun fibers can facilitate their sustained release, this approach typically exposes them to high voltage, which may affect their biological activity. An alternative strategy involves reconstructing electrospun fibers and subsequently immersing them in a solution containing bioactive peptides or proteins for adsorption-based delivery. The efficacy of this method lies in the interactions between the electrospun fibers and the bioactive peptides or proteins. Therefore, it is essential to appropriately modify the electrospun fibers to enhance their capacity for adsorbing these biomolecules.

Modifying electrospun fibers with natural sulfated GAGs such as heparin is a potential strategy for enhancing the interactions between these fibers and bioactive peptides or proteins for sustained release. Lee et al. [213] covalently conjugated heparin to electrospun PCL/gelatin scaffolds, which were employed to immobilize PDGF-BB. The results showed that PDGF-BB could be released gradually from the heparin-coupled scaffolds over 20 d without burst release due to negatively charged sulfonic groups present in heparins. In contrast, non-heparinized scaffolds exhibited a burst release within 5 d. Additionally, one study utilized heparin to modify α, β-poly(N-2-hydroxyethyl)(2-aminoethylcarbamate)-D, L-aspartamide-graft-polylactic acid [PHEA-EDA-g-polylactic acid (PLA)]/PCL scaffolds for retaining bFGF, which were subsequently applied as vascular grafts [214]. In another investigation, PLA/hydroxyapatite fiber scaffolds were fabricated via electrospinning and subsequently modified with heparin. These heparin-modified scaffolds were then used to immobilize VEGF, promoting bone regeneration [215].

Inspired by sulfated GAGs, other molecules containing sulfonic groups could also be modified into electrospun fibers to facilitate the sustained release of bioactive peptides or proteins. For instance, one study initially fabricated sulfated carboxymethylcellulos and then employed them to modify electrospun poly(hydroxybutyrate)/gelatin fibers [216]. The modified electrospun fibers were then utilized to immobilize TGF-β1 through electrostatic interactions, enabling the gradual release of the bioactive protein for at least 4 weeks [216]. Another investigation synthesized 2-N,6-O-sulfated chitosan, which was used to modify electrospun PCL scaffolds for the sustained release of BMP-2 aimed at promoting bone regeneration [217]. Furthermore, modifying raw materials before electrospinning represents an alternative strategy for introducing molecules with sulfonic groups. Mohammadi et al. [218] first sulfated alginate using chlorosulfonic acid (ClSO_3_H) and combined it with PVA as raw materials to produce electrospun fibers intended for the sustained release of TGF-β1. In contrast, modifications applied after the electrospinning process may be more effective in achieving sustained release of bioactive peptides or proteins compared to pre-electrospinning modifications. It is possibly due to a greater exposure of sulfonic groups on the surface of the electrospun fibers facilitating the immobilization of active peptides or proteins.

Electrospun fibers designed for the adsorption of bioactive peptides or proteins have been established through sulfonic groups, but these binding systems predominantly target cationic BAPPs. Consequently, there is potential to develop electrospun fibers utilizing alternative binding systems to facilitate the delivery of bioactive peptides or proteins for tissue repair. For example, graphene oxide (GO) has been incorporated into electrospun fibers to adsorb IGF-1 and BDNF for spinal cord repair, with sustained release profiles of IGF-1 and BDNF confirmed in a model [219]. Further exploration of other binding systems is necessary to effectively deliver distinct BAPPs for tissue regeneration.

### Surface coatings

Surface coatings are used to modify the surfaces of solid substrates, including porous scaffolds, orthopedic implants, and solid carriers. The biophysical or chemical specificity of these surfaces can be improved, thereby enhancing the affinity of BAPPs for delivery platforms. Various surface coatings have been developed to deliver BAPPs for tissue repair.

#### Polydopamine (PDA) coatings

Dopamine undergoes polymerization to form PDA under alkaline conditions, and PDA serves as a biological coating due to its exceptional biocompatibility, adhesive properties, and abundant functional groups, such as carboxyl, amino, and imine groups, that facilitate adsorption onto distinct substrates. Typically, PDA coatings are performed on the surfaces of substrates for the subsequent adsorption of BAPPs. For example, Yang et al. [220] utilized PDA coatings to modify diverse substrates, which were then employed to immobilize adhesion peptides and neurotrophic growth factors. The results suggested that bioactive peptide- or protein-immobilized substrates dramatically promote the proliferation and differentiation of NSCs [220]. In another study, Huang et al. [221] initially fabricated a PDA coating on the surface of a calcium phosphate cement scaffold before covalently grafting chondroitin sulfate onto the PDA layer using adipic acid dihydrazide to bind BMP-2 for bone regeneration. Both the unmodified PDA coating and chondroitin sulfate-functionalized version demonstrated sustained release of BMP-2, particularly during the initial days, but incorporating chondroitin sulfate improved the efficacy of bone regeneration [221]. Additionally, BAPPs can be added to the dopamine precursor solution, and subsequently loaded within PDA coatings through polymerization processes. Godoy-Gallardo et al. [222] developed a dual-factor release system utilizing multilayered PDA coatings. The PCL/HA-based scaffolds were first modified with a PDA coating containing BMP-2, followed by a dithiol connecting layer enabling the deposition of an additional VEGF-containing PDA coat. This design facilitated the rapid initial release of VEGF to induce vascularization while ensuring sustained release of BMP-2 contributed to bone repair [222]. However, it remains unclear whether this approach influences the biological activity of peptides and proteins.

#### Metal-phenolic network (MPN) coatings.

MPN coatings are formed through the coordination of phenolic ligands and metal ions, resulting in organic–inorganic hybrid networks on various substrates characterized by facile synthesis and excellent biocompatibility. Owing to their permeable structure with gating properties, MPN coatings can effectively modulate the release of BAPPs from modified substrates. Zhang et al. [223] developed an MSN coating using copper ions (Cu^2+^) and tannic acid on the surface of porous PLA scaffolds, and this coating acted as a physical barrier that significantly delayed the diffusion and release of BMP-2. The results from the in vitro release experiment showed that the model drug molecule, rhodamine B, exhibited a burst release phase in the initial 60 h and then showed a slow and stable release from 60 to 500 h. Given that BMP-2 has a higher molecular weight than rhodamine B, its release rates at various stages were slower compared to those observed for rhodamine B [223].

#### Layer-by-layer (LBL) self-assembling coatings

LBL self-assembling coatings are fabricated by the alternating deposition of two oppositely charged polyelectrolyte materials, which form coatings for the delivery of BAPPs by electrostatic interactions. On one hand, adsorbing BAPPs to pre-prepared LBL self-assembling coatings is a potential strategy. Typically, polyelectrolytes with a strong affinity for BAPPs, such as heparin and HA, are selected for this purpose. Lu et al. [224] used chitosan as a polycation and heparin as a polyanion to fabricate LBL self-assembling coatings, which were then cross-linked by an EDC/EHS system. These coatings were subsequently utilized to adsorb FGF-2, with results revealing that FGF-2 was released slowly over more than 7 d [224]. In another study, poly(L-lysine) (PLL) and HA were sequentially deposited on a solid scaffold after several cycles [225]. Following stabilization via the EDC/NHS system, LBL self-assembling coatings were employed to adsorb platelet lysates containing multiple bioactive proteins, results demonstrated sustained release of these proteins over 2 weeks [225]. PLL/HA-based LBL self-assembling coatings have also been applied to modify Ti implants before loading them with PTH-related proteins [226]. On the other hand, incorporating BAPPs within multilayer LBL self-assembling coatings presents another viable strategy for the delivery of bioactive peptides or proteins. One study constructed LBL self-assembling coatings using TGF-β2-containing alginate and gelatin solutions to modify the surface of autologous hair follicular stem cells, the findings unveiled that these coatings facilitated sustained release of TGF-β2 for hair regeneration [227]. Another investigation involved the sequential deposition of β-cyclodextrin-grafted chitosan solution containing calcitriol alongside gelatin and calcitonin solutions, to fabricate LBL self-assembling coatings on the surface of Ti-based implants, thus incorporating calcitriol and calcitonin within the multilayers [228]. The results indicated that these composite systems enabled sustained release of calcitonin while promoting the osteogenic differentiation of osteoblasts and enhancing osseointegration in vivo [228]. Therefore, BAPPs can either be adsorbed to the surfaces of pre-prepared LBL self-assembling coatings or loaded into their interior during polyelectrolyte deposition.

#### Electrophoretic deposition (EPD) coatings

EPD coatings are produced by the deposition of charged particles onto the surfaces of substrates immersed in an electrolyte solution under the influence of an electric field. Parameters such as the electric field strength, solution concentration, and deposition time can be precisely controlled to achieve accurate regulation of the thickness, composition, and structure of EPD coatings. Therefore, EPD coatings can effectively deliver BAPPs for tissue repair. For example, Wei et al. [229] used EPD technology to sequentially deposit mesoporous silica nanoparticles (MSNs) and carbon nanotubes (CNTs) on the surface of porous titanium (Ti) scaffolds. The outer MSN layer provides immobilization sites for chemically conjugating MGF, a variant subtype of IGF-1, via EDC/NHS chemistry. The inner CNT layer serves as a buffer that mitigates strain between MSNs and Ti scaffolds due to its superior mechanical properties. The adsorption of MGF in the external pores of MSNs resulted in an initial burst release followed by a cumulative release of 20% on day 3, 50% on day 12, and 70% after 30 d [229].

#### ECM coatings

ECM coatings are fabricated through the adsorption of ECM components, such as heparin, chondroitin sulfate, fibrin, and collagen onto the substrate surface followed by subsequent immobilization. These ECM coatings not only improve the biocompatibility and hydrophilicity of substrates but also exhibit high affinity for BAPPs. For example, Wang et al. [230] immersed a QHM polyurethane scaffold (Q: Quadrol; H: Hexamethylene diisocyanate; M: Methacrylic anhydride) in fibrinogen and subsequently employed thrombin to create a fibrin coating for adsorbing GDF-7 aimed at tendon repair. Results showed that this coating facilitated sustained release of GDF-7 for at least 1 week in vitro. In a separate study, Seong et al. [231] initially adsorbed collagen onto biphasic calcium phosphate microspheres, which were then cross-linked using EDC/NHS chemistry to form a collagen coating for the adsorption of BMP-2 for bone regeneration. The findings indicated that the collagen coating improved the loading capacity while reducing the initial release of BMP-2 from 85 to 55% [231]. Moreover, ECM components can be modified to impact specific properties. For example, thiolated gelatins were synthesized through the reaction of Traut’s reagent with gelatins, which were subsequently utilized to coat nanoceria containing gabapentin for dry eye therapy [232]. The results demonstrated that thiolated gelatins improved cellular uptake of nanoceria and binding efficacy to mucin while also enhancing bioactivity and facilitating the slow release of gabapentin to restore the tear film.

#### Mineral coatings

The development of mineral coatings is a straightforward and effective approach for the sustained release of BAPPs, which can preserve their biological activity over extended periods [233, 234]. Since mineral coatings are formed through incubation deposition in aqueous solutions, they can be applied to the surfaces of materials with complex porous geometries [235]. The binding of BAPPs to the mineral coating relies on electrostatic interactions, while the dissolution of the mineral coating facilitates the gradual release of BAPPs. Therefore, controlled release of BAPPs can be achieved to a certain extent by changing intrinsic characteristics such as thickness dissolution and kinetics of the mineral coating [235]. The surface of the β-tricalcium phosphate (β-TCP) particles was coated with hydroxyapatite by Gonzalez et al. [233] to modify their dissolution rate. The coating served as a controlled release vector for VEGF, modular VEGF peptide, and modular BMP-2 [233]. The nanostructure of the mineral coating protects the bioactivity of growth factors, while an increase in carbonate content accelerates its degradation rate. Then, Yu et al. [235] accomplished a two-factor sequential release using a hydroxyapatite coating. BMP-2 was initially bound to one mineral coating, followed by VEGF binding to another [235]. VEGF exhibited a two-stage release, rapid release during the first 14 d followed by continuous release over the next 36 d. BMP-2 demonstrated continuous release of BMP-2 for 50 d. Notably, both growth factors releases could be regulated by changing intrinsic properties of the mineral coating [235]. Additionally, this calcium phosphate-based mineral coating has been used for controlled bFGF release, enhancing its bioactivity and exhibiting certain heat resistance properties [234].

#### Engineered functional coatings

In addition to delivering BAPPs via surface coatings, BAPPs can be directly covalently attached to the surfaces of solid substrates to create engineered functional coatings. For example, one study covalently linked a transacting activator of transcription (TAT) cell-penetrating peptide to the surface of PCL nanoparticles, enhancing retinal permeability and thereby improving the therapeutic efficacy of dual cargos, including metformin and resveratrol, for treating macular degeneration [236]. Another investigation conjugated the prestin-targeting peptide LS19 onto the surfaces of liposome nanoparticles, facilitating the targeted delivery of forskolin to outer hair cells for addressing noise-induced hearing loss [237]. Unlike small molecular BAPPs, poly(peptides) not only impart specific functions to substrates but also enable sustained release of bioactive drugs. A study by Yang et al. [238] fabricated poly(l-histidine) coatings on ceria nanocages using an EDC chemistry procedure, and they revealed that these coatings could enhance corneal penetration while allowing pH-responsive release of bioactive cargos, including acetylcholine chloride and SB431542, for the treatment of chemical eye injury.

### Assisted particles

Assisted particles serve as prevalent delivery platforms, with sizes ranging from nanometers to micrometers, into which BAPPs can be incorporated either on their surface or within their interior. Particles loaded with bioactive agents are generally integrated into scaffolds or hydrogels for tissue engineering, and they may also be employed directly for tissue repair [239]. The main components of assisted particles can be divided into bioceramic particles, polymeric particles, liposomes, extracellular vesicles, and metal-organic frameworks (MOFs).

#### Bioceramic particles

Bioceramic particles can be synthesized from a variety of inorganic components, primarily including silica, calcium carbonate, and calcium phosphate. BAPPs can be immobilized on the surfaces of bioceramic particles through surface adsorption or covalent anchoring. Furthermore, BAPPs may also be loaded into the interior of bioceramic particles through coprecipitation or macropore strategies.

Surface adsorption represents the predominant method for bioceramic particles to deliver BAPPs. For example, lactoferrin is adsorbed to the surface of hydroxyapatite through electrostatic interactions to facilitate bone regeneration [240]. Moreover, enhancing noncovalent interactions via binding systems can augment the ability of bioceramic particles to deliver BAPPs. Stable CaCO_3_ microspheres modified with casein were subsequently functionalized with heparin to adsorb BMP-2 for bone regeneration [241]. Additionally, BAPPs may be first encapsulated within secondary carriers before being adsorbed onto the surfaces of inorganic microspheres or nanoparticles. Xue et al. [242] initially incorporated BMP-2 within soy lecithin and then adsorbed this system onto calcium phosphate silicate microspheres for applications in bone tissue engineering. The results showed that the release of BMP-2 from the microspheres was significantly delayed due to the presence of soy lecithin [242].

Covalent anchoring represents an alternative strategy for delivering BAPPs to the surfaces of bioceramic particles. Functional groups are typically modified on inorganic carriers to serve as coupling sites for anchoring BAPPs. For example, the surface of MSNs was initially functionalized with amine groups using APTES, which were subsequently employed to couple a BMP-2 derivative peptide for bone tissue regeneration via the EDC/NHS system [243]. Another study applied the same method to covalently graft TGF-β3 onto the surface of MSNs for annulus fibrosus repair [244]. However, covalent conjugation through the EDC/NHS system is nonspecific. Therefore, specific coupling strategies need to be developed. Additionally, covalent conjugation may reduce the biological activity of peptides and proteins, potentially limiting their applications.

Coprecipitation, which involves blending bioactive agents with precursor solutions before the formation of microspheres, serves as a strategy for incorporating BAPPs inside bioceramic particles. Compared to surface adsorption, coprecipitation improves the delivery efficiency, ensuring the release of sufficient doses of bioactive factors necessary for tissue repair [245]. However, direct contact between bioactive peptides or proteins and reagents may affect their biological activity in this approach. Furthermore, regulating the non-covalent interactions between bioactive peptides or proteins and inorganic carriers is the key to controlled release.

Macropore strategies involve the fabrication of macroporous bioceramic particles that facilitate the incorporation of BAPPs into their interior. The mesopore diameters of mesoporous bioceramic particles are generally less than 7 nm, but the dimensions of bioactive proteins such as BMPs, IGFs, VEGF, and PDGF exceed 10 nm. Therefore, enlarging the size of pores is essential for enabling these bioactive proteins to penetrate the interior of the particles [246]. Zhang et al. [246] developed large-pore mesoporous silica particles with mesopore diameters exceeding 15 nm and utilized bovine serum albumin (BSA) as a model protein to assess loading capacities and release profiles. The results revealed that BSA could be successfully introduced into the particle and released in a sustained manner [246]. Another study produced enlarged-pored MSNs with mesopore diameters measuring 12.2 nm and incorporated acidic FGF (aFGF) into these nanoparticles [247]. Measuring nanoparticles containing aFGF were blended in polymer solutions to create polymer-shelled particles via electrospraying, and the results showed that this shell structure also promoted sustained protein release [247].

#### Polymeric particles

Polymeric microspheres and nanoparticles are generally fabricated from natural degradable polymers such as collagen, gelatin, alginate, and chitosan, as well as synthetic degradable polymers like PLGA, PCL, and PLA [248]. Utilizing various fabrication methods, BAPPs can be loaded on the surfaces or within carriers for tissue repair.

Adsorbing or coupling BAPPs to pre-prepared microspheres or nanoparticles constitutes one of the strategies employed for their delivery. On one hand, BAPPs can be directly loaded into microspheres or nanoparticles through immersion followed by desiccation, allowing them to penetrate carriers due to swelling [239, 249]. To enhance the interactions between carriers and bioactive peptides or proteins, binding molecules can be modified on the surfaces of microspheres and nanoparticles. Consequently, BAPPs tend to accumulate on the surfaces of these structures following surface modification. For instance, Chen et al. [250] developed GelMA microspheres using a microfluidic technique and modified their surfaces with fibronectin to immobilize PDGF-BB via this heparin-binding domain. Another study used laponite to modify the surface of microspheres for adsorbing BMP-2 aimed at bone induction [251]. On the other hand, covalently coupling bioactive peptides or proteins is an alternative method for delivering them onto organic microspheres and nanoparticles. For instance, vanillin methacrylate was employed to modify GelMA microspheres through copolymerization. **S**ubsequently, TGF-β3 was immobilized on these microspheres via dynamic covalent bonds formed between the aldehyde groups of vanillin methacrylate and amino groups of bioactive proteins [252]. The results indicated that the release of TGF-β3 from modified microspheres occurred at a relatively slower rate compared to unmodified GelMA counterparts while maintaining the bioactivity necessary for the regeneration of intervertebral discs [252].

BAPPs can be incorporated within organic microspheres and nanoparticles during the fabrication process. Emulsification and solvent evaporation are common strategies employed for integrating BAPPs in tissue repair applications. Specifically, BAPPs in the aqueous phase are added to the oil phase containing organic polymers, which are then emulsified through ultrasonication before being further mixed with the water phase to form emulsion droplets. Following the evaporation of organic solvents, these emulsion droplets solidify into microspheres or nanoparticles, thereby encapsulating bioactive peptides or proteins in their interior. This method has been widely utilized to produce PLGA microspheres and nanoparticles for delivering BAPPs in various tissue regeneration and repair, including cartilage [253], intervertebral discs [254], tendons [255], and cochlear ribbon synapses [256]. Additionally, polyelectrolyte complexation can be used to encapsulate bioactive peptides or proteins during fabrication, typically utilizing heparin and PLL. Bioactive peptides or proteins were previously adsorbed onto negatively charged heparin before being added to a positively charged PLL solution under constant vortexing to obtain nanoparticles suitable for tissue repair [257]. Furthermore, phase separation and microfluidic techniques may also be applied to construct microspheres that load BAPPs [258]. In this approach, bioactive peptides were added to the water phase containing the polymers before incorporation into an oil phase. The introduction of crosslinkers facilitates particle formation from polymers while incorporating BAPPs within these structures. However, direct contact between crosslinkers and BAPPs may lead to a decrease in biological activity.

Electrospraying can be used to encapsulate BAPPs within microspheres and nanoparticles. Compared to other techniques for developing carriers, the size and charge of the particles can be regulated by adjusting the flow rate and electric voltage [259]. Before the electrospraying process, bioactive peptides or proteins are combined with a polymer precursor solution and subsequently transferred into a needle to produce microspheres or nanoparticles. For example, Nagase et al. [260] mixed an aqueous phase containing bFGF with an oil phase comprising PLGA, then ultrasonicated the blend solution to prepare a W/O emulsion, which was utilized to fabricate bFGF-incorporated nanoparticles via electrospraying. The resulting nanoparticles were collected and placed on prepared hepatocyte sheets to promote vascularization and maintain cell survival in hepatic tissue engineering [260]. Additionally, modifying electrospraying particles with inorganic components is a simple and efficient strategy for optimizing the delivery of BAPPs without complex chemical reactions or expensive equipment. Zhang et al. [261] added VEGF to a laponite solution before mixing it with an RGD-modified alginate solution. This mixture was subsequently used to prepare microspheres through electrospraying for endodontic regeneration. The results indicated that the release of VEGF from laponite/RGD-modified alginate microspheres dramatically decreased compared to that from pure RGD-modified alginate microspheres [261]. Since electrospraying and electrospinning use similar equipment, microspheres or nanoparticles generated by electrospraying can be directly coated onto electrospun fiber scaffolds. For instance, PLGA microspheres encapsulating NGF were sandwiched between two layers of electrospun fibers by integrating both techniques, facilitating long-term controlled release of NGF for spinal cord injury repair [255].

The capacity of microspheres or nanoparticles to deliver and release multiple factors can be tailored to meet the requirements of complex tissue regeneration. One potential strategy involves the fabrication of core–shell particles, where one bioactive agent is loaded into the core structure while another agent is incorporated into the shell phase [262]. For example, researchers encapsulated OGP in PLGA microspheres and closed the surface pores by treating them with acetonitrile. Subsequently, these microspheres were coated with a LBL membrane for delivering BMP-2 [263]. The sequential release of OPG and BMP-2 from multilayered microspheres was employed to induce osteogenic differentiation and promote bone regeneration [263]. Additionally, encapsulating one factor along with small carriers containing another agent within larger carriers slows the simultaneous release of two different bioactive factors. Xu et al. [264] fabricated relatively small BMP-2-incorporated alginate microspheres through an initial electrospraying process followed by coating their surface with chitosan. These resulting microspheres were then blended with an alginate solution containing SDF-1 to create larger microspheres via a second electrospray. The burst release of SDF-1 contributed to stem cell recruitment, while the sustained release of BMP-2 induced osteogenic differentiation in stem cells for bone regeneration. Another study incorporated kartogenin within MSNs before adding them to a chitosan solution containing PDGF-BB to fabricate composite microspheres aimed at repairing osteoarticular lesions [258]. This complex structure enables substantial amounts of PDGF-BB to be released during early stages while allowing kartogenin in MSNs to be released gradually over time [258].

#### Liposomes

Liposomes are vesicles composed of an aqueous core enclosed by a lipid bilayer membrane containing phospholipids and cholesterol [265]. BAPPs are generally incorporated into the aqueous phase for tissue repair [266]. A study integrated a BMP-2-derived peptide into maleimide-modified liposomes and chemically linked these liposomes to electrospun PLLA scaffolds for bone regeneration [267]. The results showed that approximately 20% of the BMP-2-derived peptide was released after 6 d [267]. Additionally, the inner section of the lipid layer can encapsulate hydrophobic drugs, while BAPPs can be further loaded into the aqueous phase within liposomes, thus facilitating multidrug delivery [268]. Given that bioactive peptides or proteins are released slowly from liposomes, other bioactive factors may also be directly loaded into the scaffold matrix to enable sequential release at different time points [269]. Furthermore, the composition of liposomes can be modified to impact specific functions. For instance, liposomes fused with activated neutrophil membranes were subsequently utilized to encapsulate keratinocyte growth factor (KGF) for the treatment of ulcerative colitis [270]. The addition of an activated neutrophil membrane allows these composite carriers to target inflamed bowel regions, enabling KGF to be released and exert therapeutic effects at diseased sites [270].

#### Extracellular vesicles

Extracellular vesicles, including exosomes, microvesicles, and apoptotic bodies, are lipid bilayer membrane-bound vesicles that are secreted by cells through paracrine mechanisms rather than being artificially synthesized. They naturally contain a variety of bioactive proteins as well as other bioactive agents, such as small and long coding and noncoding RNAs (e.g., mRNA, miRNA, lncRNA) [271]. Extracellular vesicles, particularly exosomes, have gained significant attention for tissue repair due to the valuable cargo they deliver [272]. In recent years, engineered exosomes have been developed by introducing plasmid DNA encoding bioactive peptides or proteins to achieve specific functions [273, 274]. Further research may focus on the direct loading of bioactive peptides or proteins into extracellular vesicles for tissue repair. Moreover, combining liposomes encapsulating BAPPs with extracellular vesicles presents a promising strategy for tissue repair.

#### MOFs

MOFs are synthesized by bridging metal ions with organic ligands to form specific structures that exhibit multiple properties, including high porosities, large surface areas, and flexible designs [275]. BAPPs can be delivered to MOFs through 4 methods: surface adsorption, pore entrapment, coprecipitation, and covalent conjugation [276]. Coprecipitation refers to the process in which bioactive peptides or proteins are loaded in situ within MOFs during synthesis, thereby forming a shell that inhibits the release of these bioactive agents. Compared to surface adsorption and pore encapsulation, both relying solely on noncovalent interactions for delivery, coprecipitation may be a better strategy as MOFs act as shells that effectively block the release of bioactive peptides or proteins. Although covalent conjugation can also facilitate long-term delivery, it may compromise the biological activity of BAPPs.

Among various MOFs, zeolitic imidazolate framework-8 (ZIF-8) has been extensively utilized for drug delivery in tissue regeneration [277, 278]. ZIF-8 is typically synthesized by mixing zinc salt with 2-methylimidazole at room temperature. In recent years, ZIF-8 has been employed to deliver bioactive peptides or proteins for tissue engineering. For example, BMP-2 was loaded into ZIF-8 through a one-pot rapid coprecipitation method, which was subsequently blended with PCL to fabricate electrospun fiber scaffolds for bone tissue engineering [279]. During the initial 4 h, approximately 12% of BMP-6 was released, however, this increased to about 35% after 30 d [279]. Furthermore, ZIF-8 can be modified with other molecules to improve sustained release or drug loading. For instance, Jiang et al. [280] developed PDA-modified ZIF-8 for delivering BMP-2 in bone regeneration and observed a significant improvement in encapsulation efficiency compared to unmodified ZIF-8. Therefore, ZIF-8 and its derivatives are ideal carriers for BAPPs after coprecipitation for tissue repair.

### Nanotubes

Nanotubes possess a hollow tubular structure, with their surfaces suitable for the adsorption of BAPPs, while the interior facilitates encapsulation. Commonly utilized nanotubes BAPPs delivery include halloysite nanotubes (HNTs), CNTs, TiO_2_ nanotubes (TNTs), and self-assembling nanotubes.

#### HNTs

HNTs are natural nanoclusters characterized by a negatively charged outer surface due to the presence of SiOH groups, while their inner surface exhibits a positive charge attributed to AlOH groups. Consequently, HNTs can be employed for the delivery of BAPPs in tissue regeneration via noncovalent interactions, including electrostatic forces and hydrogen bonding. Abdulmalik et al. [281] introduced exendin-4 (Ex-4), a negatively charged analog of glucagon-like peptide-1, into the open lumen of HNTs, which were subsequently blended with PCL/CA spinning solution to fabricate nanofiber scaffolds for tendon regeneration. The bilateral ends of HNTs containing Ex-4 were sealed with polymer solution during electrospinning. Results showed that the encapsulation efficiency improved from 34.4 to 86.58% when using HNTs-based methods compared to directly loading Ex-4 into electrospun fibers. Additionally, the initial release of Ex-4 was dramatically optimized from 75 to 40% with the aid of HBTs [281]. Furthermore, both the inner and outer surfaces of HNTs may be functionalized or modified to enhance BAPP efficacy in promoting tissue regeneration.

#### CNTs

CNTs are composed of graphene arranged in a cylindrical structure and are predominantly utilized for intracellular drug delivery due to their capacity for cellular internalization[282]. Although BAPPs usually interact with cell membrane receptors to exert their effects, CNTs have been less extensively investigated as secondary carriers for BAPPs. However, they can be directly used to fabricate scaffolds that deliver peptides and proteins for tissue regeneration. Tanaka et al. [283] employed CNT porous blocks as scaffolds to incorporate BMP-2 for bone regeneration. The results revealed that the CNT porous block adsorbed and released more BMP-2 compared to interconnected porous HA ceramics [283]. Further modifications of CNT-based scaffolds may improve their efficacy in delivering BAPPs for tissue repair.

#### TiO_2_ nanotubes

Titanium-based implants are widely utilized for the repair of bone and dental structures [284]. The titanium surface can be modified through an electrochemical anodizing process to create TNTs, which serve as delivery platforms for bioactive peptides or proteins. For example, researchers introduced BMP-2 or a BMP-2-derived peptide into TNTs and subsequently implanted the scaffolds in vivo [285, 286]. Compared to basic pure scaffolds, those loaded with BMP-2 or BMP-2-derived peptides significantly promoted bone tissue regeneration [285, 286]. Another study incorporated BMP-2 into bone-shaped TNTs, sealing the upper surface with a VEGF-loaded hydrogel [287]. The simultaneous release of BMP-2 and VEGF markedly facilitated bone regeneration [287]. Moreover, TNTs can be optimized as fundamental carriers by changing their physical characteristics (such as diameter and length) and employing alternative delivery methods to enhance their delivery capacities [288, 289]. For instance, TNTs were modified with PDA coatings, which were then used to incorporate IL-4 for improved bone regeneration [286]. Additionally, secondary carriers containing bioactive agents may be encapsulated within TNTs to optimize their release.

#### Self-assembling nanotubes

Self-assembling nanotubes are formed through the self-assembling of small molecules via noncovalent interactions, which possess the ability to deliver BAPPs. Zhou et al. [290] fabricated Janus base nanotubes (JBNTs) by assembling DNA-based monomers utilizing hydrogen bonding, π-π stacking, and hydrophobic interactions. These JBNTs were then used to create JBNT nano matrices with TGF-β1 or matrilin-3 sandwiched among multiple JBNTs. Using a layer-by-layer method, TGF-β1 was encapsulated within the inner layer of the JBNT nano matrix for chondrogenic differentiation, while marine-3 was loaded onto the outer layer to stimulate the cartilage microenvironment [290]. The results revealed that the JNBT nano matrix effectively prevented the leakage of TGF-β1 after 15 d of incubation in vitro, thus localizing the bioactive protein to exert its effects [290]. In addition to nucleic acid-based nanotubes, peptide- or protein-based nanotubes can also be utilized for drug delivery [291, 292]. Furthermore, peptide- or protein-based nanotubes demonstrate potential for delivering BAPPs in tissue repair.

### Two-dimensional nanomaterials

Two-dimensional nanomaterials generally possess large surface areas that facilitate the immobilization of bioactive peptides or proteins on their surface. These nanomaterials are primarily composed of inorganic materials, including two-dimensional nanoclays, two-dimensional graphene family materials, black phosphorus nanosheets (BPNTs), and MXene nanosheets. Additionally, organic polymers can be engineered into nanosheets, which are predominantly utilized for encapsulating BAPPs.

#### Two-dimensional nanoclays

Two-dimensional nanoclays consist of alternating tetrahedral and octahedral sheets, characterized by large surface areas and favorable adsorption properties for drug delivery [293]. Sheet-based nanoclays can be divided into natural nanocalys and synthetic varieties. Although natural nanoclays such as montmorillonite and kaolinite have been employed for the delivery of bioactive peptides or proteins, synthetic nanoclays exhibit more uniform morphologies and properties compared to their natural counterparts [294, 295]. Laponite is one of the most extensively utilized synthetic nanoclays for delivering bioactive peptides or proteins. It features a sheet silicate crystal structure in which a central octahedral sheet containing Mg^2+^ is inserted into two tetrahedral silica sheets. These materials exhibit positive charges on the edges and negative charges on the surfaces, enabling them to load various bioactive peptides or proteins for tissue regeneration, including bone [296], wounds [297], and skeletal muscle [298]. For example, Quint et al. [299] loaded IGF-1 to laponite, which was subsequently incorporated into GelMA hydrogels for skeletal muscle tissue engineering. The pure GelMA hydrogel released nearly all the IGF-1 within 7 d, whereas the GelMA hydrogel containing laponite with IGF-1 exhibited a sustained release over 15 d. Additionally, laponite releases several ions, including magnesium, lithium, and silicate ions, which are beneficial for tissue regeneration [300].

#### Two-dimensional graphene family materials

The two-dimensional graphene family primarily comprises graphene, GO, and reduced graphene oxide (rGO), all of which possess large surface areas and then free π electrons. Additionally, BAPPs with low solubility, partial hydrophobicity, or a positive charge tend to adsorb onto their surface [301]. Compared to graphene and rGO, GO exhibits multiple functional groups, including carboxyl, hydroxyl, and epoxide groups, resulting from oxidation. Therefore, it shows greater potential for delivering BAPPs through various noncovalent interactions, such as hydrogen bonding, π-π interactions, and ionic interactions [302]. GO has been widely used to deliver BAPPs for tissue regeneration [303, 304]. For example, TGF-β3 was physically loaded onto GO before being incorporated into a photopolymerizable poly-D, L-lactic acid/polyethylene glycol (PDLLA) hydrogel for chondrogenic differentiation [305]. GO significantly delayed the release of TGF-β3 over several weeks. Furthermore, the functional groups on GO enable covalent conjugation with BAPPs. Wu et al. [306] chemically coupled a BMP-2-derived peptide, P24 to GO using the EDC/NHS method and then introduced the GO-P24 complex to silk fibroin electrospun fiber scaffolds modified with chitosan for bone regeneration. The results showed that these scaffolds did not release more than 50% of the material even after 21 d [306].

#### BPNTs

BPNTs are potential candidates for the delivery of bioactive peptides or proteins due to their strong surface and noncovalent interactions, including π-π stacking, electrostatic interactions, and hydrophobicity. In one study, VEGF was initially incorporated into BPNTs before being integrated into DNA hydrogels [307]. These hydrogels were subsequently injected into 3D-printed PCL scaffolds for bone regeneration, and the results demonstrated that the composite scaffolds containing VEGF-loaded BPNTs exhibited sustained release without burst release [307]. Currently, BPNTs are primarily utilized for delivering small molecule non-amino acid drugs for tissue regeneration, such as deferoxamine [308, 309] and dexamethasone [310]. Research on the delivery of bioactive peptides or proteins by BPNTs remains limited. Considering their excellent biocompatibility and photothermal conversion capacity, BPNTs have shown great potential for tissue repair. Rational surface modifications, such as surface coatings to BPNTs, may serve as effective strategies to improve the capacity for loading content or sustained release.

#### MXene nanosheets

MXene nanosheets also exhibit biocompatibility and high efficiency in near-infrared (NIR)-thermal conversion. They have a large surface area suitable for loading bioactive agents; however, their low drug loading limits their potential as a delivery platform [311]. Therefore, the surfaces of MXene nanosheets are typically modified to enhance the delivery of bioactive peptides or proteins. Yang et al. [312] ultrasonicated MXene bulks to fabricate MXene nanosheets and then modified these with MoS2 for antibacterial activity and PDA coatings to functionalize FGF-21. The results suggested that the FGF-21-loaded nanosheets effectively promoted wound healing in vivo. As emerging biomedical delivery platforms for tissue repair, further research is essential for the optimal modification of MXene nanosheets for delivering BAPPs in tissue regeneration.

#### Layered double hydroxide (LDH) nanosheets

LDH nanosheets are biocompatible and degradable, exhibiting unique properties, including large surfaces and controllable metal components and structures, which have been used in tissue repair. Lv et al. [313] fabricated a MgFe-LDH nanosheet modified with chondroitin sulfate to load BMP-2, subsequently incorporating the BMP-2-loaded nanosheet along with PDGF-BB into chitosan/silk fibroin hydrogels for bone regeneration. The results showed that 80.03% of the PDGF-BB in the hydrogel matrix was released within the first 7 d, while the BMP-2 adsorbed onto the nanosheet was released more slowly, with 74.51% of the loaded BMP-2 being released after 35 d [313].

#### Polymer-based nanosheets

While two-dimensional carriers are mainly fabricated from inorganic substances, polymers can also be used to develop nanosheets for the delivery of bioactive peptides or proteins. For example, researchers have created PLLA nanosheets via a simple fabrication process involving spin-coating and peeling techniques, subsequently sandwiching BMP-2 between two layers of PLLA nanosheets for bone regeneration [314]. BMP-2 was released slowly in vitro over 2 months, and the sandwich-like nanosheets containing BMP-2 dramatically promoted bone repair in vivo [314]. Furthermore, multilayer PLLA nanosheets were developed to deliver FGF-2 for the repair of femoral defects, and the findings indicated that these scaffolds gradually released FGF-2 over 2 weeks when implanted subcutaneously in vivo [315]. Therefore, polymer-based nanosheets hold considerable promise for the delivery of BAPPs, warranting further investigation into nanosheets derived from other polymers.

### Nanoparticle engineered cells

Recent advancements in drug delivery have increasingly focused on nanoparticle-engineered cells. Among various cell types, neutrophils are emerging as promising carriers for drug delivery due to their unique response to inflammation, chemotaxis, and transmigration [316]. Nanoparticles containing bioactive peptides or proteins are directly internalized by live neutrophils and subsequently used for tissue regeneration. For example, PTH 1-34 was preloaded on PLGA nanoparticles, which were then internalized by neutrophils that highly expressed CXCR4 after 1 h of incubation [317]. Under the influence of CXCR4/CXCL12 signaling, neutrophils carrying PTH 1-34-loaded nanoparticles migrate to the bone marrow to promote bone regeneration [317]. However, the internalization of nanoparticles may affect neutrophil function, and the activity of BAPPs may also be impacted during this process. Strategies have been developed to load drugs onto the surfaces of neutrophils [318, 319], making it a viable approach to couple particles containing bioactive peptides or proteins on the surface of neutrophils for tissue repair via targeted delivery.

## Stimuli-responsive delivery of BAPPs

**Fig. 8:**
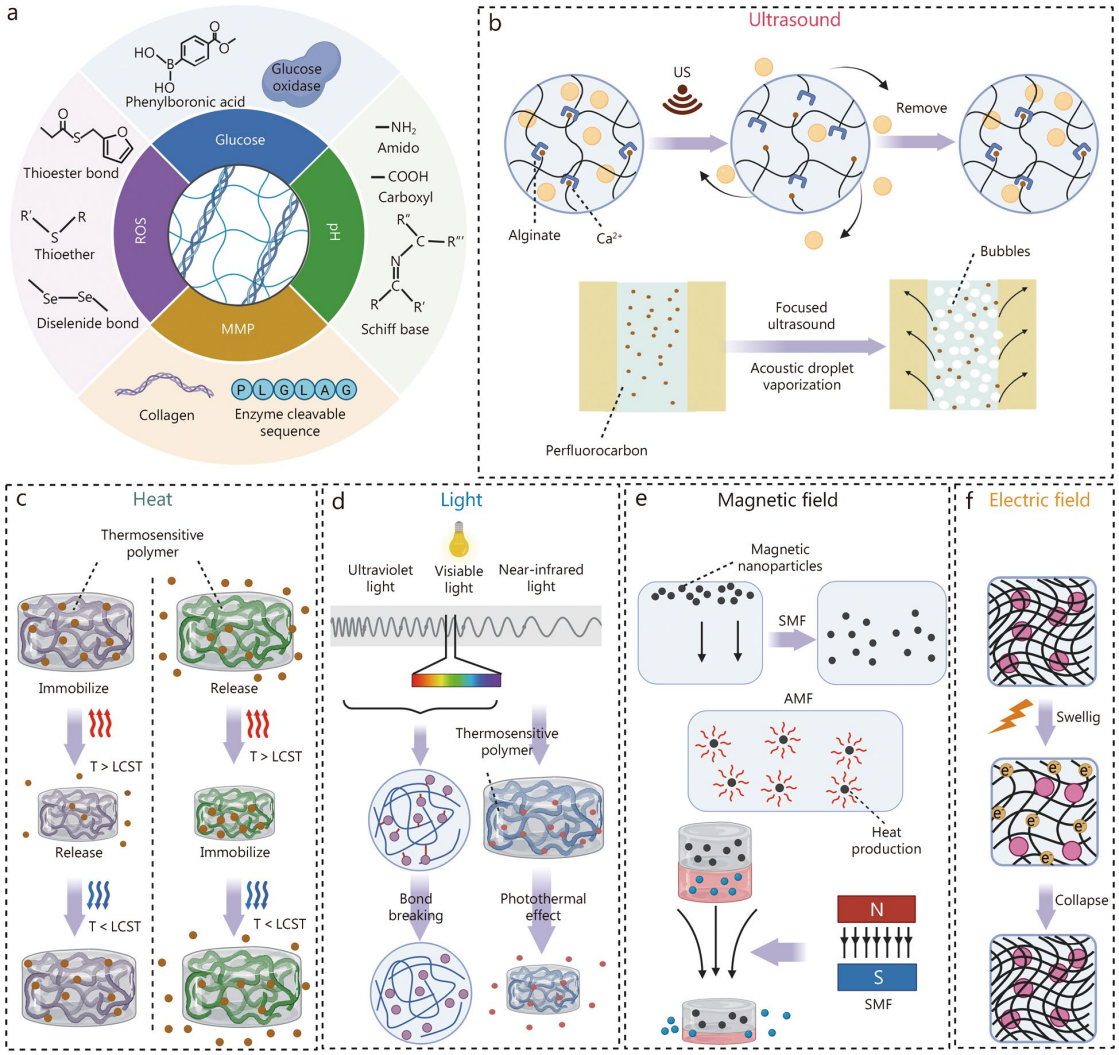
Stimuli-responsive delivery of bioactive peptides and proteins (BAPPs) for tissue repair. **a** Endogenous stimuli from the microenvironment (glucose, ROS, the MMP, and pH) and the corresponding chemical bond breaks in response. **b** The coordination bond between calcium and alginate is broken under ultrasonic stimulation; ultrasound stimulates PFC droplets to vaporize into microbubbles, which triggers response release. **c** When the temperature is higher than the lower critical solution temperature (LCST), the volume of the thermosensitive polymers decreases. Then, the micromolecular agents are squeezed out, while the macromolecular agents are fixed by contracted thermosensitive polymers. **d** The photosensitive group is broken under ultraviolet and visible light stimulation; near-infrared light produces photothermal effects that trigger volume shrinkage of thermosensitive polymers. **e** AMF triggers magnetic nanoparticles to generate heat; SMF triggers magnetic nanoparticles to move and induces hydrogel deformation. **f** When an electric current is applied, the drug-loaded conjugated polymer undergoes a redox reaction, which changes its properties, leading to the release of BAPPs. ROS reactive oxygen species, MMP matrix metalloproteinases, US ultrasound, SMF stationary magnetic field, PFC perfluorocarbon, AMF alternating magnetic field

On-demand release through stimuli-responsive delivery represents a promising strategy for BAPPs, as it addresses the requirements of tissue regeneration while minimizing adverse effects associated with high doses of BAPPs. Both endogenous and exogenous stimuli can be exploited to precisely control the release of BAPPs (Fig. 8). Endogenous stimuli in organisms are mediated by factors such as glucose, ROS, enzymes, and pH, while exogenous stimuli are influenced by ultrasound, heat, light, magnetic fields, electric fields. In the absence of external stimuli, BAPPs can be preserved in delivery platforms to avoid off-target effects and side effects caused by inappropriate release timing [4]. Conversely, upon exposure to specific stimuli, BAPPs can be released due to the disintegration of delivery platform materials via various mechanisms, including protonation, hydrolytic cleavage, and molecular/supramolecular conformational changes [320].

### Endogenous stimuli from the microenvironment

The tissue microenvironment has significant characteristics in some special diseases like diabetes, and pathological changes such as changes in or the appearance of pH, ROS, and specific enzymes, occur in the microenvironment. Therefore, developing delivery strategies to respond to these endogenous stimuli will help promote tissue regeneration.

#### Glucose

Glucose-responsive materials exhibit widespread application prospects due to the prevalence of diabetes, and they can be categorized into 2 types based on phenylboronic acid (PBA) or glucose oxidase (GOx) [321]. Elevated glucose levels lead to an increase in advanced glycation end products and promote the generation of excessive ROS, which adversely affects tissue repair. Consequently, responsive modulation of the microenvironment with high glucose at the injury site positively influences tissue regeneration. Furthermore, insulin plays a crucial role in regulating the function of epidermal and dermal skin cells, as well as blood vessels and immune cells, thereby further promoting repair [322, 323].

PBA can covalently bind to polymers and react with polyhydroxy substances to form glucose-responsive PBA ester bonds, while the catechol structure of the PBA ester impacts glucose responsiveness to the materials. In microenvironments characterized by high glucose levels, glucose competitively binds to borate anions through its diol functional groups, leading to the decomposition of covalent borate esters [324]. PBA-based materials have been widely used to fabricate delivery platforms for BAPPs [325]. For example, Zhao et al. [326] developed a glucose-responsive film via LBL self-assembling by alternately depositing PBA-grafted γ-PGA and PVA, enabling the delivery of lysine-proline-valine (KPV) peptide for inflammation inhibition and EGF for tissue repair. The KPV peptide was incorporated into the bottom layer, while EGF was loaded into both the middle and upper layers [326]. Upon exposure to a gradient of glucose in PBS, the film exhibited glucose-dependent degradation, resulting in a controlled release of KPV peptide and EGF in response to glucose levels [326]. The KPV peptide from the bottom layer was rapidly released within 1 − 3 d to exert an anti-inflammatory effect during the early stages of wound healing, while the EGF from the middle and upper layers was continuously released over 7 d to promote subsequent production of collagen [326]. Additionally, Li et al. [325] synthesized a glucose-responsive crosslinker, N^1^-(4-boronobenzyl)-N^3^-(4-boronophenyl)-N^1^,N^1^,N^3^,N^3^-tetramethylpropane-1,3-diaminium (TSPBA), by using 4-bromomethyl PBA and N, N, N’, N’-tetramethyl-1,3-propanediamine. TSPBA reacted with PVA to form PBA ester bonds. Therefore, IL-4 loaded within this matrix could be released in response to elevated glucose levels, thereby modulating the immune microenvironment for bone regeneration [325].

The introduction of GOx is another strategy for the development of glucose-responsive materials, as GOx catalyzes the conversion of glucose into gluconic acid, which generates electrostatic repulsions through protonation and causes the matrix to swell, thereby facilitating drug release [327, 328]. For example, Chen et al. [328]. Added GOx and insulin to the MOFs, where glucose triggered the degradation of the MOFs and subsequent release of insulin to manage diabetes or macular disease. Additionally, catalase was incorporated into the MOFs to mitigate the toxicity associated with H_2_O_2_ produced by the reaction between Gox and glucose [328].

#### ROS

ROS participate in many physiological processes and immune responses in the human body, and their levels are often elevated in various pathological conditions, such as tumors, chronic wounds, myocardial infarction, postoperative tissue adhesion, and other diseases [329]. In comparison to other stimulus-responsive systems, ROS-responsive materials can effectively protect cells from oxidative stress by eliminating excessive ROS while responding to stimuli [330, 331].

ROS can influence the solubility of poly(propylene sulfide) (PPS), sulfide-containing polymers, and tellurium- and selenium-containing polymers. In oxidative stress microenvironments, hydrophobic sulfides can be converted into hydrophilic sulfoxides and sulfones, thus facilitating the release of bioactive drugs [332]. For example, Martin et al. [333] used poly (thioketal β-amino amide) as the polycation and poly(acrylic acid) as the polyanion to fabricate a LBL multilayer system for delivering BMP-2 in bone regeneration. This multilayer contains the ROS-sensitive thioketal units in the polycation, allowing for BMP-2 release following the oxidation of thioester-based polymers by ROS [333]. In another study, Yuan et al. [334] utilized diselenide bonds to conjugate NGF to ruthenium nanoparticles while combing NGF with the brain-targeting peptide RVG to form nanoclusters aimed at brain-targeted delivery. The diselenide bonds undergo cleavage upon exposure to elevated levels of ROS at the lesion site, thus releasing NGF for nerve tissue repair [334].

ROS can also directly cleave the chemical linkages of poly(thioester) (TK), PBA ester poly(proline), and other ester-containing polymers, leading to their degradation in a ROS-rich environment [332]. For instance, researchers developed a ROS-responsive injectable PEG-TK hydrogel containing thioester bonds for the delivery of EGF aimed at wound repair [335]. The TK bonds in the PEG-TK hydrogel not only removed excessive ROS in the microenvironment but also facilitated EGF release in response to ROS levels, thereby promoting tissue regeneration [335]. Additionally, TSPBA cross-linked hydrogels exhibit responsiveness to both ROS and glucose [336]. Researchers have utilized TSPBA to react with PVA, resulting in an injectable hydrogel designed for delivering bFGF, which can be released upon exposure to elevated concentrations of ROS surrounding ischemia–reperfusion myocardium [337]. Higher concentrations of ROS accelerated the release of bFGF from the hydrogel in vitro, and the therapeutic effects of the ROS-responsive hydrogel loaded with bFGF were validated using mouse and pig models of ischemia–reperfusion injury [337].

The application of ROS-responsive release materials still faces several technical challenges and limitations. Since low levels of ROS are essential for normal metabolic activity, uncontrolled removal of ROS may lead to adverse effects. Therefore, an effective release system must ensure that normal concentrations of ROS do not trigger the release of therapeutic drugs while allowing for drug release or enhancing the ability of materials to regulate ROS under pathological conditions [338]. Furthermore, the specific species and concentration of ROS in various pathological states remain largely unknown, making it challenging to align the degradation rate of materials with ROS level in vivo.

#### Enzyme

Some enzymes, such as matrix metalloproteinases (MMPs), cysteine proteases, and hyaluronidase, are generally overexpressed in the microenvironment during the process of tissue damage and regeneration [339, 340]. Among these, MMPs, extracellular matrix remodeling endopeptidases capable of cleaving various matrix polymers such as collagen, elastin, gelatin, and casein, are most frequently utilized as triggers for stimulus responses [341]. Therefore, polymers containing natural cleavage sites for MMPs can be employed to develop MMP-responsive delivery systems. Yang et al. [342] synthesized hydrogels using GelMA and poly(sodium 4-styrene sulfonate) (PSS), synthetic analogs of heparin, to fabricate VEGF delivery. GelMA is degradable in response to elevated levels of MMPs within inflammatory environments. Additionally, negatively charged PSS effectively adsorbs positively charged VEGF [342]. In vitro, release experiments demonstrated that VEGF was released through an initial burst release followed by a slow sustained release regardless of the presence or absence of MMPs, but the release accelerated upon the addition of MMPs [342].

The incorporation of an MMP-clearable sequence into BAPPs, along with the immobilization of fusion peptides onto delivery platforms, are also potential strategies for the fabrication of MMP-responsive systems. For example, Fan et al. [343] introduced the MMP-2/9-clearable sequence PLGLAG and glutathione-S-transferase (GST) into bFGF, thereby developing a recombinant fusion protein designated GST-TIMP-bFGF. This fusion protein was then covalently conjugated to glutathione-grafted collagen hydrogels through the action of GST [343]. In regions affected by cardiac infarction, overexpressed MMP-2/9 can cleave the TIMP peptide like bFGF and GST, facilitating the on-demand release of bFGF [343]. Moreover, the TIMP peptide serves as a competitive substrate of MMPs and can inhibit excessive degradation of the cardiac matrix following myocardial infarction [343]. Another study introduced an MMP-clearable sequence at both ends of a bioactive peptide while additionally incorporating thiol-functionalized crosslinkers at each end, thus developing C-IPESLRAG-bioactive peptide-IPESLRAG-C-G to enable conjugation with norbornene-functionalized PEG [344]. The PEG hydrogels exhibited degradation and subsequent releases of bioactive peptides in response to MMPs presence, with release kinetics aligning closely with the degradation kinetics of hydrogels [344].

#### pH

The release of BAPPs can be precisely regulated by the local pH fluctuations that occur in the microenvironment during tissue regeneration. For example, acute wounds exhibit a decrease in pH due to temporary acidosis, while chronic wounds demonstrate an increase in pH resulting from persistent inflammation and bacterial infection [345]. Furthermore, certain diseases such as fractures, osteoarthritis, and osteomyelitis, are characterized by localized pH alternations, thereby presenting significant application prospects for pH-responsive materials.

pH changes can induce the protonation of acid-sensitive chemical groups, such as amino groups and carboxyl groups, which undergo structural and hydrophobic transformations in a pH-dependent manner to release bioactive drugs on demand [320, 346]. For example, alginate possesses a large number of carboxyl groups in its polymer framework, which are converted into negatively charged carboxylate ions in alkaline conditions, leading to pH-responsive expansion that accelerates drug release [345]. Researchers used alginate hydrogels cross-linked with calcium ions for the delivery of IGF-1 aimed at tendon regeneration and found that the hydrogel swelled under low pH, thereby promoting the release of IGF-1. Additionally, pH changes can trigger the cleavage of acid-sensitive bonds, including ortho-esters, Schiff bases, acetal/ketal moieties, hydrazone/oxime bonds, silyl ethers, and vinyl ethers [346, 347]. Zhao et al. [348] developed a chitosan-PVA hydrogel based on low pH-responsive benzamide (Schiff base) for insulin delivery in wound healing, indicating that a low pH environment promoted insulin release.

Different tissues may exhibit distinct optimal healing pH values, and the pH at the injured site can undergo multiple stages of change throughout various healing periods, posing challenges for the application of pH-responsive release systems. The utilization of implant materials carpetable of real-time monitoring of dynamic pH changes in the wound microenvironment and responding with drug release represents a promising strategy. Several approaches have been developed to access pH at injury sites, including materials integrated with pH sensors [349], wound dressings mixed with pH-indicating dyes [350], and pH-sensitive fluorescent probes [351].

### Exogenous stimuli by artificial control

Delivery strategies that rely on endogenous stimuli are limited due to a lack of disease-specific cues and large variability among individuals. Therefore, methods involving exogenous stimuli are important supplements for the precise delivery of BAPPs. Various exogenous stimuli, including ultrasound, heat, light, magnetic field, and electric field, can be employed to noninvasively regulate the release of BAPPs for tissue repair.

#### Ultrasound

Ultrasound is a well-established and promising medical technology that has been extensively used in biomedical therapy and diagnosis. It effectively penetrates complex media, allowing for precise localization in targeted areas [352]. Drug release can be accurately regulated through ultrasound-induced thermal and nonthermal effects [353]. High-intensity focused ultrasound is employed to generate thermal effects for drug delivery in tumor treatment [354]. However, the application of high-intensity focused ultrasound often results in tissue damage, limiting its use in the field of tissue repair. The nonthermal effects of ultrasound are mediated by ultrasonic pressure, acoustic flow, shock waves, liquid microjets, and oscillation or cavitation, which can be adapted to control the release of BAPPs.

Ultrasound can induce the cleavage of ultrasound-responsive bonds, thereby leading to the release of BAPPs. For example, ultrasound can disrupt the coordination between calcium ions and carboxyl groups. Consequently, researchers have used ultrasound-responsive calcium cross-linked alginate hydrogels to deliver ultrashort peptides containing SESSE for M2 macrophage polarization [355]. In another study, Shan et al. [356] encapsulated PLGA nanoparticles pre-loaded with NGF into alginate microspheres and subsequently introduced vitamin B12 along with these microspheres into a matrix of a calcium cross-linked alginate hydrogel. The results showed that ultrasonic treatment significantly accelerated the release rates of both drugs, while multilevel encapsulation enabled sustained release of NGF over one month[356]. Additionally, ultrasound could trigger a reversal reaction in the Diels–Alder linker; thus, the chitosan hydrogel formed via this cross-linking could release BSA under ultrasound stimulation [357].

Ultrasound can induce perfluorocarbon (PFC) droplets to vaporize into microbubbles through a process known as acoustic droplet vaporization [358]. Based on this property, Dong et al. [359] developed an acoustically responsive scaffold composed of a fibrin hydrogel infused with a bFGF-loaded, sonosensitive emulsion featuring a water-in-PFC-in-water (W1/PFC/W2) structure. In this configuration, bFGF was encapsulated with the W1 phase and could be released via PFC vaporization induced by ultrasound. The same research group also fabricated acoustically responsive scaffolds for bFGF delivery by incorporating perfluorohexane or perflurooctane into the phase-shift emulsion [358].

Although ultrasound offers the advantages of high penetration and noninvasiveness, it can also produce excessive ROS during the release process and may heat tissues, potentially causing damage [360]. Therefore, optimizing ultrasonic parameters remains a critical concern. High-frequency ultrasound is often used for diagnostic purposes, while frequencies below 1 MHz are utilized for drug delivery. To mitigate thermal effects and prevent tissue damage, the Food and Drug Administration (FDA) mandates that the mechanical index ratio, defined as the peak negative pressure divided by the center frequency, should be less than 1.9 [361].

#### Heat

Thermosensitive polymers can undergo phase transitions in response to temperature changes and are divided into two groups: polymers with lower critical solution temperature (LCST) and those with upper critical solution temperature (UCST). The solubility of LCST-type polymers decreases as the temperature rises, whereas the solubility of UCST-type polymers increases with elevated temperature. Polymers exhibiting LCST behavior are commonly utilized to fabricate heat-responsive delivery platforms. Below their LCST, these hydrophilic polymers exist in an expanded state due to hydrogen bonding between water molecules and the polymers. However, when the temperature exceeds the LCST, they become hydrophobic and contract [362]. Therefore, the shrinking or swelling of thermosensitive delivery platforms facilitates the release of BAPPs.

BAPPs can be preloaded onto thermosensitive platforms in a swollen state, and an increase in temperature beyond the LCST induces shrinkage of the thermosensitive platform, facilitating accelerated release. For example, Chen et al. [363] used a thermosensitive poly(N-isopropyl acrylamide) (pNIPAM) hydrogel loaded with FGF-2 to modify porous inverse opals, which was then introduced to a chitosan hydrogel for infectious wound healing. The temperature at the site of infection exceeded the LCST, leading to the contraction of the pNIPAM hydrogel and subsequent release of FGF-2. Additionally, both the structural color and characteristic reflection peak of the composite inverse opal particles exhibited blueshift upon shrinkage of the pNIPAM hydrogel, enabling real-time monitoring of FGF release [363]. In another study, researchers combined inverse opals with pNIPAM hydrogel containing VEGF for diabetic wound healing. The release rate of VEGF served as an indicator for monitoring pH changes and infections surrounding diabetic wounds [364]. Given that pNIPAM is intrinsically non-biodegradable, it can be modified through copolymerization with degradable polymers such as gelatin and chitosan, and these thermosensitive platforms may also facilitate the incorporation of bioactive agents for tissue repair [365, 366].

The interactions between biomacromolecules and pNIPAM hydrogels are significantly influenced by pH and temperature due to electrostatic interactions, seizing actions, and hydrophobic forces [367]. Therefore, small-molecule drugs and BAPPs exhibit distinct delivery mechanisms in thermosensitive hydrogels [368]. Lin et al. [368] developed a composite hydrogel in which calcium ion crosslinked sodium alginate served as the continuous phase. pNIPAM nanogels loaded with bFGF (with an LCST of 1 − 33 °C) and p(N-isopropylacrylamide-coacrylic acid) nanogels [p(NIPAM-co-AA), with an LCST of 2 − 40 °C] were incorporated as the dispersed phase along with diclofenac sodium. When the temperature is maintained between LCST1 and LCST2, diclofenac sodium can be released from swollen p(NIPAM-co-AA) nanogels, while bFGF remains immobilized within contracted pNIPAM nanogels. Conversely, when the temperature falls below LCST1, swelling of the pNIPAM nanogels facilitates the release of bFGF [368].

#### Light

Light-responsive delivery platforms hold significant promise for applications in tissue repair due to their precise control over exposure time and location, as well as their non-damaging nature. The photosensitive groups within light-responsive materials can undergo isomerization, cleavage, or dimerization when exposed to light of specific wavelengths. Three distinct wavelength ranges can be utilized to regulate the release of bioactive agents: ultraviolet light (200 − 400 nm), visible light (400 − 700 nm), and NIR light (700 − 1300 nm) [369].

Ultraviolet light provides sufficient energy to trigger the cleavage, isomerization, or rearrangement of photosensitive polymers, thereby regulating the release of BAPPs. Acrylic or coumarin bonds serve as photosensitive groups that can be cleaved under ultraviolet light irradiation, making them suitable for controlling drug release [370]. Azagarsamy et al. [371] covalently modified BMP-2 and BMP-7 with two distinct photocleavable azides (nitrobenzyl azide and coumarin azide, respectively) and conjugated these to PEG hydrogels via click chemistry. Nitrobenzyl azide exhibits high cleavage efficiency under 405 nm illumination for BMP-2 release, while coumarin azide demonstrates high cleavage efficiency under 365 nm illumination for BMP-7 release [371]. Additionally, azo bonds readily converted from the trans orientation to the cis orientation when irradiated with ultraviolet or visible light. Zhao et al. [372] constructed a supramolecular hydrogel based on host–guest interactions between trans-azobenzene and β-cyclodextrin groups coupled with HA chains, which could release EGF under simulated light to promote wound healing. The release rate of EGF from hydrogels treated with ultraviolet light was approximately 2 − 3 times greater than that from those treated with visible light, allowing for regulation of EGF through alternating irradiation with ultraviolet and visible light [372].

Visible light can be used to regulate the release of BAPPs when combined with photosensitive materials, thereby avoiding the tissue-damaging side effects associated with ultraviolet light. For example, Sieber et al. [373] incorporated VEGF-modified tetrapod zinc oxide (t-ZnO) particles into GelMA hydrogels to create a light-responsive delivery system. When illuminated by blue or green light, charges propagated to the surface of the t-ZnO particles and accumulated, resulting in altered surface polarity that facilitated VEGF release from the surface of ZnO particles due to electrostatic repulsion. Conversely, ultraviolet light does not trigger VEGF release; however, it remains effective for cross-linking GelMA hydrogels [373].

Compared with ultraviolet and visible light, NIR light can penetrate deeper into tissues. BAPPs and NIR-responsive particles are simultaneously introduced into polymers that exhibit thermal phase changes, enabling NIR-responsive release through the phase transitions of thermosensitive polymers induced by heat generated from the particles. Several natural thermosensitive polymers with phase change properties, such as gelatin [374], agarose [375], and chitosan [376], can be combined with NIR-responsive particles to regulate the release of BAPPs. For example, Wan et al. [376] used PDA-coated magnesium-calcium carbonate microspheres as NIR-responsive carriers for BMP-2 delivery. These BMP-2-loaded microspheres and aspirin were subsequently incorporated into a thermosensitive hydroxybutyl chitosan hydrogel to achieve a sequential release of aspirin and BMP-2. The results revealed that BMP-2 release in the NIR-treated group dramatically increased compared to the untreated group, demonstrating a sustained release model [376]. Additionally, certain synthetic thermosensitive polymers can be utilized to fabricate NIR-responsive delivery platforms in conjunction with NIR-responsive particles [377]. For example, Zhao et al. [378] embedded GO into a hydrogel composed of pNIPAM and GelMA, and GO was induced to undergo photothermal conversion under NIR irradiation, triggering the contraction of pNIPAM chains and resulting in the responsive release of VEGF. In another study, Liu et al. [379] designed a thermoresponsive hydrogel featuring an LCST above body temperature using n-isopropyl acrylamide along with carbon dots as photothermal converters. This hydrogel was then coated with a PTHrP-2-loaded mesoporous bioactive glass scaffold, allowing for controlled sustained and pulsatile release of PTHrP-2 via NIR irradiation [379].

Nonpolymer thermosensitive systems can also be integrated with NIR-responsive components to regulate the release of BAPPs in response to NIR. For instance, Che et al. [380] constructed a thermosensitive liposome for loading PTH 1-34, which was coated with PDA as a photothermal conversion component. The release of PTH 1-34 could be modulated through NIR-mediated heating, enhancing the extravasation of thermosensitive liposomes and facilitating the release of PTH 1-34. Upon temperature reduction, the liposomes rapidly reverted to a dense structure, thereby hindering further release of PTH 1-34 [380]. Additionally, Donsante et al. [381] incorporated NT-3 into a phase change material derived from a eutectic mixture of lauric acid and stearic acid, which was subsequently added between 2 layers of PCL electrospun fibers. The results showed that the photothermal effect induced by NIR stimulation led to the melting of fatty acids, thereby enabling controlled pulse release of NT-3 [381].

Light-responsive delivery platforms continue to encounter several challenges and limitations. The high energy of ultraviolet light can drive numerous photochemical and photoisomerization reactions, rendering many photosensitive groups less sensitive to visible light and NIR light, or capable of responding solely to ultraviolet light [382]. However, the use of ultraviolet light poses a greater risk of cellular and tissue damage. While NIR exhibits significantly enhanced tissue penetration compared to ultraviolet or visible light, it still falls short in meeting the requirements for deep noninvasive trigger-response release. Additionally, the biosafety of photoreactive materials and their products under light stimulation must also be thoroughly evaluated.

#### Magnetic field

Magnetic fields can deeply penetrate human tissue without harmful ionization effects [346]. Superparamagnetic nanoparticles, such as iron oxide (Fe_3_O_4_, Fe_2_O_3_), cobalt, and nickel oxide particles, are commonly utilized as delivery platforms that respond to magnetic fields. Among these, superparamagnetic iron oxide nanoparticles are particularly advantageous due to their favorable magnetic properties, excellent biocompatibility, and high specific surface area [269, 383]. Two types of magnetic fields can be applied to activate the magnetic response: alternating magnetic field (AMF) and stationary magnetic field (SMF).

AMFs are often used for heat generation to facilitate cargo release [361]. For example, researchers cross-linked TGF-β to the surface of SPION particles through carbodiimide and applied an external AMF to generate a thermal effect, thus achieving AMF-responsive release [384]. However, the release of TGF-β induced by AMF does not occur until the heating temperature reaches 55 °C, and substantial amounts of TGF-β are only released after heating the material to 80 °C [384]. Elevated temperatures often lead to the inactivation of BAPPs and potential tissue damage; therefore, temperature-sensitive delivery platforms can be integrated to respond to AMF-mediated heating for controlled release of BAPPs.

When SMFs are applied to magnetic nanoparticles, they induce the deformation of hydrogels, subsequently extruding BAPPs. For example, Kim et al. [385] developed an alginate iron hydrogel using heparin-grafted alginate and iron oxide nanoparticles, and the incorporated TGF-β1 could be extruded through hydrogel deformation induced by the movement of iron oxide nanoparticles following SMF application. Additionally, Tolouei et al. [386] fabricated a magnetically responsive biomaterial system comprising two compartments, an outer gelatin scaffold and an inner biphasic ferrogel. The upper layer of the biphasic ferrogel contains Fe_3_O_4_, while the lower layer consists of a porous and deformable region devoid of Fe_3_O_4_ [386]. Proinflammatory cytokines (MCP-1 and IFN-γ) loaded into the outer gelatin scaffold were initially released to promote proinflammatory macrophage phenotypes, whereas anti-inflammatory cytokines (IL-4 and IL-10) incorporated into the lower biphasic ferrogel could be precisely released via the deformation of inner compartment controlled by SMF to promote healing macrophage phenotypes [386]. Therefore, the release of BAPPs can be accurately regulated by the deformation of delivery platforms, such as hydrogels, induced by SMFs. Furthermore, release profiles correlated with the mechanical properties of the hydrogels, the number of magnetic nanoparticles, and the strength of the magnetic field.

The combination of AMF and SMFs can facilitate the spatiotemporal release of BAPPs. Specifically, an SMF can be initially applied to induce directional movement and accumulation of magnetic particles, followed by the application of an AMF to generate a magnetocaloric effect that promotes drug release. For example, Huang et al. [387] incorporated NGF into magnetic PLGA microcapsules containing iron oxide nanoparticles, subsequently introducing these microcapsules into a gelatin/silk hydrogel. The hydrogel underwent a reversible sol-to-gel phase transition due to gelatin dissolution above 20 °C, enabling the microcapsules to migrate within the aqueous phase of the hydrogel under SMF influence, resulting in a micropatterned hydrogel with corrugated topography. Micropatterned hydrogels can spatially and longitudinally guide neurite growth, while NGF release can be regulated through AMF-induced thermal effects on iron oxide nanoparticles [387].

In general, magnetic fields are regarded as safe, but the ions in the medium can generate eddy currents within the magnetic field, resulting in an increase in temperature at the site of action, which may damage healthy tissue. It is widely accepted that in biomedical applications, the product of magnetic field frequency and magnetic field strength should range from 1.8 × 10^9^ to 5 × 10^9^ Am^−1^ s^−1^ [361]. Another critical concern regarding magnetically responsive materials is the biosafety of implanted magnetic particles, whose in vivo toxicity is dose-dependent and time-dependent.

#### Electric field

Electric fields can be effectively utilized to control the release of BAPPs with high precision through the incorporation of conjugated polymers. Conjugated polymers, such as polypyrrole (PPy), polyaniline, and polythiophene (PTh), possess alternating single and double bonds that facilitate electron mobility along the polymer skeleton [388]. Upon application of electrical stimulation, drug-loaded conjugated polymers undergo redox reactions, resulting in changes to the polymer's charge that influence its conductivity, volume, and permeability, ultimately leading to the release of BAPPs [389]. For example, Miar et al. [390] deposited a conductive PPy coating onto the surface of polyvinylidene fluoride electrospun fibers doped with biotin at binding sites for bioactive proteins (bFGF and NGF), showing that t electrical stimulation could accelerate the release of these bioactive proteins. However, the loading capacity of delivery platforms based on conjugated polymers is limited when incorporating large molecular weight molecules [391]. Combining conjugated polymers with other biomaterials to fabricate delivery platforms such as hydrogels can overcome these limitations. For instance, Chen et al. [392] developed a conducting polymer hydrogel by integrating conductive poly(3,4-ethylenedioxythiophene) into GelMA hydrogel and employed BSA as a model for bioactive proteins to explore its release under electrical stimulation. The findings demonstrated that the hydrogel retained a high degree of charge transfer properties, and its release rate further increased approximately threefold after each electrical stimulation compared to the non-stimulation group [392].

Conductive particles can also be used for the electric field-responsive delivery of BAPPs, with common examples including metallic nanoparticles and carbon-based particles. For example, researchers developed an electrically responsive biohybrid composite film based on silk fibroin and rGO to deliver NGF, where rGO served as a conductive component [393]. NGF is released through passive diffusion in the absence of electrical stimulation, while its release is significantly improved by electrical stimulation [393]. In further conversion applications, it is necessary to consider the long-term performance stability of implanted conductive materials under physiological conditions, along with their biocompatibility and changes in conductivity. The safe voltage range is generally considered to be from − 0.6 V to + 0.8 V. Excessively high voltages may lead to tissue redox reactions, ROS production, and alterations in local pH levels [394]. Therefore, careful consideration should be given to the timing, frequency, and strength of electrical stimulation during in vivo application.

## Clinical potential of BAPPs

**Fig. 9:**
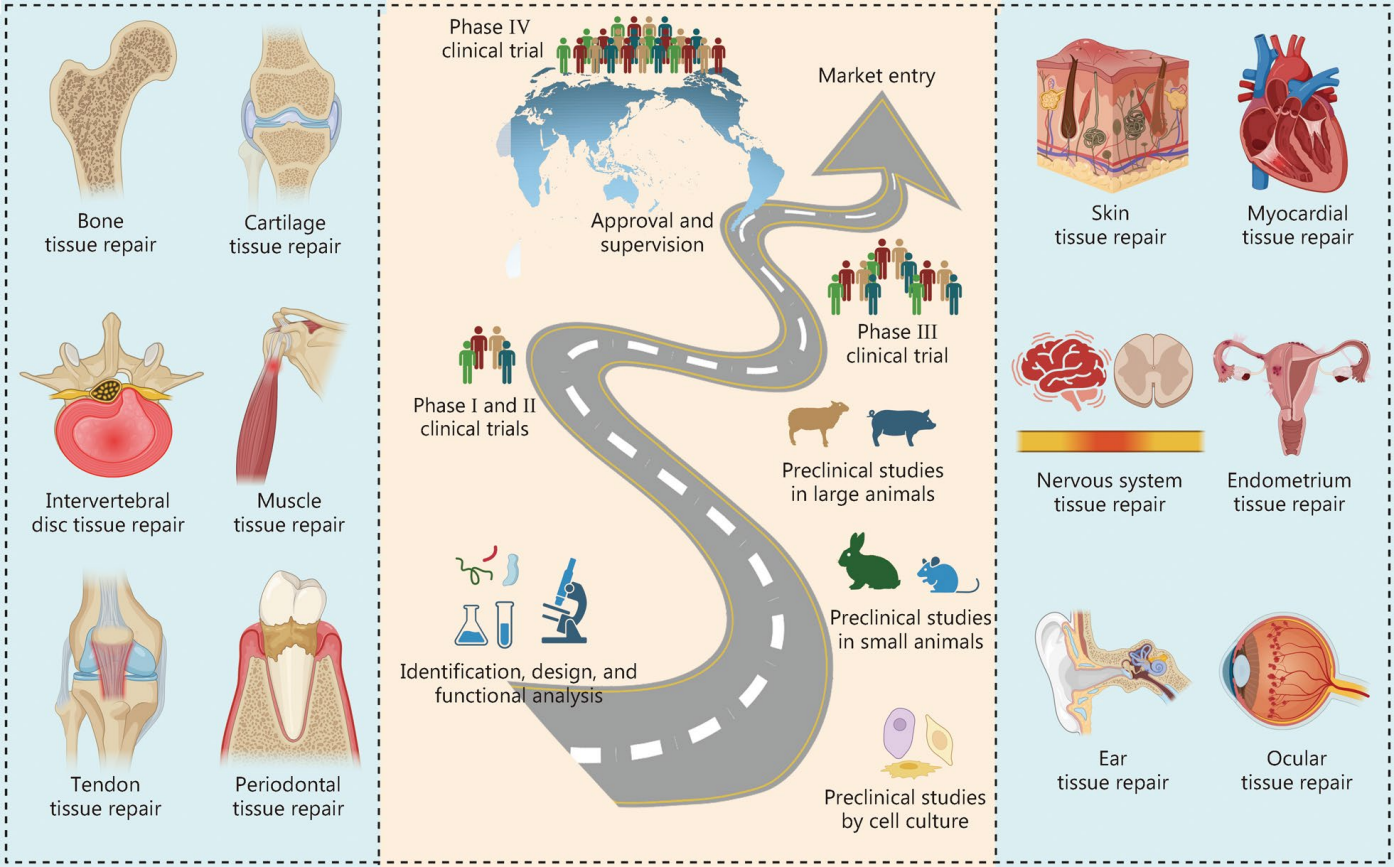
Clinical potential of bioactive peptides and proteins (BAPPs) for various tissue repair and critical steps for their clinical translation. BAPPs demonstrate significant potential for the clinical repair of a wide range of tissue, including bone, cartilage, intervertebral discs, muscle, tendons, periodontal tissue, skin, myocardial tissue, nervous system tissue, endometrium tissue, as well as ear and ocular tissue. In alignment with the critical steps necessary for their clinical translation, BAPPs are typically evaluated in preclinical studies through sequential cell culture followed by testing in small and large animals after the initial identification, design and functional analysis. Subsequently, they undergo evaluation via clinical trials from phase I to phase IV until receiving approval for market entry

BAPPs show great clinical potential due to their diverse functions and high therapeutic efficiency. The process of clinical translation typically involves several critical steps: basic scientific studies for identification, design and functional analysis; preclinical studies utilizing cell culture and animal models; clinical trials encompassing phase I pharmacology trials, phase II exploratory trials, phase III confirmatory trials and phase IV post-marketing trials; as well as institutional approvals and clinical supervision (Fig. 9). Currently, several BAPPs, including BMP-2, PDGF-BB, PTH 1–34 and abaloparatide, have received FDA approval for use in clinical tissue regeneration. Furthermore, numerous BAPPs are undergoing clinical trials and preclinical evaluations to assess their efficacy and safety for specific tissue repair. A summary of the representative BAPPs evaluated in clinical trials is presented in Table 2.

**Table 2:** Clinical trials of bioactive peptides and proteins (BAPPs) in tissue regeneration

Clinical trials	Conditions	Participants (age/gender)	BAPPs	Overcome	Phase	Study time
EudraCT 2008–000592-24	Newly formed bone after tibia distraction osteogenesis	16 (25 + /both)	PTH 1-34	Teriparatide treatment doubled bone regeneration when compared to no treatment	-	2009–2013
NCT01279187	Craniofacial osseous regeneration in bone	27 (30–85/both)	Teriparatide	-	Phase II	2011–2015
NCT00533793	Tibial shaft fractures	201 (adults, elder/both)	TGplPTH 1-34	-	Phase II	2007–2011
NCT04294004	Degenerative disc disease	50 (25–75/both)	TGplPTH 1-34	-	Phase II	2020–2025
NCT01033994	Knee osteoarthritis	192 (40 + /adults, elder/both)	Sprifermin	-	Phase I	2008–2010
NCT01066871	Acute cartilage injury of the knee	74 (18–45/both)	Sprifermin (AS902330)	-	Phase II	2010–2013
NCT01689337	Grade 3 degenerative joint disease of the knee	102 (18–70 adults, elder/both)	Sprifermin	-	Phase II	2011–2014
NCT05276011	Hip osteoarthritis	255 (18–80 adults, elder/both)	Sprifermin	-	Phase II	2024–2025
NCT02491281	Cartilage repair in primary osteoarthritis	28 (50–75 adults, elder/both)	LNA043	-	Phase I	2015–2018
NCT03275064	Articular cartilage lesions	142 (18–75 adults, elder/both)	LNA043	-	Phase II	2017–2022
NCT04864392	Knee osteoarthritis	581 (40–75 adults, elder/both)	LNA043	-	Phase II	2021–2027
NCT04814368	Knee osteoarthritis	24 (40–80 adults, elder/both)	LNA043	-	Phase II	2021–2024
NCT01124006	Early lumbar disc degeneration	24 (adults, elder/both)	RhGDF-5	-	Phase II	2010–2014
NCT01158924	Early lumbar disc degeneration	40 (adults, elder/both)	RhGDF-5	-	Phase I Phase II	2010–2016
NCT00813813	Early lumbar disc degeneration	32 (adults, elder/both)	RhGDF-5	-	Phase I Phase II	2008–2013
NCT01182337	Early lumbar disc degeneration	31 (adults, elder/both)	RhGDF-5	-	Phase I Phase II	2010–2014
NCT03708926	Lumbar disc degeneration	- (adults, elder/both)	Abaloparatide	-	Phase II	2021–2024
NCT01207908	Duchenne muscular dystrophy	44 (5 + adults, elder/both)	IGF-1	-	Phase I Phase II	2010–2013
NCT00577577	Myotonic dystrophy type 1	69 (21–65 adults, elder/both)	rhIGF-I rhIGFBP-3	-	Phase II	2007–2008
NCT00233519	Myotonic dystrophy type 1	17 (21–60 adults/both)	rhIGF-I rhIGFBP-3	-	Phase I Phase II	2005–2008
NCT01834989	Patellar tendinopathy	40 (18–50 adults/both)	IGF-1	-	Early phase I	2013–2020
NCT00936559	Rotator cuff repair	13 (25–75 adults, elder/both)	BMP-12	-	Phase I	2010–2012
NCT01122498	Gingival recession defects	30 (20–55 adults/both)	BMP-12	-	Phase I	2019–2021
NCT04375618	Gingival recession defects	30 (20–55 adults/both)	RhEGF	-	Phase I	2019–2021
NCT02236793	Chronic diabetic foot ulcer	252 (adults, elder/both)	PDGF-BB	-	Phase III	2014–2016
NCT01098357	Chronic diabetic foot ulcer	192 (18–75 adults, elder/both)	PDGF-BB	-	Phase I Phase II	2010–2011
NCT03037970	Chronic diabetic foot ulcer	40 (30–75 adults, elder/both)	RhPDGF-BB	-	Phase II	2017
NCT00740922	Chronic diabetic foot ulcer	563 (adults, elder/both)	RhPDGF-BB	-	Observational	1999–2001
NCT00812513	Healing wounds caused by third-degree thermal and electrical burns	120 (18–75 adults, elder/both)	R-Pdf/Gbb	-	Phase II	2011–2012
NCT00847925	Wound healing	103 (18–45 adults/male)	Juvista	-	Phase I Phase II	2001–2003
NCT00629811	Wound healing	78 (18–85 adults, elders/both)	Juvista	-	Phase II	2006–2007
NCT00847795	Wound healing	42 (60 + adults, elders/both)	Juvista	-	Phase I Phase II	2002–2003
NCT00430326	Wound healing	156 (18–85 adults, elders/both)	Juvista	-	Phase II	2006–2009
NCT00594581	Wound healing	71 (18–85 adults, elders/both)	Juvista	-	Phase II	2003–2005
NCT00432211	Scar appearance following scar revision surgery	60 (18–85 adults, elder/both)	Juvista	-	Phase I Phase II	2006–2012
NCT00117936	Coronary heart disease	150 (25–75 adults, elder/both)	FGF1–141	-	Phase II	2020–2023
NCT03686163	Acute ischemic stroke	106 (adults, elder/both)	NGF	-	Phase IV	2016–2020
NCT04041349	Cerebral small vessel disease	510 (50–80 adults, elder/both)	mNGF	-	Phase IV	2019–2021
NCT02490501	Traumatic spinal cord injury	27 (18–65 adults, elder/both)	FGF-1	-	Phase I Phase II	2015–2020
NCT06328166	Carpal tunnel syndrome	28 (18–80 adults, elder/both)	ES135 (RhFGF-1)	-	Not applicable	2024
NCT02193334	Acute spinal cord injury	45 (18–75 adults, elder/both)	KP-100IT (HGF)	-	Phase I Phase II	2014–2018
NCT04475224	Acute spinal cord Injury	25 (18–89 adults, elder/both)	KP-100IT (HGF)	-	Phase III	2020–2023
NCT04100655	Endometrial thickness in infertile women	96 (18–40 adults/both)	GM-CSF	-	Not applicable	2019–2020
NCT03023774	Recurrent Ivf/Icsi failure	20 (child, adults/female)	GM-CSF	-	Not applicable	2016
NCT01202643	Poor endometrial development during IVF	12 (21–45 adults/female)	GM-CSF	-	Phase I Phase II	2010–2013

*GM-CSF* granulocyte–macrophage colony-stimulating factor, *RhBMP* recombinant human bone morphometric proteins, *PTH 1–34* parathyroid hormone 1–34, *IGFBP-3* recombinant human IGF binding protein-3, *IGF-1* insulin-like growth factor-1, *HGF* hepatocyte growth factor, *RhPDGF-BB* recombinant human platelet-derived growth factor-BB, *FGF-1* fibroblast growth factor-1, *NGF* nerve growth Factor, *RhEGF* recombinant human epidermal growth factor, *RhGDF-5* recombinant human growth differentiation factor-5

### Bone tissue repair

While bone tissue possesses a self-healing capacity, critical defects or fractures resulting from trauma or surgical resection are difficult to repair autonomously, particularly in patients with metabolic diseases such as osteoporosis and diabetes. BAPPs exhibit significant potential to enhance the repair of bone tissue.

BMP-2 has potent osteoinductive properties and has been approved by the FDA for the induction of bone regeneration [395]. Clinically, BMP-2 is generally used for posterolateral fusion when combined with a collagen sponge or hydroxyapatite carrier [396]. A systematic review included 6 randomized clinical trials between 2002 and 2017, encompassing a total of 908 patients to compare the effects of BMP-2-loaded implants versus autologous iliac crest bone grafts in lumbar spine posterolateral fusion [397]. The results showed that healing rates at 24 months were 94% in the BMP-2 group compared to 83% in the iliac crest bone graft group, with the BMP-2 cohort demonstrating improvements in angiogenesis, operation duration, intraoperative blood loss, and length of hospital stay [397]. Additionally, BMP-2 has been employed for treating open tibia fractures [398], maxillary sinus fractures [399], and localized alveolar ridge augmentation [400]. A meta-analysis involving 10 studies with a total of 653 participants from 2007 to 2017 assessed the use of BMP-2 against iliac crest bone grafts for cleft lip and palate repair [401]. Overall analyses revealed that outcomes related to bone formation volume and filling percentage were comparable between BMP-2 and iliac crest bone grafts; however, complications risk and treatment costs associated with iliac crest grafting were higher [401]. In another meta-analysis comprising 6 randomized controlled trials from January 1990 to February 2015, researchers evaluated the efficacy of conventional bone grafting relative to that of BMP-2 during maxillary sinus floor elevation, reporting similar clinical and histological outcomes [402]. Nevertheless, challenges associated with BMP-2 include its short half-life, complex production equipment and preparation process, as well as supraphysiological doses, which may lead to side effects such as inflammation, pain, heterotopic ossification, or even osteolysis [403]. Consequently, BMP-2 mimetic peptides have been developed as potential substitutes for bone regeneration due to their advantages including low cost and feasibility for large-scale synthesis. For example, a BMP-2 mimetic peptide known as P20 (KIPKASSVPTELSAISTLYL) was derived from the knuckle antigenic epitope, which represents the core functional region essential for osteogenesis [81]. To enhance its osteogenic activity and affinity for calcium phosphorus materials, P20 was modified by incorporating a serine at one end and three aspartic acids at the opposite end, resulting in a novel BMP-2 mimetic peptide designated as P24 (SpKIPKASSVPTELSAISTLYLDDD) [82].

BMP-7, also referred to as osteogenic protein-1 (OP-1), is another bioactive peptide from the BMP family that has received FDA approval for clinical applications in bone regeneration. In clinical trials, BMP-7 has been utilized for posterolateral fusion, with results indicating that patients treated with OP-1 exhibited comparable efficacy and few implant-related adverse effects compared to those receiving autografts [404, 405]. Additionally, BMP-7 has been employed in the treatment of tibial or femoral nonunion in clinical settings, demonstrating its effectiveness in enhancing healing rates for nonunions [406, 407]. Furthermore, a BMP-7 mimetic peptide (GQGFSYPYKAVFSTQ) has been developed through preclinical studies aimed at facilitating bone tissue regeneration while addressing the limitations associated with macromolecular forms of BMP-7 [408].

PTH is a bioactive peptide hormone composed of 84 amino acids, synthesized and secreted by the parathyroid gland, playing a crucial role in bone remodeling. PTH 1–34, commonly known as teriparatide in the clinic, is the first 34 amino acids at the N-terminal end of PTH and exhibits similar osteogenic effects [88]. The FDA has approved PTH 1–34 for the treatment of osteoporosis via intermittent subcutaneous injection. Intermittent administration of PTH 1–34 has also been used to promote bone tissue regeneration for bone defect healing. For example, in a clinical trial (EudraCT 2008–000592-24), intermittent PTH 1–34 was administered for patients undergoing bone segment transport due to tibial defects following infection, and the results showed that the treatment group receiving PTH 1–34 improved BMD compared with the control group [409]. In another clinical trial (NCT01279187), intermittent administration of PTH 1–34 was utilized for craniofacial osseous regeneration. However, topical application of PTH 1–34 may lead to overactivation of osteoclasts, potentially resulting in compromised bone healing outcomes. Sustained release of PTH 1–34 could mitigate this challenge. A transglutaminase substrate along with a plasmin-clearable sequence was incorporated into PTH 1–34 to construct a prodrug named TGplPTH 1–34 [379]. The transglutaminase substrate facilitates conjugation between PTH 1–34 and fibrin through factor XIIIa activity while allowing endogenous plasmin-mediated release via its clearable sequence [379]. Implants containing TGplPTH 1–34 have been evaluated in a clinical trial (NCT00533793) aimed at treating acute open tibial shaft fracture, and it was found that this prodrug effectively promotes bone regeneration [410]. Furthermore, TGplPTH 1–34 is currently under investigation for single-level transforaminal lumbar interbody fusion (NCT04294004). In preclinical studies, distinctive peptides related to PTH, including PTHrP–1 [411], PTHrP–2 [412], and P1R16 [413], have been developed for topical applications in bone regeneration and exhibit promising potential for further clinical use.

PTH-related proteins are naturally occurring bioactive proteins that facilitate bone regeneration via paracrine or autocrine secretion [89, 90]. PTHrP 1–37 represents the first 37 amino acids at the N-terminus of PTH-related proteins and exhibits bioactive effects on bone tissue regeneration [89]. Abaloparatide is a PTHrP 1–34 analog with 76% similarity to PTHrP 1–34 and 41% similarity to PTH 1–34, enhancing osteogenesis while reducing osteoclastogenesis compared to both PTHrP 1–37 and PTH 1–34. Since its approval by the FDA in 2017 for treating osteoporosis in postmenopausal women [92, 414], abaloparatide has been utilized in recent clinical trials aimed at inducing bone regeneration for spinal fusions (NCT03841058), odontoid fractures, supracondylar distal femur fractures (NCT04626141), and pelvic fractures (NCT04249232). Furthermore, topical administration of abaloparatide also shows great potential for promoting the healing of bone defects.

PDGF-BB is a multifunctional bioactive protein that promotes bone tissue regeneration through mechanisms of osteogenesis and angiogenesis. This systematic review included 3 randomized controlled trials involving 634 patients up to July 2015, aimed at evaluating the safety and efficacy of rhPDGF-BB compared to autogenous bone grafts for foot and ankle fusion [415]. No significant differences were observed in radiological efficacy or clinical outcomes between the two groups, with rhPDGF-BB demonstrating comparable safety profiles to autogenous bone graft [415]. Moreover, the rhPDGF-BB group can mitigate several issues associated with autogenous bone grafts, such as blood loss, scarring, extra surgical time, and donor pain [415]. In clinical trials, PDGF-BB has been combined with a β-TCP matrix (NCT00583375) [416] or a β-TCP-collagen matrix (NCT01008891) [417] for foot and ankle fusion. Additionally, PDGF-BB is being incorporated into a resorbable barrier membrane along with bone particulate allogenic graft for the treatment of deficient bone ridges in an ongoing clinical trial (NCT04954664).

P-15 is a bioactive peptide that mimics the cell-binding domain of type I collagen, thereby enhancing the attachment and activity of osteoblasts [418]. In a clinical trial (NCT00310440), P-15 was adsorbed onto an organic bone mineral and subsequently introduced to an inert biocompatible hydrogel to fabricate an i-Factor™ bone graft for single-level anterior cervical discectomy and fusion. The results revealed that bone regeneration induced by i-Factor™ was similar to that induced by autografts two years post-surgery [419]. This systematic analysis included 5 studies including 388 patients up to August 2023, assessing overall outcomes following lumbar surgery using the iFactor. Findings showed that patients treated with the i-Factor during lumbar surgery experienced outcomes similar to those receiving alternative transplant methods but demonstrated significantly faster fusion. Additionally, the i-Factor™ has received FDA approval for use in single-level anterior cervical discectomy and fusion from C3 to C7 [419]. A subsequent study confirmed both the efficacy and safety of this procedure after 6 years of follow-up [420].

In addition to the aforementioned BAPPs that have been evaluated in the clinic, several agents have undergone testing in preclinical studies. For example, OGP (ALKRQGRTLYGFGG) is the naturally occurring peptide derived from human serum that promotes osteogenesis, and its C-terminal pentapeptide named OGP (10–14) represents the physiologically active form of OGP [93, 421]. Both OGP and OPG (10–14) have been utilized in preclinical investigations for bone regeneration [422], demonstrating significant potential for future clinical applications. Additionally, NELL-1 is a bone-inducing protein that promotes bone differentiation by mediating key downstream effects of the osteogenic regulator RUNX 2 [423]. Moreover, NELL-1 has been shown to enhance BMP-2-induced bone formation while inhibiting BMP-2-induced lipogenesis via the Wnt signaling pathway [423]. Reports indicate that NELL-1 exhibits bone-inducing effects in vivo across various preclinical models. Collectively, these findings suggest that NELL-1 holds considerable promise for broad clinical application.

In recent years, there has been a growing emphasis on multifunctional materials for bone tissue repair. For example, sensory nerve fibers are predominantly located in metabolically active regions and play a direct role in bone formation through the secretion of neuropeptides such as calcitonin gene-related peptide, substance P, and semaphorin 3A. The localized delivery of these neuropeptides by vectors or the stimulation of their production to promote bone regeneration has shown clinical potential in animal experiments [424]. Additionally, antimicrobial peptides (AMPs) exhibit significant promise for bone tissue repair due to their bactericidal, antibiofilm, immunomodulatory, and regenerative properties [425]. Furthermore, the immune microenvironment at the site of bone injury is important for bone tissue regeneration. Macrophage polarization toward the M2 phenotype under stimuli can facilitate this process by promoting stem cell differentiation and extracellular matrix remodeling [426]. Immunomodulatory cytokines such as IL-4 combined with nanostructured scaffolds have been employed in osteogenic studies involving rats [426].

### Cartilage tissue repair

Cartilage regeneration is commonly related to the repair of articular cartilage, a critical component of joints that provides cushioning and reduces friction. However, the intrinsic capacity for articular cartilage repair is limited due to the absence of essential blood vessels and the restricted proliferative capacity of chondrocytes. Following cartilage injuries, there is often a progression to osteoarthritis, which imposes a significant health burden on individuals and society.

The TGF-β superfamily is widely recognized for its role in cartilage tissue repair [97]. TGF-β1 plays a vital role in inducing chondrocyte differentiation and regulating ECM synthesis [427]. In a clinical trial (NCT01221441), human chondrocytes expressing TGF-β1 (TG-C) were utilized to treat patients with knee osteoarthritis, demonstrating that intra-articular administration of TG-C improved the function of the knee joint and reduced pain severity [428]. Furthermore, TG-C is currently being investigated in ongoing clinical trials for patients with Kellgren and Lawrence Grade 2 or 3 Osteoarthritis (NCT03291470) and symptomatic early hip osteoarthritis (NCT05276011). However, existing clinical studies have primarily focused on the direct application of TGF-β1 for cartilage tissue repair. In preclinical studies, sustained release of TGF-β1 has been used to promote cartilage regeneration [429]. To overcome the challenges associated with the poor stability and short half-life of TGF-β1, distinctive TGF-β1 mimetic peptides such as LIANAK [98] and YYVGRKPK [97] have been developed for cartilage tissue repair. Additionally, intra-articular BMP-7 has also been evaluated in clinical trials involving patients with osteoarthritis (NCT01111045 and NCT01133613). In a phase I clinical trial (NCT00456157), patients treated with 0.1 mg and 0.3 mg doses of BMP-7 exhibited greater symptomatic improvement compared with those receiving placebo, and no ectopic bone formation or dose-limiting toxicity was observed among patients treated with BMP-7 [107].

FGF-18, commonly referred to as sprifermin, is a promising candidate for cartilage tissue repair and has been widely used in this context. In a clinical trial (NCT01919164), intra-articular administration of sprifermin every 6 or 12 months resulted in significant improvements in total femorotibial cartilage thickness after 2 years compared to placebo [430]. A subsequent study further confirmed that intra-articular sprifermin not only reduces cartilage loss but also increases cartilage gain [431]. When administered via intra-articular injection to patients with knee osteoarthritis, the 5-year efficacy and safety results showed that sprifermin effectively modified cartilage, leading to reductions in patient pain and the rate of knee replacement surgeries [431]. In another clinical trial (NCT01033994), sprifermin was investigated for its effects on patients with knee osteoarthritis who did not require surgical intervention. The results confirmed its efficacy through structural secondary endpoints alongside high safety [432, 433]. It was also shown that sprifermin increases cartilage thickness while simultaneously reducing cartilage loss [434]. Moreover, intra-articular administration of sprifermin has been applied for patients with acute cartilage injury of the knee (NCT01066871), where treatment outcomes revealed reductions in both defect volume and cartilage defect thickness. Another clinical trial (NCT01689337) also evaluated the efficacy and safety of sprifermin in patients following microfracture surgery for knee cartilage injury; however, the study was terminated due to low recruitment.

LNA043 is a derivative of the C-terminal portion of angiopoietin-like 3, recognized as a potent inducer of chondrogenesis for cartilage tissue repair. In a first-in-human clinical trial (NCT02491281), LNA043 was administered via intra-articular injection before total knee replacement, and results showed that LNA043 could reverse the transcriptome signature associated with osteoarthritis by promoting the expression of hyaline cartilage matrix components and anabolic signaling pathways [435]. Moreover, LNA043 is currently being investigated in clinical trials for the treatment of articular cartilage lesions (NCT03275064) and knee osteoarthritis (NCT04864392, NCT04814368).

In preclinical studies, some BAPPs have been evaluated for their efficacy in cartilage repair. For example, recent investigation has focused on the endogenous recruitment of stem cells for cartilage regeneration, with BMHP1 and BMHP2 being conjugated to RADA16 to fabricate functional hydrogels [73]. Additionally, the inflammatory microenvironment is an important factor influencing the regeneration of cartilage tissue, which can inhibit the differentiation of MSCs such as chondrocytes, leading to chondrocyte death and hypertrophy, extracellular matrix decomposition, and even promoting the progression of osteoarthritis [436]. Therefore, modulating the local inflammatory environment is another strategy to enhance cartilage regeneration. Researchers have explored the effects of local delivery of IL-4 on cartilage regeneration in rat models [437]. Sneaking ligand construct (SLC1) is a ligand fusion protein that effectively inhibits the NF-κB signaling pathway and transcription of proinflammatory cytokines after selective endocytosis [438]. Although not yet utilized in clinical trials, SLC1 shows promising applications in cartilage regeneration.

### Intervertebral disc tissue repair

The intervertebral disc is a complex and specialized joint comprising the nucleus pulposus in the central region, the peripheral annulus fibrosus, and cartilaginous endplates on the upper and lower sides. Regeneration of the intervertebral disc is generally difficult due to its avascular structure. Recent studies have demonstrated that degenerated disc tissues can recruit endogenous stem cells by releasing chemotactic cytokines such as IGF-1, TGF-β, SDF-1, and CCL-5 [99–101]. Additionally, GDF-5 plays a crucial role in intervertebral disc degeneration by inhibiting apoptosis of nucleus pulposus cells, promoting the synthesis of key ECM components, and downregulating the expression of proinflammatory factors, thus alleviating intervertebral disc degeneration [439]. In clinical trials (NCT01124006, NCT01158924, NCT00813813, and NCT01182337), intradiscal GDF-5 has been used to evaluate the safety, tolerability, and preliminary effectiveness in patients with early-stage lumbar disc degeneration. Results indicated that GDF-5 provided effective treatment outcomes for function improvement and pain relief while demonstrating high safety. Furthermore, PTH, PTH-related proteins, and their derivatives identified in preclinical studies are promising candidates for attenuating intervertebral disc degeneration [440]. In a clinical trial (NCT03708926), daily administration of abaloparatide via injection improved pain levels, functional capacity, and overall disc health in subjects experiencing low back pain secondary to lumbar disc degeneration. Preclinical investigations have also designed a bionic peptide, octadecadienoic acid (CIS-9,12)-FFVLK-FKPHFPKSYTKICQ (OAFF), for treating disc degeneration [441]. Subsequent animal experiments confirmed that OAFF could inhibit CXCL8 to maintain resting NPSCs through the PI3K-Akt-mammalian target of rapamycin (mTOR) signaling pathway, thereby inhibiting disease progression in a rat tail disc puncture model [441].

### Muscle tissue repair

Muscle tissue repair is correlated with muscular dystrophy or defects resulting from damage or excision, primarily involving satellite cells (or skeletal muscle stem cells). IGF-1 is a potent bioactive protein that induces the proliferation and differentiation of satellite cells, demonstrating significant potential for clinical applications in muscle tissue repair. IGF-1 has been investigated in clinical trials for the treatment of Duchenne muscular dystrophy (NCT01207908) and myotonic dystrophy type 1 (NCT00577577 and NCT00233519). In one published clinical trial (NCT00233519) involving 15 participants, SomatoKine (Iplex), a combination of IGF-1 and IGF-1 binding protein-3, was administered to patients with myotonic dystrophy type 1, indicating that SomatoKine could enhance lean body muscle mass and metabolism without significantly improving muscle strength or function [442]. Further randomized controlled clinical trials are needed to assess the safety and efficacy of these treatments. In preclinical studies, IGF-1 has been applied for repairing muscle loss or defects through topical sustained release, showing promising potential for further clinical applications [443].

### Tendon tissue repair

The tendon is a uniquely mechanosensitive tissue that transmits forces from muscle to bone, with common injuries including rotator cuff injuries, finger flexor tendon injuries, patellar tendon injuries, and Achilles tendon injuries [102]. In a clinical trial (NCT01834989), IGF-1 was locally infused to improve the tendon structure in patients with patellar tendinopathy, but the overall results indicated no therapeutic benefit for tendon healing associated with local IGF-1 injection [444]. Further clinical trials are warranted, and delivery strategies should be optimized. Additionally, BMP-12 serves as another bioactive protein for tendon tissue repair, and it was loaded into absorbable collagen sponges as adjuvant therapy for patients undergoing open rotator cuff repair in clinical trials (NCT00936559 and NCT01122498) [445]. Results demonstrated that the addition of BMP-12 to absorbable collagen sponges was both feasible and safe while promoting functional recovery [445]. Beyond these growth factors, an extracellular matrix-associated protein periostin expressed in tendon but not limited to bone contributes to the repair of tendon tissue. A preclinical study revealed that recombinant POSTN promotes the stemness, proliferation, and tenogenic differentiation of tendon stem/progenitor cells while facilitating the healing of total excision Achilles tendon defects when delivered via a bionic scaffold [446].

### Periodontal tissue repair

The repair of periodontal tissue encompasses a variety of complex structures and multiple tissue types, including the formation of alveolar bone, osteoid regeneration on exposed root surfaces, and the orderly insertion of Sharpey’s fibers into both intrinsic alveolar bone and newly formed bone. Each tissue exhibits distinct structural characteristics and functions while interacting in a coordinated manner, thereby complicating the reconstruction of the physiologic periodontal structure.

PDGF-BB, FGF-2, and BMP-2 have been extensively utilized in clinical settings for periodontal tissue repair [105, 106, 447]. PDGF-BB has received FDA approval for this purpose. A systematic review encompassing 63 human clinical studies up to June 2019 demonstrated that PDGF-BB is advantageous for treating periodontal defects and gingival recession when used in conjunction with bone allografts, xenografts, or β-TCP [105]. Furthermore, a systematic review of 34 clinical trials conducted from January 2000 to November 2022 evaluated the clinical potential of FGF-2 in promoting periodontal regeneration through radiographic bone filling, secondary probing pocket depth, and probing attachment levels. The findings indicated that FGF-2 enhances radiographic bone filling, particularly when combined with bone substitutes, although it did not yield additional benefits regarding secondary probing pocket depth or probing attachment levels [106]. For BMP-2, a systematic review including 2 clinical studies with a total of 48 samples from January 1980 to December 2017 investigated its potential for periodontitis patients with intrabony defects by analyzing radiographic bone fill, gain in clinical attachment level, and reduction in pocket depth [447]. Compared to open-flap debridement procedures, BMP-2 promoted defect healing while reducing both the gain in clinical attachment levels and reduced pocket depth, making it a promising alternative to traditional grafts [447]. However, after 6 months compared to platelet-rich fibrin treatment outcomes, only radiographic bone filling showed significant improvement following BMP-2 application [447]. In addition to these bioactive proteins mentioned above, EGF is currently undergoing clinical assessment for patients suffering from gingival recession defects (NCT04375618).

The cell-binding peptide P-15 has also been used for periodontal tissue repair. A systematic review and meta-analysis included clinical trials investigating the application of an organic bovine-derived hydroxyapatite matrix combined with P-15 for the healing of periodontal defects up to December 2019. The findings indicated that, compared to open flap debridement, the P-15-loaded organic bovine-derived hydroxyapatite matrix significantly enhanced clinical attachment levels while reducing the probing depth and gingival recession.

Certain odontoblast-specific proteins, such as dentin-derived growth factor, cementum attachment protein, and cementum osteoprotegerin-1, have been shown to promote neodentin and bone formation in damaged periodontal tissues [448]. Although they have not yet undergone clinical trials, they possess the ability to induce a mineralized extracellular matrix similar to that of dental bone, showing their potential for further clinical applications. Moreover, the inflammatory response surrounding dental implants is a prevalent factor that impedes repair. Given the broad-spectrum activity of AMPs and their low risk for inducing bacterial resistance, AMPs present promising prospects for application in periodontal tissue repair [449]. Numerous animal experiments have verified the effect of AMP coatings on dental implant surfaces in enhancing periodontal tissue repair. However, considerations regarding the reliability of AMPs, their long-term effects on microorganisms in the body, as well as biocompatibility and toxicity must also be addressed [450].

### Skin tissue repair

Skin tissue repair is crucial for the restoration of barrier function and can be categorized into 4 stages: inflammation, angiogenesis, extracellular matrix synthesis, and tissue remodeling. ECM synthesis, re-epithelialization, and angiogenesis collectively contribute to skin tissue repair. EGF, FGF, PDGF, and GM-CSF are the three bioactive proteins most frequently utilized in clinical trials for skin tissue repair [107, 108]. In a systematic review and meta-analysis, 229 papers with a total of 281 studies were conducted to evaluate the efficacy and safety of EGF, FGF, and GM-CSF in treating acute skin wounds. The findings indicated that the administration of these bioactive proteins significantly shortens the healing time for various wound types, including superficial burns, deep burns, trauma, and surgical wounds [451]. Additionally, treatment with these bioactive proteins resulted in decreased scar scores with high safety [451]. Another systematic review and meta-analysis that included 13 studies involving 1924 participants across 2130 wounds further confirmed that EGF, FGF, and GM-CSF could promote healing in partial-thickness burns [452]. In terms of chronic diabetic foot ulcer management, a systematic review comprising 26 randomized controlled trials revealed that FGF exhibited substantial effectiveness in enhancing healing outcomes for diabetic ulcers [453].

PDGF-BB is another potent bioactive protein utilized in skin tissue repair. In a phase III clinical trial, PDGF-BB was administered to patients with chronic neuropathic diabetic ulcers of the lower extremities, and the results showed that 100 μg/g PDGF-BB significantly enhanced complete wound healing while reducing healing time [108]. Additionally, a combined analysis that included 4 randomized studies further confirmed the efficacy of PDGF-BB in treating chronic full-thickness diabetic ulcers of the lower extremities [454]. In another phase clinical trial, PDGF-BB exhibited effective outcomes in managing pressure ulcers [455]. Moreover, PDGF-BB has been evaluated in clinical trials for diabetic foot ulcer treatment (NCT02236793, NCT01098357, NCT03037970, and NCT00740922) as well as for third-degree thermal and electrical burns (NCT00812513). TGF-β3 is also a potent protein for skin tissue regeneration. Several randomized phase I/II clinical trials (NCT00847925, NCT00847795, NCT00629811, NCT00432211, NCT00430326, and NCT00594581) investigated Juvista (Avotermin), known as TGF-β3, for treating scarring following skin injury. The results showed that this treatment improved scar appearance compared to placebo [456–458]. However, Juvista did not meet its primary endpoints in phase III clinical trials [459].

With the emergence of drug-resistant microorganisms and the misuse of antibiotics, AMPs present significant potential as therapeutic agents for combating bacterial infections and promoting skin regeneration. The LL37 peptide is an endogenous antimicrobial peptide found in human skin, exhibiting antimicrobial, angiogenic, and immunomodulatory properties [460]. In a phase II clinical trial focused on treating venous ulcers in the lower extremities, LL37 demonstrated its capacity to enhance wound healing compared to placebo at specific concentrations, with no adverse effects reported [460]. Furthermore, a clinical trial (NCT04098562) is currently underway to investigate the efficacy of LL-37 in patients with diabetic foot ulcers. Additionally, various AMPs, such as melittin [461], P5 S9 K [462], and Chol-37 [463], have been shown to facilitate wound healing in murine models.

### Myocardial tissue repair

Myocardial tissue repair primarily refers to the restoration of ischemic heart disease, which is correlated with insufficient oxygen supply and detrimental excessive fibrosis. The positive induction of ECs is dedicated to the formation of new blood capillaries to alleviate hypoxia and prevent cardiomyocyte death. Current trials have mainly focused on angiogenesis therapy. Increased levels of programmed cell death 5 (PDCD5) subsequently ameliorated progressive fibrosis and cardiac dysfunction by inhibiting histone deacetylase 3 (HDAC3), suggesting that PDCD5 serves as an endogenous negative feedback regulator in progressive cardiac fibrosis [109]. FGF-1 is currently under investigation in a clinical trial (NCT00117936) for patients with coronary heart disease. In preclinical studies, sustained release of VEGF, PDGF, HGF, and IGF-1 from hydrogels has demonstrated promising potential for myocardial tissue repair, indicating their applicability in clinical settings [4]. Additionally, mitochondrial-derived peptide-c (MOTS-c) has been shown to reverse mitochondrial dysfunction and reduce oxidative stress, thereby alleviating metabolic reprogramming, decreasing beta-oxidation, and increasing glycolytic flux influenced by abnormal metabolism during myocardial infarction [464]. Local delivery of MOTS-c has been found to promote cardiac remodeling in rat models of myocardial infarction [464]. Furthermore, death-domain associated protein 6 (DAXX) regulates Fas protein-dependent apoptotic signaling during myocardial ischemia–reperfusion injury [465]. The interference peptide Tat-DAXXp, derived from DAXX coupled with a cell-penetrating peptide and a short transmembrane peptide, has exhibited anti-apoptotic effects and improved recovery of myocardial function in mouse models of myocardial infarction [465].

### Nervous system tissue repair

Nervous system tissue repair in the brain, spinal cord, and peripheral nerves mainly involves neurons, neuroglial cells, and NSCs. The survival and functionality of these cells, along with the neurogenesis of NSCs, contribute to nervous system tissue repair [102]. Brain tissue repair is typically associated with acute diseases such as stroke and traumatic injury or chronic diseases such as Alzheimer’s disease. Conversely, the repair processes for the spinal cord and peripheral nerves are linked to traumatic injuries resulting from external forces.

Neurotrophins, including NGF, BDNF, and NT-3, are essential bioactive proteins involved in neural development and regeneration. NGF has been used in clinical trials for brain tissue repair. In a clinical trial (NCT03686163), intranasal NGF was administered to treat acute ischemic stroke, with normal saline serving as a control. Treatment commenced between 24 and 72 h post-stroke and continued for 2 weeks. Another clinical trial (NCT04041349) randomized 510 patients with cognitive impairment due to cerebral small-vessel disease into two groups: the standard treatment group and the NGF-treated group. Researchers have investigated the clinical efficacy of mouse NGF on cognitive deficits by evaluating patient imaging performance. Additionally, NGF has been employed via intracerebroventricular infusion for patients with Alzheimer’s disease; however, notable side effects were observed despite these patients exhibiting normal EEG patterns, upregulated nicotinic receptor expression, and increased glucose metabolism [466, 467]. Therefore, optimized strategies for delivering NGF across the blood–brain barrier should be developed and tested in future clinical trials. Moreover, NGF can facilitate peripheral nerve repair. A systematic review encompassing 41 clinical studies revealed that NGF therapy effectively improved outcomes related to peripheral nerve injury while maintaining limited adverse reactions [468]. Although BDNF is not currently used directly in clinical trials for nervous system tissue repair, other pharmacological drugs such as memantine, donepezil, and atorvastatin, are being investigated to enhance BDNF levels for stroke treatment [469]. Preclinical studies have also led to the development of NGF mimetic peptides and BDNF mimetic peptides for nervous system tissue repair, showing promising potential for further clinical applications. Furthermore, localized release of NT-3 has been explored in animal models, and the results demonstrated effective neuroregeneration [470, 471] and synaptic restoration [113, 472].

In addition to neurotrophins, other bioactive proteins are also available for nerve tissue repair. FGF-1 and FGF-2 are versatile proteins that promote both neurogenesis and angiogenesis. Currently, FGF-1 is being evaluated in a clinical trial (NCT02490501) aimed at repairing spinal cord injuries. This trial seeks to assess the safety, tolerability, and efficacy of heparin-activated FGF1 delivered via a biodegradable device in patients with complete traumatic spinal cord injury. The intervention group received SC0806, a biodegradable device containing heparin-activated FGF1 along with nerve implants, combined with rehabilitation, while the control group underwent rehabilitation alone. Additionally, FGF1 has been utilized in another clinical trial (NCT06328166) to demonstrate the therapeutic efficacy and safety of ES135 (a recombinant human FGF1) for patients suffering from carpal tunnel syndrome. Participants diagnosed with mild to moderate CTS were randomly assigned to receive either ES135 or a placebo. Injections were administered between the median nerve and transcarpal ligament under ultrasound guidance. Outcomes were assessed using the Boston Carpal Tunnel Syndrome Questionnaire, electrophysiological studies, and measurement of the cross-sectional area of the median nerve at 1-, 2-, 3-, 4-, and 6-months post-injection. Moreover, HGF is currently under investigation for its potential role in repairing acute spinal cord injuries through ongoing clinical trials (NCT02193334 and NCT04475224).

Several bioactive peptides exhibit promising potential for nervous system tissue repair. For example, neuropeptides such as CGRP play a critical role in the repair of peripheral nerves due to their bioactivity in Schwann cells. Following injury, CGRP interacts with its receptors CGRP receptor (CRLR) and receptor modifying protein (RAMP-1), which are expressed on Schwann cells, thereby activating the downstream PI3K-Akt pathway and the RAS-Erk1/2 pathway to promote cell proliferation and dedifferentiation essential for peripheral nerve regeneration [111]. Additionally, chondroitin sulfate proteoglycans (CSPGs) are highly expressed at sites of nerve injury, and scar tissue rich in CSPGs inhibits axonal germination and growth [112]. In rat models of traumatic spinal cord injury, membrane-permeable intracellular σ peptide and intracellular LAR peptide have been shown to specifically inhibit CSPG expression, promoting both axon regeneration and functional recovery [112]. Moreover, Ily lys-val-Ala-Val (IKVAV), one of the main bioactive peptides found in laminin, facilitates the differentiation of neural precursor cells into neurons. IKVAV has been combined with other supramolecules to construct active peptide scaffolds, and its ability to promote nerve regeneration was demonstrated in a rat model of traumatic spinal cord injury [113, 473].

### Endometrial tissue repair

The endometrium is the only human tissue that undergoes periodic breakdown and regeneration, necessitating a transition of tissue cells between mesenchymal and epithelial phenotypes, a process known as mesenchymal-epithelial transition and epithelial-mesenchymal transition [470, 471]. Repair and regeneration of the endometrium are essential for reestablishing anatomical conditions conducive to embryo implantation and maintaining pregnancy following endometrial injury. Granulocyte colony-stimulating factor (G-CSF) is a multifunctional cytokine with immunomodulatory, vasoprotective, and angiogenesis-promoting properties. It has been evaluated for its efficacy in endometrial repair through several clinical trials (NCT04100655, NCT03023774, NCT01202643, IRCT201012272576N4 [474], and ChiCTR-IPR-17011242 [475]). A systematic review encompassing 10 studies involving patients with thin endometrium indicated that treatment with G-CSF via transvaginal perfusion significantly increased the endometrial thickness and improved gestation rates [476]. Moreover, KGF plays multiple important roles in regulating epithelial cell migration and proliferation as well as mediating epithelial-mesenchymal interactions. KGF treatment was shown to enhance the proliferation of both endometrial glandular epithelial cells and luminal epithelial cells during the healing process of endometrial injury in a preclinical study [114]. Therefore, KGF demonstrates promising potential for clinical applications in endometrial tissue repair. Additionally, SDF-1α can recruit multiple MSCs to promote tissue repair. E7 peptide-modified scaffolds have been developed to selectively capture MSCs [477]. A previous study has confirmed the feasibility of using collagen scaffolds co-modified with SDF-1 and E7 in rat models of endometrial injury [477].

### Ear tissue repair

The tympanic membrane is a common site of clinical ear disease, and growth factors have been used in tympanic myringoplasty to facilitate the healing of chronic non-healing tympanic membrane perforations. FGF-2, bFGF, EGF, and other growth factors can promote the regeneration of epithelial cells and fibroblasts, leading to numerous clinical applications for the tympanic membrane. A meta-analysis that included 14 studies involving a total of 1072 patients from 2003 to 2018 analyzed the therapeutic effects of bFGF on the tympanic membrane [478]. The findings indicated that bFGF significantly improves the rate of tympanic membrane regeneration and reduces healing time. However, there is no evidence supporting a significant effect on hearing improvement [478]. In another systematic review assessing FGF-2 and EGF for tympanic membrane repair, 47 studies were included as of January 30, 2021 [479]. The results demonstrated that both FGF-2 and EGF could safely and effectively regenerate the tympanic membrane [479]. Notably, EGF exhibited a superior regenerative effect on acute perforation, while the combination of FGF-2 with a biological scaffold showed enhanced reparative outcomes for chronic perforation [479].

### Ocular tissue repair

Corneal wounds caused by trauma, surgery, or disease are quite prevalent, and bFGF has been shown to stimulate the proliferation of corneal epithelial cells, stromal fibroblasts, and ECs. Consequently, numerous clinical applications of bFGF in corneal repair have been carried out. In a clinical study investigating corneal epithelial healing after photorefractive keratectomy with bFGF eye drops, 100 patients were enrolled in a control group supplemented with saline [480]. The results showed that the healing time in bFGF group was significantly shorter than that in control group, with no observed side effects or toxic reactions [480]. In another study comparing sodium hyaluronate to bFGF for treating corneal epithelial abrasions caused by mechanical injury, 30 patients participated [481]. The findings revealed that both wound closure rates and reduction in wound size were greater in bFGF group compared with those in HA group [481]. Corneal tissue is rich in nerve endings. Infection due to herpesvirus, surgery, or damage to ocular nerves can impair corneal innervation and lead to neurotrophic keratitis [482].

NGF promotes neuronal differentiation, survival, and the growth of axons and dendrites in the nervous system, playing a crucial role in corneal homeostasis. In a phase II trial assessing NGF for the treatment of neurotrophic keratitis, 156 patients were enrolled, with a control group receiving vehicle treatment [482]. The rate of corneal healing in bFGF group was significantly higher than that in control group after 4 or 8 weeks of treatment [482]. NGF treatment was well tolerated, with adverse reactions being mild or transient [482]. In another multicenter randomized controlled trial focused on neurotrophic keratitis, NGF group demonstrated greater lesion size reduction and disease inhibition compared with vehicle-treated control group [483]. Additionally, NGF targets retinal ganglion cells, protecting retinal ganglion cells is vital for the treatment of retinal injury diseases. Multiple preclinical studies have shown that NGF protects against damage to retinal ganglion cells in animal studies [484]. A randomized controlled study involving 60 patients with open-angle glaucoma investigated the efficacy of short-term, high-dose rhNGF eye drops [485]. The results indicated that local application of rhNGF was safe and well tolerated; however, no statistically significant short-term neuroenhancement was observed [485]. Furthermore, a phase II trial evaluating rhNGF eye drops for the treatment of RP and CME (NCT02609165) is currently ongoing.

Pigment epithelium-derived factor (PEDF) is a multifunctional protein with antioxidant, antiangiogenic, neuroprotective, and neurotrophic functions. It can inhibit pathological neovascularization in the choroidal epithelium and exert a neuroprotective effect on photoreceptor cells [486]. Therefore, PEDF has good application prospects for retina protection. Numerous preclinical studies have been conducted to evaluate the efficacy of PEDF in animal models of diabetic retinopathy [487] and age-related macular degeneration [488]. Furthermore, due to their small size, low immunogenicity, and cost-effectiveness in production, PEDF fragments have been developed and exhibit certain clinical potential [489].

Current studies have focused on the application of multiple bioactive factors to address various risk factors associated with ocular tissue repair [490]. For example, spermine was conjugated to HA using carbodiimide chemistry based on its ability to enhance permeability, while HA serves to inhibit corneal fibrosis-induced alkali burn. The resulting spermine-modified HA was subsequently employed to functionalize nanoceria, which can inhibit oxidative stress, inflammation, apoptosis, and angiogenesis [491]. This functionalized nanoceria was further utilized for loading IGF-1 to promote cell proliferation and migration, and the results showed that the synthetic effects of spermine-modified HA, nanoceria, and IFG-1 significantly facilitated the repair of corneal alkali burns [491].

## Conclusions and prospects

BAPPs are highly versatile agents for tissue repair due to their ability to modulate the microenvironment through multiple mechanisms, and a range of delivery platforms have been developed to effectively incorporate BAPPs. Furthermore, they can be precisely released in response to endogenous stimuli from the microenvironment as well as exogenous stimuli under artificial control. Taking into account the functions of BAPPs, the characteristics of the tissue microenvironment, and suitable delivery strategies, BAPPs hold great promise in tissue repair.

When utilizing BAPPs for targeted tissue repair, several critical factors should be comprehensively considered: 1) the risk factors present in the tissue microenvironment that impede tissue repair, such as excessive ROS and harmful inflammation, 2) the types of repair cells in the microenvironment and their directional fate, 3) the natural physiology processes involved in specific tissue repair following damage, and 4) the function roles of BAPPs. The demand for BAPPs to promote tissue repair catalyzed advancements in microenvironment modulation; however, specific molecular mechanisms and corresponding novel or combined strategies require further investigation. For rational delivery of BAPPs, key considerations include: 1) biocompatibility, 2) degradability, 3) modifications to delivery platforms, 4) combinations of distinctive delivery platforms, 5) release profiles, and 6) the need for stimuli-responsive release. Although BAPPs coupled with their delivery platforms exhibit significant potential for tissue repair, there are still some limitations. Enhancing the resistance of BAPPs to proteases while preserving their biological activity remains a challenge due to their relatively short half-lives. Alternatively, chemical reagents may reduce the functionality of BAPPs during incorporation into delivery platforms, necessitating protective measures to safeguard their bioactivity. Moreover, identifying active sites on BAPPs is essential for enabling rational modification or coupling with delivery platforms.

Given the complexity of tissue repair requirements, current research is progressively evolving from single-factor incorporation to multifactor delivery systems. A variety of two-factor systems have been extensively investigated, but these systems are insufficient to fully meet complex needs. Therefore, robust delivery platforms and reasonable combinations of various delivery platforms need to be established for the delivery of multiple BAPPs. Additionally, on-demand release mechanisms of BAPPs can be developed to accommodate intricate repair needs. Novel stimuli-responsive release strategies still need further exploration, and synergistic response systems that utilize multiple stimuli should be designed to facilitate the on-demand release of distinct BAPPs. Moreover, co-delivery of multiple BAPPs can be achieved through a combination of sustained release and stimuli-responsive mechanisms. Furthermore, improving the targeted delivery of BAPPs as opposed to relying on free diffusion may be a potential direction for advancing tissue repair.

BAPPs can be delivered in conjunction with other components, such as active ions and small molecule drugs, to enhance tissue repair. Additionally, biophysical stimuli, including temperature, low-intensity pulsed ultrasound, and electric field, can regulate cellular behavior and facilitate tissue repair. These stimuli can also be precisely controlled on demand at different times. The synergistic administration of BAPPs alongside biophysical stimuli demonstrates significant potential for tissue repair. Furthermore, BAPPs can collaborate with exogenous stem cells for tissue repair while also recruiting endogenous stem cells.

Further investigations should prioritize the clinical translation of BAPPs in conjunction with their delivery platforms. Bioactive peptides comprising 5—50 amino acids may offer greater promise than bioactive proteins for tissue repair, owing to advantages such as simplified synthesis and purification, cost-effectiveness, enhanced stability, and high selectivity. Consequently, there is a pressing need for the clinical translation of bioactive peptides along with their development, design, screening, and optimization for tissue repair. Standardized protocols and large animal models should be established to evaluate their biosafety and efficacy comprehensively. The mass production of BAPPs alongside their delivery platforms is essential to reduce medical costs. This necessitates advancements in corresponding preparation technologies. Personalized and precision treatment strategies utilizing BAPPs can enhance therapeutic effectiveness while minimizing adverse effects. Ultimately, BAPPs are potential and robust agents for tissue repair in the future, with an increasing number likely to gain approval for use in clinical tissue regeneration.

## Data Availability

Not applicable.

## Abbreviations

AMF: Alternating magnetic field
AMPs: Antimicrobial peptides
AOPs: Antioxidant peptides
ASIC: Acid-sensing ion channel
BAPPs: Bioactive peptides and proteins
BDNF: Brain-derived neurotrophic factor
BMPs: Bone morphogenetic proteins
BMSCs: Bone marrow mesenchymal stem cells
BPNTs: Black phosphorus nanosheets
BSA: Bovine serum albumin
CCL: C chemokine ligand
CGRP: Calcitonin gene-related peptide
CNTs: Carbon nanotubes
DAXX: Death-domain associated protein 6
E7: EPLQLKM
ECM: Extracellular matrix
ECs: Endothelial cells
EPD: Electrophoretic deposition
ET-1: Endothelin-1
ETBR: Endothelin B receptor
Ex-4: Exendin-4
FGFs: Fibroblast growth factors
FN: Fibronectin
FSP1: Ferroptosis suppressor protein 1
GM-CSF: Granulocyte–macrophage colony-stimulating factor
GO: Graphene oxide
GOx: Glucose oxidase
GST: Glutathione-S-transferase
HA: Hyaluronic acid
HBD: Human β-defense
HB-EGF: Heparin-binding epidermal growth factor-like growth factor
HDAC: Histone deacetylase
HNTs: Halloysite nanotubes
IFN-γ: Interferon-γ
IGF: Insulin-like growth factor
IGFBP7: IGF binding protein 7
IL: Interleukin
KGF: Keratinocyte growth factor
LBL: Layer-by-layer
LCST: Lower critical solution temperature
LDH: Layered double hydroxide
LPS: Lipopolysaccharide
MAPK: Mitogen-activated protein kinase
MCP-1: Monocyte chemotactic protein-1
MLIF: Monocyte locomotion inhibitory factor
MMPs: Matrix metalloproteinases
MOFs: Metal-organic frameworks
MSNs: Mesoporous silica nanoparticles
mTOR: Mammalian target of rapamycin
NELL-1: Nel-like molecule-1
NGF: Nerve growth factor
NIR: Near-infrared ray
NSCs: Neural stem cells
NT-3: Neurotrophin-3
OGP: Osteogenic growth peptide
PBA: Phenylboronic acid
PCL: Poly(ε-caprolactone)
PDA: Polydopamine
PDGF: Platelet-derived growth factor
PEDF: Pigment epithelium-derived factor
PEG: Polyethylene glycol
PFC: Perfluorocarbon
PHSRN: Proline-histidine-serine-arginine-aspartic acid
PI3K: Phosphatidylinositol 3-kinase
PLA: Poly(lactic acid)
PLGA: Poly(lactic-co-glycolic acid)
PLL: Poly(L-lysine)
pNIPAM: Poly(N-isopropyl acrylamide)
PTh: Polythiophene
PVA: Poly(vinyl alcohol)
PPy: Polypyrrole
RGD: Tripeptide arginine-glycine-aspartic acid
rGO: Reduced GO
ROS: Reactive oxygen species
SDF-1: Stromal cell-derived factor-1
sHA: Sulfated HA
SMF: Stationary magnetic field
TCR: T-cell antigen receptor
TGF-β: Transforming growth factor-β
Ti: Titanium
TK: Thioester
TNF: Tumor necrosis factor
TNTs: TiO_2_ nanotubes
t-ZnO: Tetrapod zinc oxide
VEGF: Vascular endothelial growth factor
VIP: Vasoactive intestinal peptide
VSMCs: Vascular smooth muscle cells
